# Nanomaterials with dual immunomodulatory functions for synergistic therapy of breast cancer brain metastases

**DOI:** 10.1016/j.bioactmat.2023.04.021

**Published:** 2023-04-27

**Authors:** Zhenhao Zhao, Chufeng Li, Yiwen Zhang, Chao Li, Yongchao Chu, Xuwen Li, Peixin Liu, Hongyi Chen, Yu Wang, Boyu Su, Qinjun Chen, Tao Sun, Chen Jiang

**Affiliations:** Department of Pharmaceutics, School of Pharmacy, Fudan University, Key Laboratory of Smart Drug Delivery, Ministry of Education, State Key Laboratory of Medical Neurobiology and MOE Frontiers Center for Brain Science, Shanghai, 201203, China

**Keywords:** Breast cancer brain metastases, Drug delivery, Immunogenic cell death, Microenvironmental regulation, STAT3

## Abstract

A long-standing paucity of effective therapies results in the poor outcomes of triple-negative breast cancer brain metastases. Immunotherapy has made progress in the treatment of tumors, but limited by the non-immunogenicity of tumors and strong immunosuppressive environment, patients with TNBC brain metastases have not yet benefited from immunotherapy. Dual immunoregulatory strategies with enhanced immune activation and reversal of the immunosuppressive microenvironment provide new therapeutic options for patients. Here, we propose a cocktail-like therapeutic strategy of microenvironment regulation-chemotherapy-immune synergistic sensitization and construct reduction-sensitive immune microenvironment regulation nanomaterials (SIL@T). SIL@T modified with targeting peptide penetrates the BBB and is subsequently internalized into metastatic breast cancer cells, releasing silybin and oxaliplatin responsively in the cells. SIL@T preferentially accumulates at the metastatic site and can significantly prolong the survival period of model animals. Mechanistic studies have shown that SIL@T can effectively induce immunogenic cell death of metastatic cells, activate immune responses and increase infiltration of CD8^+^ T cells. Meanwhile, the activation of STAT3 in the metastatic foci is attenuated and the immunosuppressive microenvironment is reversed. This study demonstrates that SIL@T with dual immunomodulatory functions provides a promising immune synergistic therapy strategy for breast cancer brain metastases.

## Highlights

• We propose a cocktail-like strategy for synergistic therapy of breast cancer brain metastases.
• Reduction-sensitive nanomaterials can penetrate BBB and concentrated drug release within metastatic foci.
• SIL@T can enhance immune activation and reverse immunosuppressive in brain metastases.
• SIL@T enables the synergistic immunotherapy of brain metastases.

## Graphical abstract

**Figure:**
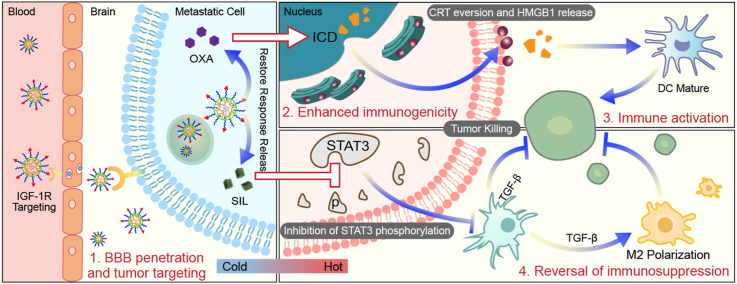

## 1 Introduction

Brain metastases (BM) are the most common type of endogenous brain tumors, occurring in approximately 30% of patients with metastatic breast cancer (BC) [[1], [2], [3]]. Among BCs, up to 46% of patients with triple-negative breast cancer (TNBC) will develop brain metastases and exhibit the worst therapeutic outcomes because of the paucity of effective therapies (the median survival is only 4.9 month) [4]. The treatment of TNBC brain metastases is largely palliative, but neither conventional chemoradiotherapy nor surgery has achieved obvious curative effect due to the existence of blood-brain barrier (BBB) and multiple metastases [5,6]. There is currently no effective clinical treatment and it is urgent to find new treatment strategies.

Due to the potential of the immune system among various novel therapeutic strategies, immunotherapy has drawn the attention of researchers [[7], [8], [9]]. Immune-based strategies, however, have been limited to date by the traditional notion that BC is immunologically cold or minimally immunogenic [10]. Approaches to reprogram tumor microenvironment (TME) to convert cold tumors into hot tumors and thus improve the efficacy of immunotherapy are gradually being developed [11,12]. Of concern, however, is that the central nervous system (CNS) is traditionally considered an immune privileged site without most peripheral immune cells [13]. Combined with limited penetration of conventional drugs into the brain, patients with BMs are excluded from many clinical trials involving immunotherapies (IT), limiting current data related to IT for BCBM treatment [14]. While there is clear evidence that T cells do infiltrate BCBMs and lower the accumulation of tumor infiltrating lymphocytes (TILs) in BM [[15], [16], [17]]. Besides activation of the immune response *via* immunogenic cell death (ICD) prolongs survival in mice with brain metastases [[18], [19], [20]], suggesting that enhance T cell trafficking to BCBMs may be a valid strategy for enhancing efficacy.

While the presence of TILs is often correlated with better prognosis and indicates higher response rates to immunotherapy, the presence of immunosuppressive components in TME is associated with tumor promotion and therapy resistance [21]. Establishment of the brain micrometastases requires reactive, inflammatory components including early infiltration and reprogramming of various immune cells and astrocytes within the brain as a “pre-metastatic niche” [22]. Signal transducer and activator of transcription 3 (STAT3) is abnormally activated in tumor cells and astrocytes of BCBM, and promotes the synthesis and secretion of downstream cancer-promoting signal molecules *via* intercellular signaling, thus inducing the M2 polarization of tumor-associated macrophages (TAM), inhibiting the infiltration of CD8^+^ T cells, and forming the immunosuppressive metastatic microenvironment [23,24]. Silibinin (SIL), a STAT3 inhibitor targeting these signaling axes in BM, has been shown to improve BCBM outcomes [25]. Microenvironmental regulatory strategies to reverse immunosuppression by inhibiting STAT3 phosphorylation therefore represent an intriguing target for BM treatment.

**Scheme 1:**
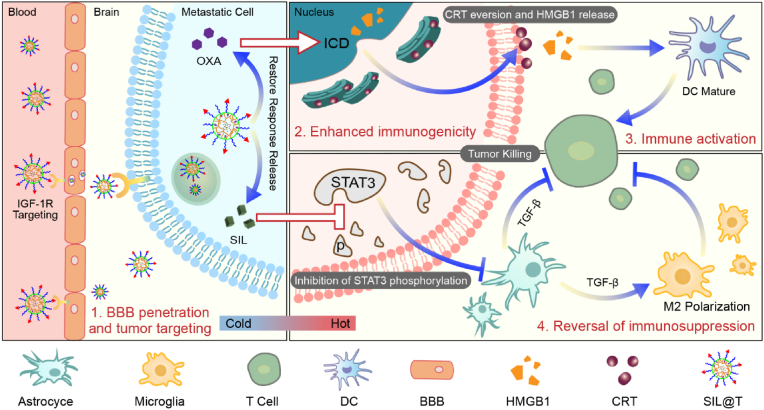
Schematic illustration of SIL@T with dual immunomodulatory functions for the treatment of breast cancer brain metastases.

In this study, based on the cocktail-like strategy for microenvironment regulation-chemotherapy-immune synergistic sensitization involving BBB targeting and concentrated drug release within BCBMs, we constructed nanomaterials (SIL@T) with dual immunomodulatory functions to deliver SIL for reversing the immunosuppressive microenvironment and Oxaliplatin (OXA) for increasing the infiltration of TILs (Scheme 1). Anchored with the CSKC optimized by ligandanalogs of insulin-like growth factor 1 receptor (IGF-1R), SIL@T can penetrate the BBB and subsequently target the brain metastases. Upon internalization by metastatic tumor cells, the micellar structure was destroyed, the encapsulated SIL was leaked to inhibit STAT3 phosphorylation, and OXA was released responsively to induce ICD in the highly reduced cytoplasmic environment. The pharmacodynamic results in the TNBC brain metastasis model mice (BM-mice) also showed that SIL@T could stimulate dendritic cells (DC) maturation and increase CD8^+^ T cell infiltration in the metastatic area, while inhibiting the activation of STAT3 in metastatic cells to reverse the immunosuppressive metastatic microenvironment, significantly prolonging the survival time of BM-mice.

## 2 Materials and methods

### 2.1 Materials and animals

*N*_*6*_-cbz-*l*-Lysine (Lys(*Z*)), Hydrogen bromide 33 wt% in Acetic acid (HBr/HOAc, 33%), HATU, DIPEA, D_2_O (99.8%), BSA were from J＆K Scientific (Shanghai, China). *L*-phenylalanine, triphosgene were from TCI (Asakawa, Japan). Azido-PEG-NHS ester (Mw = 5 kD) was purchased from JenKem (Beijing, China). Methoxy-PEG-amine (CH_3_O-PEG-NH_2_, Mw = 5 kD) was from Seebio Biotech (Shanghai, China). DCM, THF, DMF were from Acros Organics (State of New Jersey, USA). Oxaliplatin, heparin sodium, wortmannin (WTM), *D*-luciferin potassium salt, glutathione (GSH), cell counting kit-8(CCK-8) were purchased from Meilunbio (Dalian, China). Cuprous iodide (CuI), dimethyl sulfoxide-*d*_*6*_ (DMSO-*d*_*6*_ (D, 99.8%), TMS (0.03%)) were from Energy Chemical (Shanghai, China). Succinic anhydride (99%), sodium ascorbate, trifluoroacetic acid (TFA), pyrene, HEPES were from Aladdin Chemistry (Shanghai, China). *D*-type CSKC peptide was purchased from QYAOBIO (Suzhou, China). SnakeSkin™ dialysis tubing (MWCO = 3.5, 5, 8 kD), DAPI (No. D3571) were from Thermo Scientific (Waltham, USA). BODIPY-NHS 630/650 was from Lumiprobe (Hunt Valley, USA). Gelatin, filipin, chlorpromazine, poly-*D*-lysine (PDL, Mw = 70 kD), dimethyl sulfoxide for cellular use were from Sigma Aldrich (Saint Louis, USA). DMEM, FBS, pen-strep (100 × ), *L*-glutamine solution (100 × ), MEM NEAA (100 × ), trypsin-EDTA solution (0.25%), trypsin solution (0.25%), B-27™ supplement (50 × ), goat serum were from Gibco (Carlsbad, USA). Isoflurane was from RWD (Shenzhen, China). Phospho-STAT3 (Tyr705) (Y705) monoclonal antibody (AP0070) was from ABclonal Technology Co., Ltd. (Wuhan, China). All other reagents were analytically pure and from, Sinopharm Chemical Reagent (Shanghai, China).

Mouse-derived triple-negative breast cancer brain metastasis-prone cell line 4T1-Br was kindly donated by Jiangbing Zhou, Professor of Biomedical Engineering at Yale University. A stable transduced murine-derived triple-negative breast cancer brain metastasis-prone cell line 4T1-Br/Luc carrying a luciferase reporter gene was constructed and screened with CMV-Luc-PGK-Puro lentivirus from 4T1-Br cell line by Genomeditech (Shanghai, China).

SPF grade c57 nude mice (female, 6–8 weeks old, weight 18–20 g), SPF grade c57 suckling mice (female, within 24 h or 1–3 days after birth) were purchased from SLAC Laboratory Animal Co., Ltd (Shanghai, China). All animal experiment operations have been approved by the experimental animal ethics committee of the school of pharmacy of Fudan University and follow relevant management regulations.

### 2.2 Preparation, characterizations and formulation optimization of the micelles

#### 2.2.1 Preparation and formulation optimization of the micelles

The micelles were prepared by a dialysis method. The polymer materials (PEG-*p*Lys/OXA-*p*Phe or CSKC-PEG-*p*Lys/OXA-*p*Phe, the synthesis method is described in supporting information) were dissolved in DMSO at a concentration of 10 mg/mL and were sealed in the dialysis bag (MWCO = 3.5 kD. Then they were dialyzed in deionized water for 24 h with three water changes. The liquid in the dialysis bag was collected, and the particle size distribution and zeta potential of the micelles were measured using Malvern3600 Zetasizer Nano-ZS laser particle size analyzer (Malvern Panalytical, Worcestershire UK).

#### 2.2.2 The redox-sensitive properties of polymeric micelles

The sensitivity of polymeric micelles was investigated using PBS (pH = 7.4) buffer containing 10 mM Vc to simulate the cytoplasmic microenvironment and PBS (pH = 5.5) buffer containing 2 mM Vc to simulate the lysosomal microenvironment. The drug release from the micelles was investigated by high performance liquid chromatography (HPLC) at different time points (OXA detection method: column: Agilent C18, 250 mm × 4.6 mm, 5 μm; Mobile phase: 10% MeOH-90% H_2_O; Flow rate: 1 mL/min; injection volume: 10 μL; Detector and detection wavelength: VWD, 250 nm) (SIL detection method: column: Agilent C18, 250 mm × 4.6 mm, 5 μm; Mobile phase: 50% ACN-50% NaH2PO4 (0.1 M, pH = 4.8); Flow rate: 1 mL/min; Injection volume: 10 μL; Detector and detection wavelength: VWD, 288 nm).

### 2.3 Uptake by cells

#### 2.3.1 Uptake of the micelles by 4T1-Br cells

The 4T1-Br cells were inoculated in six-well plates at a density of 1 × 10^5^/well, and when the cells reached 80–90% fusion (typically 18–24 h), the medium was replaced with 2 mL of basal DMEM medium containing dual fluorescent markers (BODIPY-labeled micelles, coumarin-6 used to mimic being encapsulated in hydrophobic core of SIL), SIL@NT or SIL@T in basal DMEM medium (equivalent to 4 μM SIL administered per well). The drug-containing basal medium was removed after 1 h incubation in a 37 °C incubator. And the cells were digested and collected, and 4T1-Br cell uptake was detected using a CytoFLEX S flow cytometer.

#### 2.3.2 Investigation of the uptake pathway of CSKC-modified targeting micelles

The uptake pathway of CSKC-modified targeting micelles SIL@T was investigated using 4T1-Br cells. 4T1-Br cells were inoculated in six-well plates at a density of 1 × 10^5^/well and cultured until fusion reached 80–90% (typically 18–24 h). Each well was pretreated with a different endocytosis inhibitor for 1 h (niche protein endocytosis inhibitor filipin, 5 μg/mL; grid protein endocytosis inhibitor chlorpromazine, 5 μg/mL; giant cell drinking inhibitor Wortmannin, 1 μg/mL). Then the inhibitor-containing medium was removed and DMEM basal medium containing BODIPY-labeled SIL@T was added (each well was administered at a dose equivalent to 4 μM SIL). Then the cells were incubated in an incubator at 37 °C for 0.5 h. One sample (untreated with endocytosis inhibitor) was incubated in a refrigerator at 4 °C for 0.5 h. Cells were washed 3 times with Hank's, digested and collected, and 4T1-Br cell uptake was detected using a CytoFLEX S flow cytometer.

#### 2.3.3 In *vitro* BBB penetration of CSKC-modified targeted micelles

1) BCEC were inoculated at a density of 5*10^4^ cells/cm2 in a 2% gelatin pre-coated 24-well plate transmembrane insert for 14 days; 2) An appropriate amount of culture medium was added to the donor pool so that a liquid level difference of greater than 5 cm could be formed between the donor and recipient pools. Then the cells were incubated for 4 h. If the donor and recipient pools still maintain a significant liquid level difference, BBB formation is tentatively identified; 3) The culture medium was removed and 250 μL of basal DMEM medium containing 0.6 μCi 14C-sucrose, BODIPY-labeled SIL@NT or SIL@T (each well was administered at a dose equivalent to 4 μM SIL) was added separately to each well. 4) The plate was placed in a shaker at 37 °C with 50 rpm shaking and sampled 20 μL at 5, 10, 15 and 30 min; 5) The samples were divided into two parts with one for measuring the 14C-sucrose (add scintillation solution, mix and leave overnight, and measure the radioactivity count by liquid flash counter), and the other one for measuring the BBB permeation of micelles.

### 2.4 In *vivo* targeting of SIL@T to brain metastases

#### 2.4.1 Establishment of the BM-mice

The BM-mice were established by ultrasound-guided left ventricular injection technique with reference to the literature method [26]. After 1 week of modeling, each c57 mouse was injected intraperitoneally with 100 μL of saline containing 30 mg/mL of potassium D-fluorescein, and the IVIS Spectrum (Small Animal In Vivo Optical Imaging System) was used to monitor the bioluminescence signal of 4T1-Br/Luc cells and confirm the successful establishment of the BM-mice.

#### 2.4.2 Targeting ability of SIL@T to TNBC brain metastases

BM-mice with comparable brain metastasis Luc signals were selected and randomly divided into 2 groups of 3 animals each, and BODIPY-labeled micelles SIL@NT and SIL@T (dose: 0.5 mg BODIPY/kg) were injected *via* tail vein, respectively. The internal distribution of micelles was investigated by live imaging of small animals at different time points.

Double fluorescence-labeled micelles SIL@NT and SIL@T were obtained by encapsulating coumarin-6 with BODIPY-labeled micelles. Brain tissue was taken out 2 h after tail vein injection (dose: 0.5 mg BODIPY/kg) and frozen sections were prepared (light-protected operation). Neovascularization was labeled using Anti-CD31antibody (1:50 dilution) and goat anti-rabbit IgG H&L (Alexa Fluor® 568) secondary antibody (1:1000 dilution), and nuclei were labeled with DAPI. The prepared tissue samples were imaged and observed using an inverted laser confocal microscope.

### 2.5 Investigation of in *vitro* cytotoxicity of micelles on TNBC

The toxicity of micelles to TNBC cells 4T1-Br was detected using the CCK-8 kit. Refer to the kit instructions for the specific method.

### 2.6 In *vitro* evaluation of the efficacy of SIL@T

#### 2.6.1 Experimental grouping of formulations

The formulations include: G1: PBS; G2: OXA; G3: SIL@CPLP (where CPLP stands for the targeted polymeric material without OXA attached and SIL@CPLP stands for a micelle constructed from CPLP loaded with SIL.); G4: T (where T stands for a micelle constructed from the targeted polymeric material attached to OXA.); G5: SIL@NT (where NT stands for a micelle constructed from the non-targeted polymeric material attached to OXA. SIL@NT represents the loading of SIL into NT.); G6: SIL@T (where T stands for a micelle constructed from the targeted polymeric material attached to OXA. SIL@T represents the loading of SIL into T.). Among them, G1 was used as a blank control; G4 was a targeted prodrug form of G2 to demonstrate that T could enhance the stability of OXA as well as increase its accumulation at the target site; G5 was a non-targeted form of G6 to confirm that modification of CSKC could help micelles to better cross the BBB and further target to metastases; G3 and G4 were single-drug controls of G6 to demonstrate that the combination of the two drugs could achieve better metastatic tumor treatment by a cocktail-like strategy.

#### 2.6.2 In *vitro* therapeutic effect of SIL@T

Cultures of primary astrocytes (24 h) and complete DMEM cultures were mixed 1:1 for the culture of 4T1-Br-Luc cells.When the cell confluence reached 80–90%, different preparations (100 μmol per group of OXA; 80 μmol per group of SIL) were given and incubated with the cells for 4 h, and then replaced with fresh medium to continue culturing for 72 h.

When using WB to analyze the expression of pSTAT3 protein, the cell was lysed in RIPA buffer containing protease inhibitor. The lysate solution was centrifuged at 12,000 g for 10 min at 4 °C and the supernatant was collected to obtain the protein. The protein concentrations were quantitated by the BCA assay. Then, 20 μg of each protein sample was separated on an SDS-PAGE gel (PowerPacTM Basic Electrophoresis, Bio-Rad, Hercules, CA, USA) and transferred onto PVDF membranes. After being blocked with 5% skimmed milk in tris-buffered saline with 0.1% Tween-20 (TBST), the membranes were probed with Anti-pSTAT3 (1:2000 dilution) at 4 °C overnight. Following incubation of the corresponding IgG-horseradish peroxidase (HRP) conjugated secondary antibodies, specific bands were visualized using immobilon western chemiluminescent HRP substrate.

When using laser confocal live cell imaging system to observe the CRT and HMGB1, use 4% paraformaldehyde to fix the cells. Use Anti-CRT/HMGB1 primary antibody and goat anti-rabbit IgG H&L (Alexa Fluor® 555) secondary antibody (1:1000 dilution) to fluorescently label CRT/HMGB1 protein. And then use DAPI (5 μg/mL) to counter-stain the nuclei for 15 min.

### 2.7 In *vivo* evaluation of the efficacy of SIL@T on brain metastases from TNBC

#### 2.7.1 Administration

On the seventh day after modeling (i.e., grouping day −6), brain metastasis Luc signals were monitored by IVIS Spectrum Small Animal Live Imaging System and BM-mice were randomly divided into 6 groups according to the signals. On days 1, 5 and 9 after grouping, each group was injected *via* tail vein with a dose of OXA equivalent to 5 mg/kg and a dose of SIL equivalent to 6 mg/kg.

#### 2.7.2 Survival status, brain metastasis signal and body weight monitoring in BM-mice

The survival of each group of BM-mice was checked daily after drug administration, and the BM-mice were weighed and monitored every 4 days starting from the first day of grouping. GraphPad Prism 8.0 was used to plot the weight change curve, brain metastasis signal change curve and survival curve, and one-way ANOVA was used to analyze the weight curve and brain metastasis signal curve, and survival test was used to analyze the survival curve and compare the differences between groups.

### 2.8 Investigation of the therapeutic mechanism of SIL@T on TNBC brain metastases

Successfully established BM-mice were given formulations of each group on days 1, 5, and brain tissue was taken out on day 6 for flow cytometry and preparation of frozen sections to investigate the mechanism of SIL@T treatment of brain metastasis, and specific experimental operation reference support information.

### 2.9 HE staining

Tissues of the heart, liver, spleen, lung, and kidney of BM-mice in 2.7 were taken out. Paraffin sections (5 μm thick) were prepared and stained using the HE staining kit. Sections were placed under an inverted fluorescent microscope in bright field for observation.

### 2.10 Statistical analysis

All data were analyzed by GraphPad Prism Version 8.0 (GraphPad Software, San Diego, USA) and presented as means ± standard deviation (SD). The significance level was defined as *P < 0.05, **P < 0.01, ***P < 0.001 and ****P < 0.0001.

## 3 Results and discussion

**Fig. 1:**
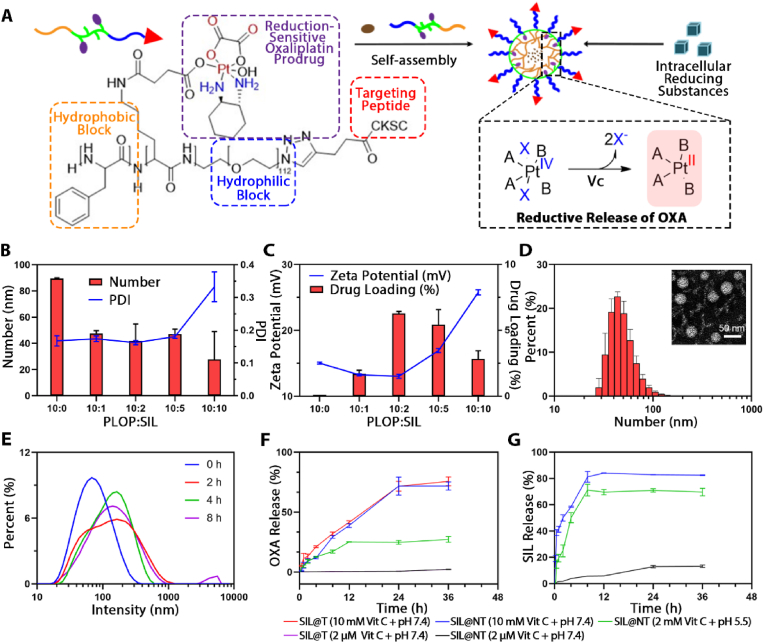
Design, formulation optimization, characterization and release kinetics of SIL@T. A) Design schematic of SIL@T. B) Effects of different mass ratios of polymer materials and SIL on particle size and PDI of micelles. C) Effects of different mass ratios of polymer materials and SIL on the zeta potential of micelles and drug loading of SIL. D) Particle size distribution and TEM images of SIL@T. E) Variation in particle size of micelles in a simulated metastatic tumor cytoplasmic environment. F) Release profile of OXA and SIL(G) at different time under simulated plasma environment, endosome/lysosomal environment, and cytoplasmic environment. Data are presented as means ± SD (n = 3).

In this study, a reduction-sensitive polymer CSKC-PEG-*p*Lys/OXA-*p*Phe (CPLOP) loaded with chemotherapeutic drugs was designed and synthesized as a nanocarrier for encapsulating the microenvironmental regulator SIL (the specific design is shown in Fig. 1A). PEG acts as the hydrophilic segment of the polymer, which can increase the stability, prevent nonspecific adsorption and prolong blood circulation duration. A ring opening polymerization strategy was adopted using PEG-NH_2_ as the initiator to yield the PEGylated polyamino acid block, where phenylalanine was used as a hydrophobic core to facilitate the encapsulation of SIL through π-π interactions and hydrophobic interactions. OXA prodrugs (IV) were coupled with the amino groups of lysine side chains, and can be reduced to the active form (II) of OXA to achieve selective killing of metastatic tumors upon meeting the highly reducing environment in tumor cells. *D*-type CSKC cyclic peptide, a ligand analog of IGF-1R, was linked to the other end of PEG as the targeting moiety to increase BBB penetration and accumulation of metastases.

### 3.1 Synthesis and characterizations of polymers

The synthesis was performed according to the literature (Fig. S1) [26,27]. PEG-*p*Lys-*p*Phe was synthesized by sequential ring-opening polymerization using PEG-NH_2_ as a macroinitiator. OXA prodrug (IV) combines with the amino group of lysine block side chain through electrophilic substitution reaction to obtain polymer PEG-*p*Lys/OXA-*p*Phe (PLOP). CSKC was subsequently modified onto the azide terminus of PEG *via* a click reaction to generate CPLOP. All polymers’ structures were confirmed by ^1^H NMR spectroscopy and the CPLOP was further characterized by gel permeation chromatography (GPC) (Figs. S2–11).

### 3.2 Optimization and characterizations of SIL@T

The SIL-encapsulated micelles were constructed by dialysis method and the formulation was optimized in detail. The effect of the mass ratio of different polymers to SIL on the particle size, potential and drug loading of micelles was studied and shown in Fig. 1B and C. When the mass ratio of polymer PLOP to SIL was optimized to 5:1, the particle size number value measured by dynamic light scattering (DLS) of the micelles (SIL@NT) was 41 ± 10 nm (the intensity value was 78 ± 4 nm), and the drug loading was 6.29 ± 0.16%. The particle size of the micelles after drug encapsulation is smaller than that of the blank micelles, which may be due to the formation of hydrophobic and π-π stacking interactions between the phenylalanine blocks in the polymeric material and SIL, leading to a more tightly packed core with a reduced size. At this ratio, the particle size number value of micelles (SIL@T) prepared using CPLOP was 50 ± 2 nm, which is similar to SIL@NT, indicating that the modification of CSKC peptide has little effect on the particle size (potential and PDI details are in Table S1). The transmission electron microscope (TEM) results of SIL@T showed that the morphology of the micelles was spherical and the particle size was similar to the number value measured by DLS (Fig. 1D). This ratio was used in subsequent experiments to prepare micelles.

### 3.3 Responsive release of SIL@T

To verify the reduction-responsive release of micelles, the drug release behavior of micelles was investigated in simulated plasma environment (2 μM Vit C + pH 7.4), endosome/lysosome environment (2 mM Vit C + pH 5.5) and tumor cytoplasm environment (10 mM Vit C + pH 7.4) *in vitro*. Under the simulated tumor cytoplasmic environment, the size of micelles gradually increased, which was basically the same as the size of empty micelles at 4 h, suggesting that the encapsulated SIL could be effectively released in the cytoplasm (Fig. 1E). In order to further explore the drug release in *vitro*, the percentage of drug release was determined by high performance liquid chromatography (HPLC). In the endosomal/lysosomal environment, the OXA prodrug is partially reduced to the active form due to the presence of a certain concentration of reducing substances. In the simulated high-level intracellular reducing conditions, OXA was basically released in the form of the original drug within 24 h (Fig. 1F). The results showed that OXA prodrugs (IV) could be reduced to the active form (II) of OXA upon meeting the Vit C and the modification of CSKC had no significant effect on drug release. From the release results of SIL, it can be found that micelles can achieve relatively complete drug release in both endosomal/lysosomal and cytoplasmic environments (Fig. 1G). In the simulated cytoplasmic environment, the micelle structure is changed due to the reduction of the OXA prodrug, which in turn leads to the release of the encapsulated SIL. In the acidic environment of endosomes/lysosomes, the amino group of the lysine side chain is protonated, leading to dissociation of the micelles and release of SIL. In summary, it is speculated that the micelles can release SIL in cells to inhibit the phosphorylation of STAT3, and can responsively release the original OXA drug in tumor cells to achieve specific tumor killing. All these speculations as verified in the follow-up pharmacodynamic studies at the cellular level.

### 3.4 Cellular uptake and subcellular distribution of SIL@T *in vitro*

**Fig. 2:**
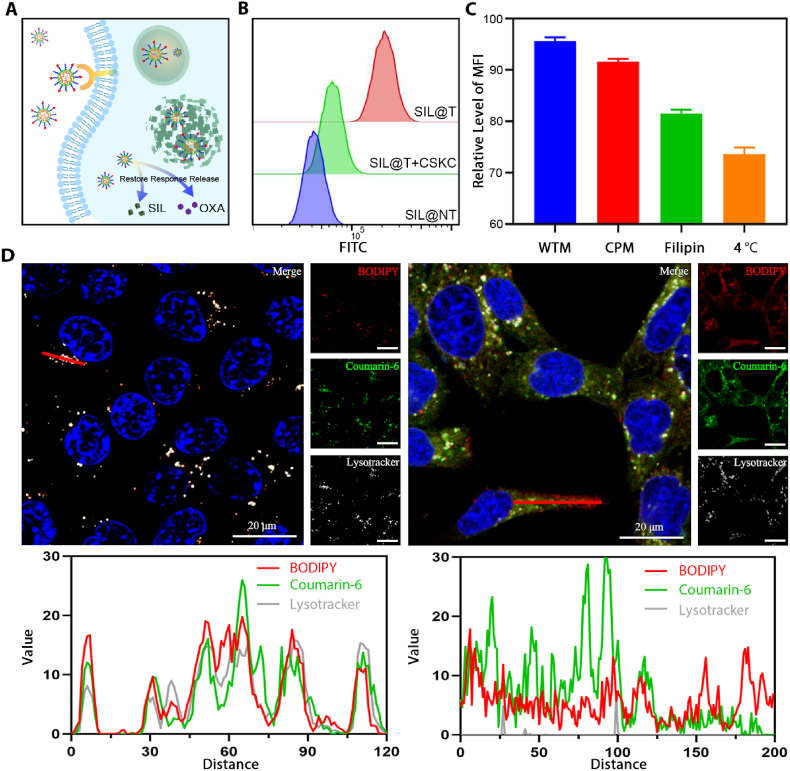
Cellular uptake and subcellular distribution of SIL@T. A) Diagram of the intracellular drug release. B) Flow cytometry results of micellar uptake by 4T1-Br-Luc cells (1 h after formulation administration), FITC: coumarin-6. C) Quantitative results of SIL@T uptake by 4T1-Br-Luc cells after pretreatment with inhibitors of different uptake pathways (1 h after formulation administration). D) Uptake of dual fluorescently labeled SIL@T (BODIOY-labeled polymer, coumarin-6 mimic encapsulated SIL) by 4T1-Br-Luc cells at 0.5 h (left) and 1 h (right). The pixel intensity maps are plotted along the red line. Blue: nucleus; green: coumarin-6; white-grey: endosome/lysosome; red: micelles. Scale bars: 20 μm.

In order to realize the microenvironment regulation-chemo-immune synergistic sensitization therapy of SIL@T as designed, the BBB penetration of micelles and the centralized drug release in metastases are crucial. Therefore, an active targeting strategy was employed to enhance the metastases-enriching ability of micelles while utilizing the long-circulation of PEG and the EPR effect of nanoparticles. IGF-1R is highly expressed on brain capillary endothelial cells and metastatic breast cancer cells (Fig. S12) [26], and modification of its ligand analog CSKC peptide can mediate selective drug delivery through IGF-1R [26]. Based on this, we speculated that SIL@T can effectively cross the BBB and target TNBC brain metastatic cancer cells through the anchored the CSKC peptide, and subsequently the intracellular drug release (Fig. 2A) and BBB penetration ability of SIL@T in *vitro*.

The surficial ligand modification density onto the micelles could tailor the active targeting efficiency [28]. Therefore, the modification degree of CSKC was optimized first. The cellular uptake of micelles with different degrees of CSKC modification by 4T1-Br cells was examined by flow cytometry and confocal laser microscopy (Fig. 2B and Figs. S13–15). The results showed that when the modification degree of CSKC was up to 40%, the cells exhibited the saturated maximum uptake of micelles. But the uptake of tumor cells decreases after the degree of modification is greater than 40%. With the increase of ligand modification, cell uptake generally increases at first and then decreases, which is accused for steric hindrance of closely packed ligand that may inversely deteriorate binding of nanoparticles to target, and consumption of high number of cell membrane receptors which decreases the overall cellular uptake [28]. Based on this, the CSKC modification degree of SIL@T was determined as 40% in the subsequent experiments. To further explore the cellular uptake pathway of SIL@T, 4T1-Br cells were pretreated with different uptake inhibitors, and the uptake of micelles was investigated by flow cytometry after incubation with SIL@T. Cellular uptake was found dramatically inhibited after treatment with filipin, chlorpromazine (CPM), and low temperature, indicating that SIL@T is mainly taken up by cells *via* an energy-dependent caveolin- and clathrin-mediated endocytic pathway (Fig. 2C and Fig. S16). Subsequently, the cellular internalization, subcellular distribution, and endosomal/lysosomal escape of dual fluorescently labeled SIL@T (BODIPY-labeled polymer, coumarin-6 mimetic-encapsulated SIL) were investigated by CLSM (Fig. 2D). Until 0.5 h of cellular uptake, the micelles were basically localized to endosomes/lysosomes, which was consistent with the cellular internalization process corresponding to the SIL@T uptake pathway. After 1 h, the red fluorescence representing micelles and the green fluorescence representing SIL had diffused in the cytoplasm, indicating that SIL@T can rapidly achieve endosomal/lysosomal escape after being taken up by cells. The rapid endosome/lysosome escape may benefit from the proton sponge effect of the protonable amine groups in the polymer under an acidic pH environment.

### 3.5 BBB-crossing ability, metastatic tumor targeting ability and *in vivo* biodistribution of SIL@T

**Fig. 3:**
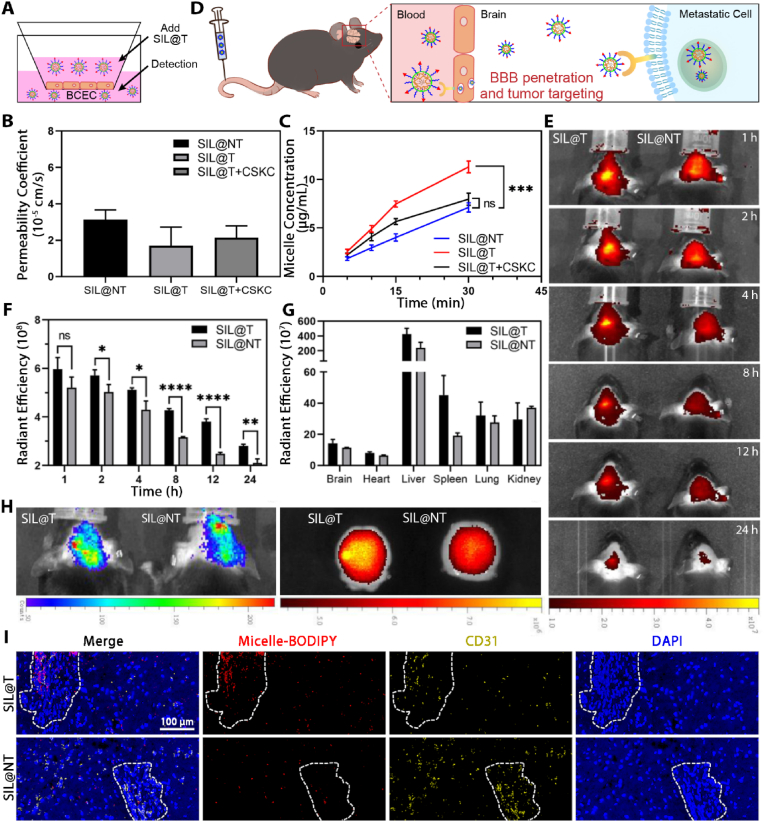
BBB penetration ability and targeting performance of SIL@T. A) Scheme of construction of the BBB model *in vitro*. B) Permeability of the BBB constructed *in vitro* (n = 3). C) BBB penetration results of micelles under different conditions. Results are reported as mean ± SD (n = 3, One-way ANOVA, ***P < 0.001). D) Scheme of SIL@T penetrating BBB and targeting metastatic tumors. E) IVIS imaging results of BM-mice at different time after tail vein injection of BODIPY-labeled micelles. F) Quantitative results in panel E (n = 3, One-way ANOVA, *P < 0.05, **P < 0.01, ****P < 0.0001). G) Tissue distribution of micelles 24 h after tail vein injection of BODIPY-labeled micelles (n = 3). H) Bioluminescence imaging of BM-mice and brain distribution of BODIPY-labeled micelles 24 h after tail vein injection. I) Fluorescence imaging of brain frozen sections of BM-mice 2 h after tail vein injection of BODIPY-labeled micelles. Blue: nucleus; yellow: new blood vessels; red: micelles. Scale bar: 100 μm.

In the BBB model constructed *in vitro*, the effect of CSKC modification on the promotion of micelles across the BBB was further verified. The results in Fig. 3A–C shows that the integrity of the BBB constructed *in vitro* before and after the experiment is well maintained (the permeability coefficient is less than 8 × 10^−5^ cm/s [29]), and the modification of CSKC can effectively enhance the BBB-crossing ability of the micelles. Brain metastasis model mice (BM-mice) were constructed by injecting 4T1-Br-Luc cells into the left ventricle of c57 mice through ultrasound imaging. The accumulation in the brain and biodistribution of BODIPY-labeled micelles in BM-mice (Fig. 3D) were investigated by using the small animal *in vivo* imaging technology. After injection through the tail vein, the accumulation results of micelles in the brain of BM-mice at different times are shown in Fig. 3E and F. Compared with untargeting SIL@NT, the fluorescence signal of SIL@T in the brain is apparently enhanced, indicating that the retention of micelles in the brain is increased. The quantitative results also showed that the accumulation of SIL@T in the brain was significantly increased compared with SIL@NT. 24 h after micelles were injected into the tail vein, the main organs and tissues of BM-mice were excised to explore the distribution of micelles *in vivo* (Fig. 3G, H and Fig. S17). It was found that the accumulation of SIL@T in the brain of BM-mice was specific, and this specific brain distribution was consistent with the bioluminescence signal of metastases, suggesting that SIL@T could effectively target metastases after penetrating into the BBB. To this end, the brains of BM-mice were collected 2 h after the micelles were injected into the tail vein to prepare frozen sections, and the BBB penetration and metastases targeting abilities of the micelles were further investigated. In the metastatic foci, SIL@T had obvious fluorescent signal, and the degree of coincidence with the fluorescent signal of CD31 marking neovascularization was low, indicating that SIL@T could effectively penetrate the BBB and accumulate in the metastatic foci (Fig. 3I). As brain metastases grow, the BBB is gradually remodeled as a blood-tumor barrier and is thought to have better permeability [14]. However, endothelial cells of BTB still retain high transmembrane resistance and a large number of efflux pumps, limiting paracellular and transcellular drug transport, and usually only lipophilic and low-molecular-weight drugs can effectively cross BTB [30]. This corresponds to the result that SIL@NT has only weak fluorescence signal in metastases. Therefore, a "double guaranteed" strategy targeting IGF-1R can increase the accumulation of micelles into the metastases, so that the escorted drug could be endowed with higher opportunities to distribute to metastases.

### 3.6 *In vitro* therapeutic effect of SIL@T

**Fig. 4:**
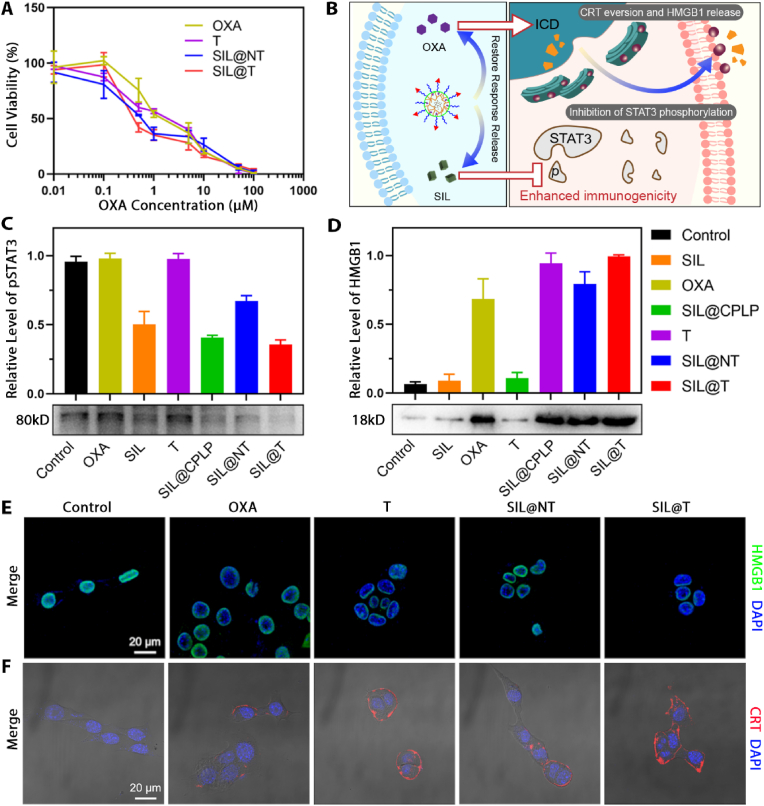
SIL@T can induce ICD and inhibit STAT3 phosphorylation on tumor cells. A) Effects of different preparations on the viability of 4T1-Br-Luc cells (n = 6). B) Scheme of the effect of SIL@T at the cellular level. C) Effects of different preparations on STAT3 phosphorylation in 4T1-Br-Luc cells. D) Investigation on the induction of HMGB1 release from 4T1-Br-Luc cells by different preparations. E) Fluorescence imaging of HMGB1 release from 4T1-Br-Luc cells induced by different preparations. Blue: nucleus; green: HMGB1. Scale bar: 20 μm. F) Fluorescence imaging of CRT translocation induced by different preparations in 4T1-Br-Luc cells. Blue: nucleus; red: CRT. Scale bar: 20 μm.

In SIL@T, SIL is responsible for inhibiting the phosphorylation of STAT3 to regulate the metastatic microenvironment, and OXA is responsible for inducing ICD of tumor cells and thereby enhancing the activation of immune responses while killing tumors. First, the effect of each preparation on tumor cell viability was assessed by cell counting kit-8 (Fig. 4A, and the half-inhibitory concentration (IC_50_) of each group is detailed in Table S2). From the results, the targeted micelles without SIL (T) had lower IC_50_ values than free OXA, which may be related to the poor stability of OXA in aqueous systems. Due to CSKC modification, SIL@T uptake was more than SIL@NT (Fig. 2B), which in turn exhibited a stronger inhibition of cellular activity. Since the interactions in the microenvironment were not involved in the *in vitro* studies, T and SIL@T exhibited similar activity inhibition on tumor cells.

Subsequently, the cocktail-like treatment mechanism of SIL@T (Fig. 4B) was further verified at the cellular level. First, the inhibitory effect of SIL on STAT3 phosphorylation in tumor cells was investigated, and the protein was extracted 72 h after administration of the preparation for western blot (WB) analysis. It was found that all preparations containing SIL could effectively inhibit the phosphorylation of STAT3, and the presence of OXA did not affect the activation of STAT3 in cells (Fig. 4C). SIL@NT lacking CSKC modification has a weaker STAT3 phosphorylation inhibition effect due to lower cellular uptake than SIL@T and micelles without covalently linked OXA but encapsulating SIL (SIL@CPLP). Then the ICD induced by OXA was investigated. It has been previously studied that OXA, as a type I ICD inducer, can initiate the endoplasmic reticulum (ER) stress in tumor cells and further apoptosis. Among them, Calreticulin (CRT), as an abundant protein of ER, and high mobility group protein B1 (HMGB1), as a nuclear protein released during cell death, can be recognized and processed by antigen-presenting cells (APC). Therefore, CRT exposure and HMGB1 release are considered as surrogate markers for ICD [27]. The cell culture medium after administration of the preparation was collected, concentrated and leveled the protein concentration, and the release of HMGB1 was analyzed by WB (Fig. 4D). The results showed that the preparations containing OXA could effectively promote the release of HMGB1 from tumor cells, but the effect was slightly worse than SIL@T due to the poor stability of OXA. Subsequently, the level of HMGB1 in the nucleus and the translocation of CRT were investigated by immune cell imaging technology. Since OXA can kill tumors and promote the release of HMGB1, HMGB1 is lost in the nucleus, which is corroborated with the results of western blotting (Fig. 4E). In the CRT translocation experiments, cells were not permeabilized, so the level of ER translocation to the cell membrane surface can be better characterized. From the results, the CRT exposure of cells after T and SIL@T treatment was more pronounced, which was associated with enhanced OXA stability and increased cellular uptake (Fig. 4F). Since the occurrence of ER stress is related to ROS, depletion of reducing substances by OXA prodrugs also enhances OXA-induced ICD [27]. The above results show that SIL@T can effectively inhibit the activation of STAT3 and induce the ICD of tumor cells, which is expected to realize the regulation of the immune microenvironment and enhance the activation of adaptive immunity.

### 3.7 Therapeutic effect of SIL@T on BM-mice

**Fig. 5:**
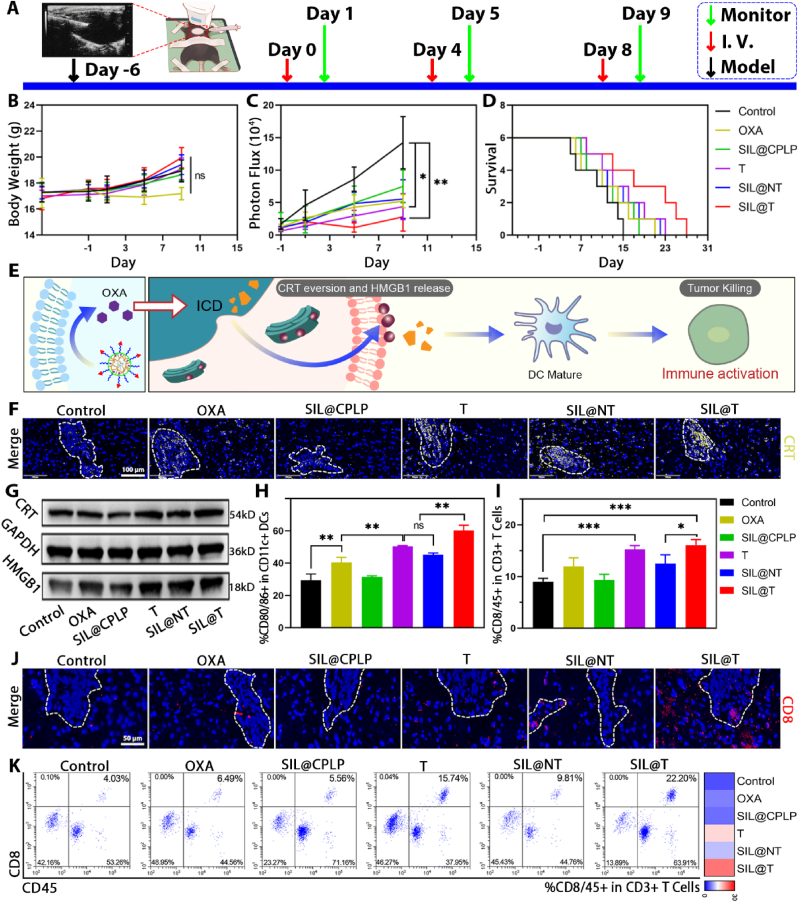
SIL@T can induce ICD, activate immune response, inhibit the growth of metastatic tumors in BM-mice and prolong the survival of BM-mice. A) Treatment and monitor schedule for SIL@T therapy. B) The change curve of body weight after administration of different preparations (n = 6). C) The signal change curve of metastasis tumor after administration of different preparations (n = 4, One-way ANOVA, *P < 0.05, **P < 0.01). D) Survival curves of BM-mice after administration of different formulations (n = 6). E) Scheme of SIL@T activating an immune response. F) Immunofluorescence imaging of CRT in brain cryosections of BM-mice. Blue: nucleus; yellow: CRT. Scale bar: 100 μm. G) WB imaging of metastatic CRT and HMGB1 protein. H) Flow cytometry results of DC maturation in the cervical lymph node of BM-mice (n = 3, One-way ANOVA, **P < 0.01). I) Flow cytometry results of T cell polarization in the spleen of BM-mice (n = 3, One-way ANOVA, *P < 0.05, ***P < 0.001). J) Immunofluorescence imaging of CD8^+^ T cell infiltration in brain cryosections of BM-mice. Blue: nucleus; red: CD8. Scale bar: 50 μm. K) Flow cytometry analysis of T cell polarization in brain metastases.

The *in vivo* antitumor therapeutic effect of SIL@T was evaluated in the mice with 4T1-Br brain metastases. The BM-mice were administered on the seventh day after the establishment, and the weight value and brain metastasis signal of the BM-mice were monitored and recorded one day after the administration, and the survival of the BM-mice was recorded every day (Fig. 5A–D). The signal of brain metastases in the control group BM-mice increased rapidly, and the death started in a relatively short period of time, and the median survival time was only 21 days, indicating that the TNBC brain metastases had a high degree of malignancy and a short median survival time, which was consistent with the actual situation of patients with brain metastases. The remaining treatment groups showed inhibition of metastatic tumor growth and prolonged median survival in model mice (Table S3). Among them, the SIL@CPLP group showed a tumor-suppressing effect because it could achieve the targeted delivery of SIL to metastases and regulate the abnormal signal exchange of metastases [25]. As one of the therapeutic drugs for patients with clinical brain metastases, OXA exhibited a good tumor inhibitory effect on the treatment of model mice. The T (i.e. the targeted micelles without SIL) group increased the accumulation of OXA in the metastases, so the tumor inhibition effect was better, and there was a significant difference compared with the control group. SIL@T takes into account the regulation of the microenvironment and the killing of metastases, so it has the best tumor suppression effect and the longest median survival. With the prolonged treatment time, the body weight of BM-mice in all treatment groups increased slightly, the HE staining images of the main organs showed that there was no obvious organ damage in all groups, indicating that the micelle delivery system was biocompatible and caused no obvious toxicity to the BM-mice (Fig. S18).

### 3.8 The therapeutic mechanism of SIL@T on BM-mice

The construction of SIL@T is to provide a new treatment method for brain metastases through a cocktail-like therapeutic strategy of microenvironmental regulation-chemotherapy-immune synergistic sensitization. Although SIL@T was demonstrated to inhibit STAT3 phosphorylation and induce ICD at the cellular level *in vitro*, the results of this simple system validation may not be applicable to complex *in vivo* environments, especially for the tightly regulated brain. Therefore, the therapeutic mechanism of SIL@T was further explored by flow cytometry, WB analysis, and immunofluorescence staining techniques.

#### 3.8.1 SIL@T induces ICD in metastatic tumors and enhances immune activation (Fig. 5E)

The ICD induced by OXA has been widely studied, but the high aqueous solubility and poor stability lead to relatively rare applications of OXA in intracranial tumors, especially considering that metastases are constrained by the immunosuppressive protection of the brain. From the WB and immunofluorescence results of the immunogenic death markers CRT and HMGB1, OXA can indeed induce the ICD, but the effect is obviously inferior to SIL@T, mainly due to the improved accumulation of OXA in the brain metastases (Fig. 5F, G and Figs. S19 and 20). Antigens can be delivered from the brain to the cervical lymph nodes *via* the olfactory nerve [[31], [32], [33], [34]] (*via* the arachnoid sheath of the olfactory nerves, through the cribriform plate, and to the nasal mucosa) or cerebrospinal fluid [[35], [36], [37], [38], [39]] (the cerebrospinal fluid flows into the brain along arterial perivascular spaces and then translocates into the interstitium *via* aquaporin 4 water channels, before exiting along venous perivascular spaces) [40]. Therefore, to confirm that OXA-induced ICD increases immune activation, the maturation of cervical lymph node DCs and the ratio of spleen CD8^+^ T cells were examined (Fig. 5H, I and Figs. S21 and 22). Compared with the control group, the proportion of mature DC and CD8^+^ T cells in the OXA group increased, and in the preparation group modified with CSKC peptide, the proportion continued to increase. In addition, activated T cells were permitted to enter the CNS parenchyma, therefore, CD8^+^ T cells in metastases were examined [41]. From the results of immunofluorescence, activated T cells could be efficiently recruited to or around metastases (Fig. 5J and Fig. S23). Combined with the results of flow cytometry, it was found that OXA can effectively induce the ICD of metastatic tumor cells and recruit CD8^+^ T cells to the metastases, but the number of its recruitment is relatively small (Fig. 5K and Fig. S24). SIL@T achieves recruitment of effector T cells while ensuring the number of infiltrated T cells. And the number of T cell infiltration is positively correlated with patient prognosis [21], which is also consistent with the best survival outcomes of SIL@T mice. Although little is known about the contribution of ICD to NK cell stimulation, HMGB1 release has been shown to promote NK cell activation [42,43]. This may be related to the fact that HMGB1 can alert the immune system to danger and trigger activation of the immune response [44]. Therefore, we further explored NK cell infiltration to examine the effect of SIL@T on the innate immunity (Fig. S25). It could be found that NK cell infiltration in the metastatic region was increased in the OXA-related preparations group. Since SIL@T increased the accumulation of OXA in the metastatic region, it exhibited the most NK cell infiltration. Therefore, we suggest that SIL@T further enhanced the inhibitory effect on metastatic tumors by increasing the infiltration of NK cells.

#### 3.8.2 SIL@T reverses the immunosuppressive brain metastases microenvironment (Fig. 6A)

**Fig. 6:**
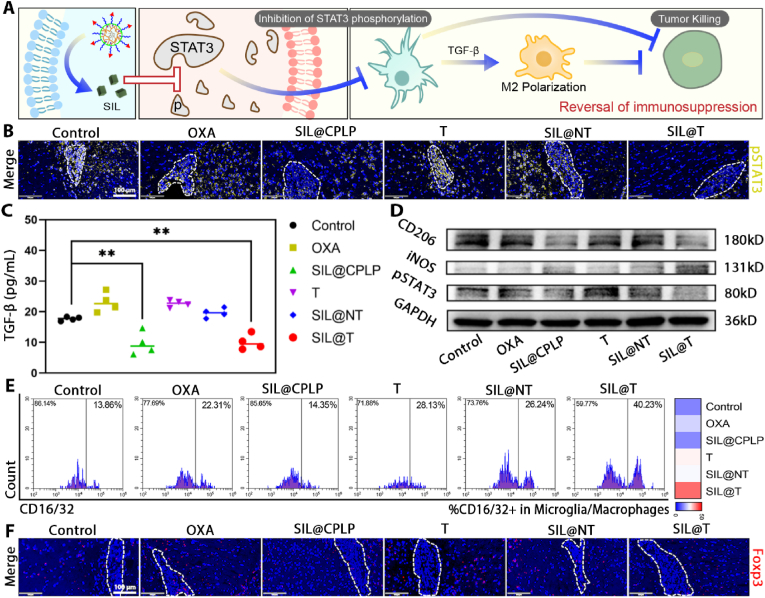
SIL@T can inhibit the phosphorylation of STAT3 and reverse the immunosuppressive microenvironment. A) Scheme of SIL@T reversing immunosuppression. B) Immunofluorescence imaging of pSTAT3 in brain cryosections of BM-mice. Blue: nucleus; yellow: pSTAT3. Scale bar: 100 μm. C) ELISA results of TGF-β content in metastases (n = 4, One-way ANOVA, **P < 0.01). D) WB results of pSTAT3, iNOS and CD206 proteins in metastases. E) Flow cytometry analysis of M1 polarization of TAM in brain metastases. F) Immunofluorescence imaging of CD8^+^ T cell infiltration in brain cryosections of BM-mice. Blue: nucleus; red: Foxp3. Scale bar: 100 μm.

Although the above results have shown that SIL@T can achieve metastatic infiltration of effector T cells, some studies suggest that a strong immunosuppressive microenvironment in the brain will limit the function of effector T cells [45]. STAT3 is abnormally activated in tumor cells and astrocytes, and promotes the synthesis and secretion of downstream cancer-promoting signal molecules *via* intercellular signaling, thus forming the immunosuppressive metastatic microenvironment [23,24]. Specifically, STAT3 is activated in metastatic tumor cells [46], inducing the resistance of tumor cell to chemotherapy, while secreting growth factors induces the STAT3 activation in astrocytes [47,48]. Astrocytes with abnormal activation of STAT3 can secrete MIF and pro-inflammatory factors such as transforming growth factor β (TGF-β) to act on tumor-associated macrophages (TAM) [48,49], thereby leading to M2 polarization of TAM and immunosuppression, and induce the exhaustion of immune cells [23]. Silibinin (SIL), a STAT3 inhibitor targeting these signaling axes in BM, has been shown to improve BCBM outcomes [25]. Therefore, SIL was selected as a microenvironmental modulating drug for improving the microenvironment of immunosuppression (Fig. 6A).

The results of immunofluorescence confirmed that the activation of STAT3 was obvious in the metastatic foci of the BM-mice in the control group, but the activation of STAT3 was inhibited under the treatment of SIL (Fig. 6B and Fig. S26). Meanwhile, ELISA results showed that SIL-containing preparations could reduce the content of TGF-β in metastases, and the reduction of TGF-β could inhibit the M2 polarization of TAMs and Treg cell recruitment (Fig. 6C). Activation of STAT3 leads to the establishment of an immunosuppressive microenvironment [48,49]. Among them, TGF-β and IL-10 are considered to be important components in immunosuppression and contribute to the maintenance of the immunosuppressive microenvironment [50,51]. Therefore, we further explored the changes of IL-10 in the metastatic region. It was possible to find lower levels of IL-10 in the SIL@CPLP and SIL@T groups (Fig. S27), which coincided with the result that STAT3 activation was inhibited in both groups (Fig. 6D and Fig. S28A). This agrees with the results of WB, flow cytometry and immunofluorescence (Fig. 6D–F and Figs. S28–34). M1 TAMs increased, M2 TAMs decreased, and Treg cell infiltration decreased in the metastases of the SIL@T group, confirming that the targeted delivery of SIL therapy could help improve the immunosuppressed brain metastatic microenvironment.

Exploration of the therapeutic mechanism in the BM-mice revealed the mechanism of SIL@T treatment in TNBC brain metastases: OXA-induced ICD directly kills metastatic tumors while activating adaptive immune responses, and SIL reverses the immunosuppressive microenvironment of brain metastases to enhance the immune killing effect on metastatic tumors. Furthermore, by regulating the "cold" tumor immune microenvironment to achieve effective killing of metastatic cells, and ultimately to enhance the synergistic therapeutic effect of TNBC brain metastases.

### 3.9 Therapeutic effect of SIL@T on Co-BM-mice

There are three types of tumor cell inoculation models [52]: local (intracranial), systemic (intracardiac and intracarotid), and orthotopic (intradermic/subcutaneous for melanoma and mammary fat pad for breast cancer). Of these, systemic inoculation recapitulates all steps required for organ colonization. Intracardiac inoculation is the most frequently used approach because of its technical feasibility (i.e. no requirement for invasive surgery, the injection could be guided by ultrasound imaging [53]), high incidence of brain metastasis among mice injected with brain-homing cells, and sufficiently homogeneous time to reach the experimental endpoint [22,54,55]. However, intracarotid inoculation is technically challenging, and requires invasive surgery, which may impact disease pathology. Therefore, we finally chose intracardiac inoculation.

**Fig. 7:**
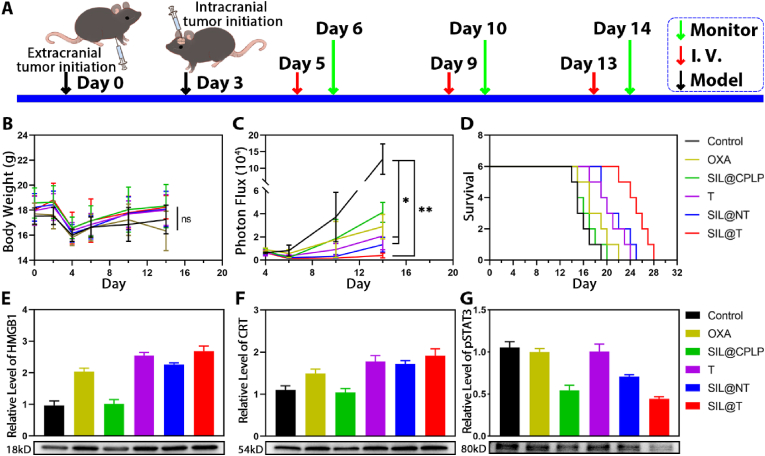
SIL@T can induce ICD, activate immune response, inhibit the growth of metastatic tumors in Co-BM-mice and prolong the survival of Co-BM-mice. A) Treatment and monitor schedule for SIL@T therapy. B) The change curve of body weight after administration of different preparations (n = 6). C) The signal change curve of metastasis tumor after administration of different preparations (n = 4, One-way ANOVA, *P < 0.05, **P < 0.01). D) Survival curves of Co-BM-mice after administration of different formulations (n = 6). E) WB results of metastatic HMGB1. F) WB results of metastatic CRT. G) WB results of metastatic pSTAT3.

Although systemic inoculation is widely accepted, it ignores phenotypic variability (i.e. variable number of metastases per mouse) and the interaction between primary and metastatic tumors. Therefore, a breast cancer transplantation model with intracranial plus extracranial (in situ) tumor (Co-BM-mice), mimicking the clinically observed coexistence of metastases inside and outside the brain, was used to further validate the feasibility of our strategy (model construction is referenced in Ref. [56]). The Co-BM-mice were administered on the fifth day after the establishment, and the weight value and brain metastasis signal of the Co-BM-mice were monitored and recorded one day after the administration, and the survival of the Co-BM-mice was recorded every day (Fig. 7A–D). The control Co-BM-mice showed rapid development of brain metastases and short median survival time, consistent with the clinical presentation of patients with brain metastases. After administration, tumor growth was inhibited and survival was prolonged in all groups of Co-BM-mice (Fig. 7C and D, Table S4). The overall treatment outcome was similar to that of the BM-mice, but improved in the non-targeted group (SIL@NT) because the BBB was disrupted in the Co-BM-mice. The modification of CSKC contributes to the accumulation of the drug at the metastatic site, so the treatment effect is better in SIL@T. To further confirm that SIL@T inhibits metastatic tumor growth through a cocktail-like treatment strategy that inhibits STAT3 phosphorylation and induces ICD, we examined the levels of relevant proteins in the metastatic foci. CRT and HMGB1, markers of immune death, were increased in metastatic region after treatment with OXA-containing agents (Fig. 7E and F). OXA is highly water-soluble and poorly stable, and therefore ICD induction in metastases is poor after administration of free OXA. ICD induction was enhanced after improving OXA accumulation in the tumor by active targeting and prodrug strategies. SIL, as an inhibitor of STAT3 phosphorylation, effectively inhibits STAT3 activation in metastatic region (Fig. 7G). Targeted micelles exhibited optimal STAT3 phosphorylation inhibition because they increased SIL accumulation in metastatic foci. Targeted delivery of OXA and SIL can effectively induce immunogenic death of tumor cells and inhibit activation of STAT3 in metastatic foci (Fig. 7E–G). SIL@T treats brain metastatic tumors through a cocktail strategy of microenvironmental modulation-chemotherapy-immune synergistic sensitization and therefore exhibits optimal therapeutic efficacy.

## 4 Conclusions

We developed a nanomaterial with dual immunomodulatory functions to precisely deliver OXA and SIL to tumor cells in brain metastases through a strategy of active targeting and responsive release for the treatment of BCBM. Reduction-sensitive OXA prodrugs could precisely kill tumor cells and induce immune activation, and SIL could reverse the immunosuppressive microenvironment to enhance the selective killing of metastatic tumors by adaptive immunity. Meanwhile, SIL@T could increase effector T cell infiltration, reduce TAM M2 polarization and Treg cell infiltration in the metastatic foci, so as to achieve the inhibition of metastatic tumors and prolong the median survival of tumor-bearing model mice.

Due to the limitation of brain immune cognition, we did not conduct in-depth exploration of the pathways of peripheral immune activation and effector T cell infiltration. After SIL@T treatment, the proportion of mature DCs in cervical lymph nodes increased, effector T cells were activated in the spleen, and the infiltration of CD8^+^ T cells in brain metastases was improved, all of which supported the immune activation ability of SIL@T against brain metastases. The dual immune regulation strategy and the functional nanomaterials SIL@T developed in this study can provide new ideas for the treatment of tumor brain metastases.

## CRediT authorship contribution statement

**Zhenhao Zhao:** Conceptualization, Methodology, Software, Validation, Formal analysis, Investigation, Data curation, Writing – original draft, Visualization. **Chufeng Li:** Conceptualization, Methodology, Software, Validation. **Yiwen Zhang:** Methodology, Software, Validation. **Chao Li:** Methodology, Software, Validation. **Yongchao Chu:** Methodology, Software, Validation. **Xuwen Li:** Formal analysis, Investigation. **Peixin Liu:** Formal analysis, Investigation. **Hongyi Chen:** Investigation. **Yu Wang:** Investigation. **Boyu Su:** Investigation. **Qinjun Chen:** Investigation. **Tao Sun:** Writing – review & editing, Supervision. **Chen Jiang:** Conceptualization, Methodology, Resources, Writing – review & editing, Supervision, Funding acquisition.

## Declaration of competing interest

The authors declare that they have no known competing financial interests or personal relationships that could have appeared to influence the work reported in this paper.

## Abbreviation

STAT3: Signal transducer and activator of transcription 3
BC: breast cancer
TNBC: triple-negative breast cancer
BBB: blood-brain barrier
CNS: central nervous system
IT: immunotherapy
TIL: tumor infiltrating lymphocyte
ICD: immunogenic cell death
CRT: calreticulin
HMGB1: high mobility group protein B1
TME: tumor microenvironment
TAM: tumor-associated macrophages; OXA, Oxaliplatin
BM-mice: triple-negative breast cancer brain metastasis model mice
CPLOP: CSKC-PEG-*p*Lys/OXA-*p*Phe
PLOP: PEG-*p*Lys/OXA-*p*Phe; ER, endoplasmic reticulum
IGF-1R: insulin-like growth factor 1 receptor
BCEC: brain capillary endothelium
CLSM: confocal laser scanning microscopy
CPM: chlorpromazine
TGF-β: transforming growth factor β

## References

[1] Nussbaum E.S., Djalilian H.R., Cho K.H., Hall W.A. (1996). Brain metastases. Histology, multiplicity, surgery, and survival. Cancer.

[2] Rostami R., Mittal S., Rostami P., Tavassoli F., Jabbari B. (2016). Brain metastasis in breast cancer: a comprehensive literature review. J. Neuro Oncol..

[3] Witzel I., Oliveira-Ferrer L., Pantel K., Müller V., Wikman H. (2016). Breast cancer brain metastases: biology and new clinical perspectives. Breast Cancer Res..

[4] Mounsey L.A., Deal A.M., Keith K.C., Benbow J.M., Shachar S.S., Zagar T., Dees E.C., Carey L.A., Ewend M.G., Anders C.K. (2018). Changing natural history of HER2-positive breast cancer metastatic to the brain in the era of new targeted therapies. Clin. Breast Cancer.

[5] Chamberlain M.C., Baik C.S., Gadi V.K., Bhatia S., Chow L.Q. (2017). Systemic therapy of brain metastases: non-small cell lung cancer, breast cancer, and melanoma. Neuro Oncol..

[6] Ostrom Q.T., Gittleman H., Truitt G., Boscia A., Kruchko C., Barnholtz-Sloan J.S. (2018). CBTRUS statistical report: primary brain and other central nervous system tumors diagnosed in the United States in 2011-2015, neuro. Oncol..

[7] Barzaman K., Moradi-Kalbolandi S., Hosseinzadeh A., Kazemi M.H., Khorramdelazad H., Safari E., Farahmand L. (2021). Breast cancer immunotherapy: current and novel approaches. Int. Immunopharm..

[8] Riley R.S., June C.H., Langer R., Mitchell M.J. (2019). Delivery technologies for cancer immunotherapy. Nat. Rev. Drug Discov..

[9] Yang Z., Gao D., Zhao J., Yang G., Guo M., Wang Y., Ren X., Kim J.S., Jin L., Tian Z., Zhang X. (2023). Thermal immuno-nanomedicine in cancer. Nat. Rev. Clin. Oncol..

[10] Emens L.A. (2018). Breast cancer immunotherapy: facts and hopes. Clin. Cancer Res..

[11] Yang Z., Gao D., Guo X., Jin L., Zheng J., Wang Y., Chen S., Zheng X., Zeng L., Guo M., Zhang X., Tian Z. (2020). Fighting immune cold and reprogramming immunosuppressive tumor microenvironment with red blood cell membrane-camouflaged nanobullets. ACS Nano.

[12] Wang Y., Gao D., Liu Y., Guo X., Chen S., Zeng L., Ma J., Zhang X., Tian Z., Yang Z. (2021). Immunogenic-cell-killing and immunosuppression-inhibiting nanomedicine. Bioact. Mater..

[13] Muldoon L.L., Alvarez J.I., Begley D.J., Boado R.J., Del Zoppo G.J., Doolittle N.D., Engelhardt B., Hallenbeck J.M., Lonser R.R., Ohlfest J.R., Prat A., Scarpa M., Smeyne R.J., Drewes L.R., Neuwelt E.A. (2013). Immunologic privilege in the central nervous system and the blood-brain barrier. J. Cerebr. Blood Flow Metabol..

[14] Carney C.P., Pandey N., Kapur A., Woodworth G.F., Winkles J.A., Kim A.J. (2021). Harnessing nanomedicine for enhanced immunotherapy for breast cancer brain metastases. Drug Deliv. Transl. Res..

[15] Cimino-Mathews A., Ye X., Meeker A., Argani P., Emens L.A. (2013). Metastatic triple-negative breast cancers at first relapse have fewer tumor-infiltrating lymphocytes than their matched primary breast tumors: a pilot study. Hum. Pathol..

[16] Sobottka B., Pestalozzi B., Fink D., Moch H., Varga Z. (2016). Similar lymphocytic infiltration pattern in primary breast cancer and their corresponding distant metastases. OncoImmunology.

[17] Ogiya R., Niikura N., Kumaki N., Yasojima H., Iwasa T., Kanbayashi C., Oshitanai R., Tsuneizumi M., Watanabe K.I., Matsui A., Fujisawa T., Saji S., Masuda N., Tokuda Y., Iwata H. (2017). Comparison of immune microenvironments between primary tumors and brain metastases in patients with breast cancer. Oncotarget.

[18] Hainfeld J.F., Ridwan S.M., Stanishevskiy F.Y., Smilowitz H.M. (2020). Iodine nanoparticle radiotherapy of human breast cancer growing in the brains of athymic mice. Sci. Rep..

[19] Verry C., Sancey L., Dufort S., Le Duc G., Mendoza C., Lux F., Grand S., Arnaud J., Quesada J.L., Villa J., Tillement O., Balosso J. (2019). Treatment of multiple brain metastases using gadolinium nanoparticles and radiotherapy: NANO-RAD, a phase I study protocol. BMJ Open.

[20] Chen Y., Jiang T., Zhang H., Gou X., Han C., Wang J., Chen A.T., Ma J., Liu J., Chen Z., Jing X., Lei H., Wang Z., Bao Y., Baqri M., Zhu Y., Bindra R.S., Hansen J.E., Dou J., Huang C., Zhou J. (2020). LRRC31 inhibits DNA repair and sensitizes breast cancer brain metastasis to radiation therapy. Nat. Cell Biol..

[21] Galon J., Bruni D. (2019). Approaches to treat immune hot, altered and cold tumours with combination immunotherapies. Nat. Rev. Drug Discov..

[22] Fitzgerald D.P., Palmieri D., Hua E., Hargrave E., Herring J.M., Qian Y., Vega-Valle E., Weil R.J., Stark A.M., Vortmeyer A.O., Steeg P.S. (2008). Reactive glia are recruited by highly proliferative brain metastases of breast cancer and promote tumor cell colonization. Clin. Exp. Metastasis.

[23] Tomaszewski W., Sanchez-Perez L., Gajewski T.F., Sampson J.H. (2019). Brain tumor microenvironment and host state: implications for immunotherapy. Clin. Cancer Res..

[24] Valiente M., Obenauf A.C., Xin J., Chen Q., Massagué J. (2014). Serpins promote cancer cell survival and vascular Co-option in brain metastasis. Cell.

[25] Ridler C. (2018). New drug blocks brain metastasis. Nat. Rev. Neurol..

[26] Zhao Z., Zhang Y., Li C., Li X., Chu Y., Guo Q., Zhang Y., Xia W., Liu P., Chen H., Wang Y., Li C., Sun T., Jiang C. (2022). Microenvironment-tailored micelles restrain carcinoma-astrocyte crosstalk for brain metastasis. J. Contr. Release.

[27] Chen Q., Liu L., Lu Y., Chen X., Zhang Y., Zhou W., Guo Q., Li C., Zhang Y., Zhang Y., Liang D., Sun T., Jiang C. (2019). Tumor microenvironment-triggered aggregated magnetic nanoparticles for reinforced image-guided immunogenic chemotherapy. Adv. Sci..

[28] Alkilany A.M., Zhu L., Weller H., Mews A., Parak W.J., Barz M., Feliu N. (2019). Ligand density on nanoparticles: a parameter with critical impact on nanomedicine. Adv. Drug Deliv. Rev..

[29] Santaguida S., Janigro D., Hossain M., Oby E., Rapp E., Cucullo L. (2006). Side by side comparison between dynamic versus static models of blood-brain barrier in vitro: a permeability study. Brain Res..

[30] Patel T., Zhou J., Piepmeier J.M., Saltzman W.M. (2012). Polymeric nanoparticles for drug delivery to the central nervous system. Adv. Drug Deliv. Rev..

[31] Bradbury M.W., Westrop R.J. (1983). Factors influencing exit of substances from cerebrospinal fluid into deep cervical lymph of the rabbit. J. Physiol..

[32] Cserr H.F., Knopf P.M. (1992). Cervical lymphatics, the blood-brain barrier and the immunoreactivity of the brain: a new view. Immunol. Today.

[33] Goldmann J., Kwidzinski E., Brandt C., Mahlo J., Richter D., Bechmann I. (2006). T cells traffic from brain to cervical lymph nodes via the cribroid plate and the nasal mucosa. J. Leukoc. Biol..

[34] Widner H., Jönsson B.A., Hallstadius L., Wingårdh K., Strand S.E., Johansson B.B. (1987). Scintigraphic method to quantify the passage from brain parenchyma to the deep cervical lymph nodes in rats. Eur. J. Nucl. Med..

[35] Eide P.K., Vatnehol S.A.S., Emblem K.E., Ringstad G. (2018). Magnetic resonance imaging provides evidence of glymphatic drainage from human brain to cervical lymph nodes. Sci. Rep..

[36] Iliff J.J., Wang M., Liao Y., Plogg B.A., Peng W., Gundersen G.A., Benveniste H., Vates G.E., Deane R., Goldman S.A., Nagelhus E.A., Nedergaard M. (2012). A paravascular pathway facilitates CSF flow through the brain parenchyma and the clearance of interstitial solutes, including amyloid β. Sci. Transl. Med..

[37] Aspelund A., Antila S., Proulx S.T., Karlsen T.V., Karaman S., Detmar M., Wiig H., Alitalo K. (2015). A dural lymphatic vascular system that drains brain interstitial fluid and macromolecules. J. Exp. Med..

[38] Louveau A., Herz J., Alme M.N., Salvador A.F., Dong M.Q., Viar K.E., Herod S.G., Knopp J., Setliff J.C., Lupi A.L., Da Mesquita S., Frost E.L., Gaultier A., Harris T.H., Cao R., Hu S., Lukens J.R., Smirnov I., Overall C.C., Oliver G., Kipnis J. (2018). CNS lymphatic drainage and neuroinflammation are regulated by meningeal lymphatic vasculature. Nat. Neurosci..

[39] Louveau A., Smirnov I., Keyes T.J., Eccles J.D., Rouhani S.J., Peske J.D., Derecki N.C., Castle D., Mandell J.W., Lee K.S., Harris T.H., Kipnis J. (2015). Structural and functional features of central nervous system lymphatic vessels. Nature.

[40] Sampson J.H., Gunn M.D., Fecci P.E., Ashley D.M. (2020). Brain immunology and immunotherapy in brain tumours. Nat. Rev. Cancer.

[41] Schläger C., Körner H., Krueger M., Vidoli S., Haberl M., Mielke D., Brylla E., Issekutz T., Cabañas C., Nelson P.J., Ziemssen T., Rohde V., Bechmann I., Lodygin D., Odoardi F., Flügel A. (2016). Effector T-cell trafficking between the leptomeninges and the cerebrospinal fluid. Nature.

[42] Zingoni A., Fionda C., Borrelli C., Cippitelli M., Santoni A., Soriani A. (2017). Natural killer cell response to chemotherapy-stressed cancer cells: role in tumor immunosurveillance. Front. Immunol..

[43] Wu J., Waxman D.J. (2015). Metronomic cyclophosphamide eradicates large implanted GL261 gliomas by activating antitumor Cd8(+) T-cell responses and immune memory. OncoImmunology.

[44] Lotze M.T., Tracey K.J. (2005). High-mobility group box 1 protein (HMGB1): nuclear weapon in the immune arsenal. Nat. Rev. Immunol..

[45] Fecci P.E., Heimberger A.B., Sampson J.H. (2014). Immunotherapy for primary brain tumors: no longer a matter of privilege. Clin. Cancer Res..

[46] Pedrosa R., Mustafa D.A., Soffietti R., Kros J.M. (2018). Breast cancer brain metastasis: molecular mechanisms and directions for treatment. Neuro Oncol..

[47] Kong L.Y., Abou-Ghazal M.K., Wei J., Chakraborty A., Sun W., Qiao W., Fuller G.N., Fokt I., Grimm E.A., Schmittling R.J., Archer G.E., Sampson J.H., Priebe W., Heimberger A.B. (2008). A novel inhibitor of signal transducers and activators of transcription 3 activation is efficacious against established central nervous system melanoma and inhibits regulatory T cells. Clin. Cancer Res..

[48] Priego N., Zhu L., Monteiro C., Mulders M., Wasilewski D., Bindeman W., Doglio L., Martínez L., Martínez-Saez E., Ramón Y.C.S., Megías D., Hernández-Encinas E., Blanco-Aparicio C., Martínez L., Zarzuela E., Muñoz J., Fustero-Torre C., Piñeiro-Yáñez E., Hernández-Laín A., Bertero L., Poli V., Sanchez-Martinez M., Menendez J.A., Soffietti R., Bosch-Barrera J., Valiente M. (2018). STAT3 labels a subpopulation of reactive astrocytes required for brain metastasis. Nat. Med..

[49] Schulz M., Salamero-Boix A., Niesel K., Alekseeva T., Sevenich L. (2019). Microenvironmental regulation of tumor progression and therapeutic response in brain metastasis. Front. Immunol..

[50] Henrik Heiland D., Ravi V.M., Behringer S.P., Frenking J.H., Wurm J., Joseph K., Garrelfs N.W.C., Strähle J., Heynckes S., Grauvogel J., Franco P., Mader I., Schneider M., Potthoff A.L., Delev D., Hofmann U.G., Fung C., Beck J., Sankowski R., Prinz M., Schnell O. (2019). Tumor-associated reactive astrocytes aid the evolution of immunosuppressive environment in glioblastoma. Nat. Commun..

[51] Nduom E.K., Weller M., Heimberger A.B. (2015). Immunosuppressive mechanisms in glioblastoma. Neuro Oncol..

[52] Valiente M., Van Swearingen A.E.D., Anders C.K., Bairoch A., Boire A., Bos P.D., Cittelly D.M., Erez N., Ferraro G.B., Fukumura D., Gril B., Herlyn M., Holmen S.L., Jain R.K., Joyce J.A., Lorger M., Massague J., Neman J., Sibson N.R., Steeg P.S., Thorsen F., Young L.S., Varešlija D., Vultur A., Weis-Garcia F., Winkler F. (2020). Brain metastasis cell lines panel: a public resource of organotropic cell lines. Cancer Res..

[53] Balathasan L., Beech J.S., Muschel R.J. (2013). Ultrasonography-guided intracardiac injection: an improvement for quantitative brain colonization assays. Am. J. Pathol..

[54] Bos P.D., Zhang X.H., Nadal C., Shu W., Gomis R.R., Nguyen D.X., Minn A.J., van de Vijver M.J., Gerald W.L., Foekens J.A., Massagué J. (2009). Genes that mediate breast cancer metastasis to the brain. Nature.

[55] Nguyen D.X., Chiang A.C., Zhang X.H., Kim J.Y., Kris M.G., Ladanyi M., Gerald W.L., Massagué J. (2009). WNT/TCF signaling through LEF1 and HOXB9 mediates lung adenocarcinoma metastasis. Cell.

[56] Taggart D., Andreou T., Scott K.J., Williams J., Rippaus N., Brownlie R.J., Ilett E.J., Salmond R.J., Melcher A., Lorger M. (2018). Anti-PD-1/anti-CTLA-4 efficacy in melanoma brain metastases depends on extracranial disease and augmentation of CD8(+) T cell trafficking. Proc. Natl. Acad. Sci. USA.

