# Supplementary material for: Nanomaterials with dual immunomodulatory functions for synergistic therapy of breast cancer brain metastases
Source: Bioact Mater. 2023 Apr 27;27:474–87. doi: 10.1016/j.bioactmat.2023.04.021 (PMC10163467; doi:10.1016/j.bioactmat.2023.04.021)
Supplement: Multimedia component 1 [file mmc1.docx]

Supporting Information

Nanomaterials with dual immunomodulatory functions for synergistic therapy of breast cancer brain metastases

Zhenhao Zhao, Chufeng Li, Yiwen Zhang, Chao Li, Yongchao Chu, Xuwen Li, Peixin Liu, Hongyi Chen, Yu Wang, Boyu Su, Qinjun Chen, Tao Sun and Chen Jiang*

Department of Pharmaceutics, School of Pharmacy, Fudan University; Key Laboratory of Smart Drug Delivery, Ministry of Education; State Key Laboratory of Medical Neurobiology and MOE Frontiers Center for Brain Science, Shanghai 201203, China

* Corresponding author

Chen Jiang, Email: [jiangchen@shmu.edu.cn](mailto:jiangchen@shmu.edu.cn)

**This Word file includes:**

Supplementary Text

Figs. S1 to S34

Tables S1 to S4

Experimental Section/Methods


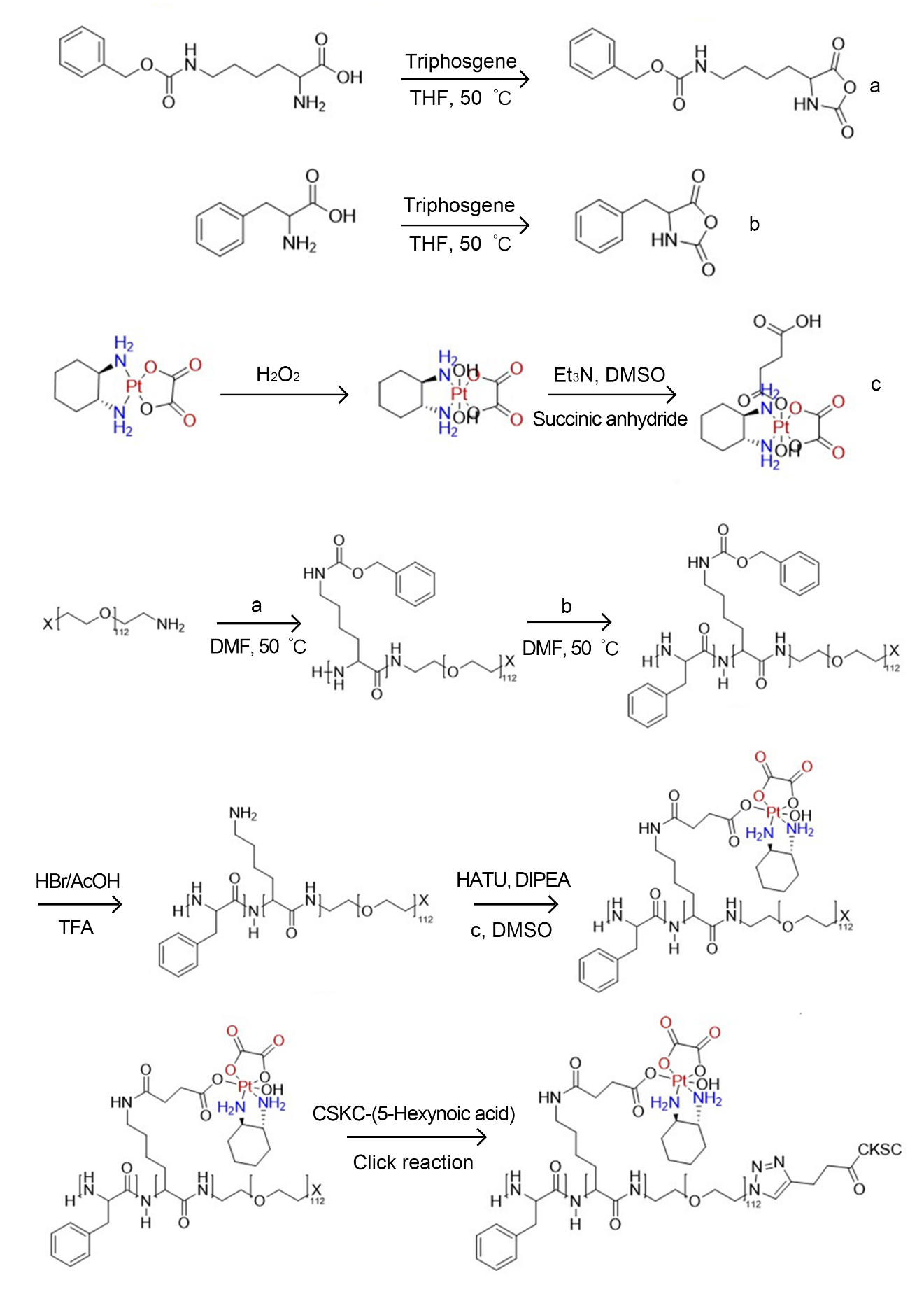


**Fig. S1.** Synthetic route of CSKC-PEG-*p*Lys/OXA-*p*Phe.


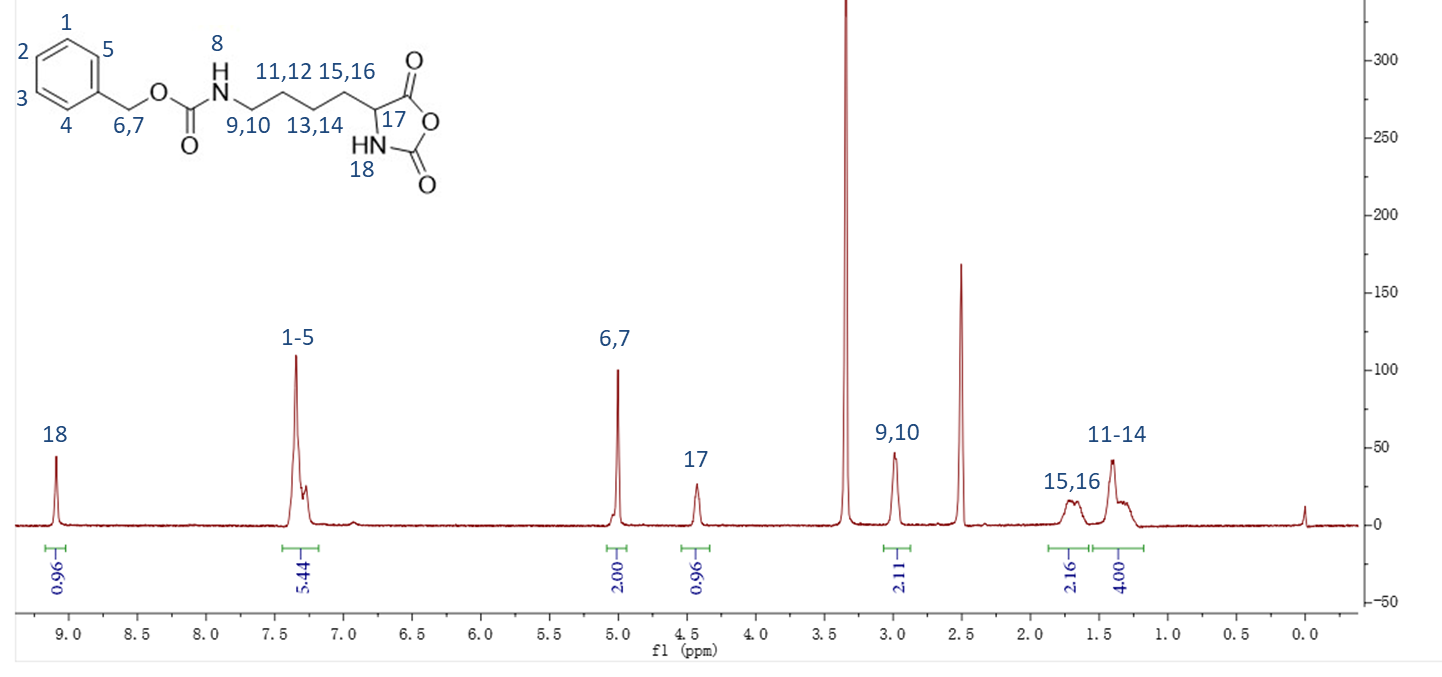


**Fig. S2.** ^1^H NMR (400 MHz, DMSO-*d_6_*) spectrum of Lys(*Z*)-NCA.

^1^H NMR (400 MHz, DMSO-*d*_6_) δ: 9.09 (s, 1H), 7.45-7.18 (m, 5H), 5.00 (s, 2H), 4.43 (s, 1H), 2.98 (d, *J* = 6.6 Hz, 2H), 1.69 (d, *J* = 27.1 Hz, 2H), 1.55-1.18 (m, 4H).


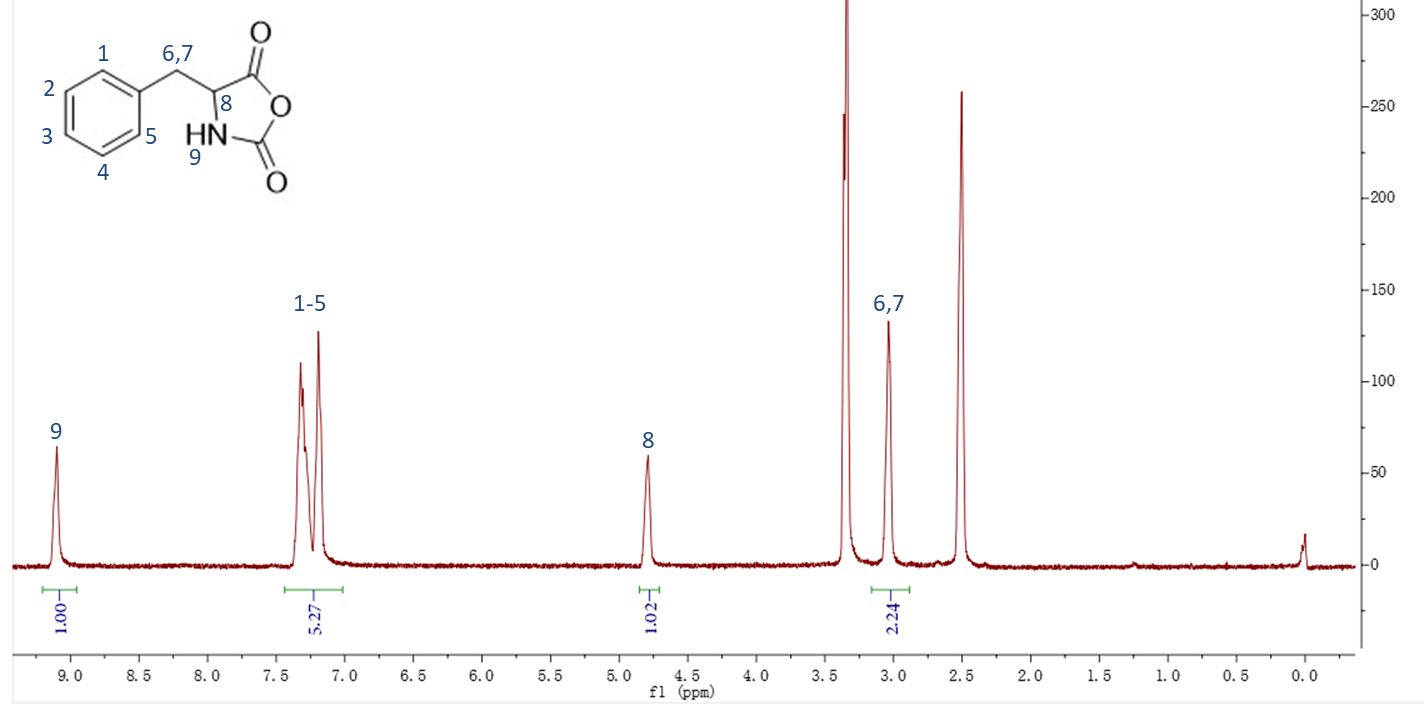


**Fig. S3.** ^1^H NMR (400 MHz, DMSO-*d_6_*) spectrum of Phe-NCA.

^1^H NMR (400 MHz, DMSO-*d*_6_) δ: 9.10 (s, 1H), 7.44-7.02 (m, 5H), 4.79 (t, *J* = 5.4 Hz, 1H), 3.04 (dd, *J* = 8.7, 4.9 Hz, 2H).


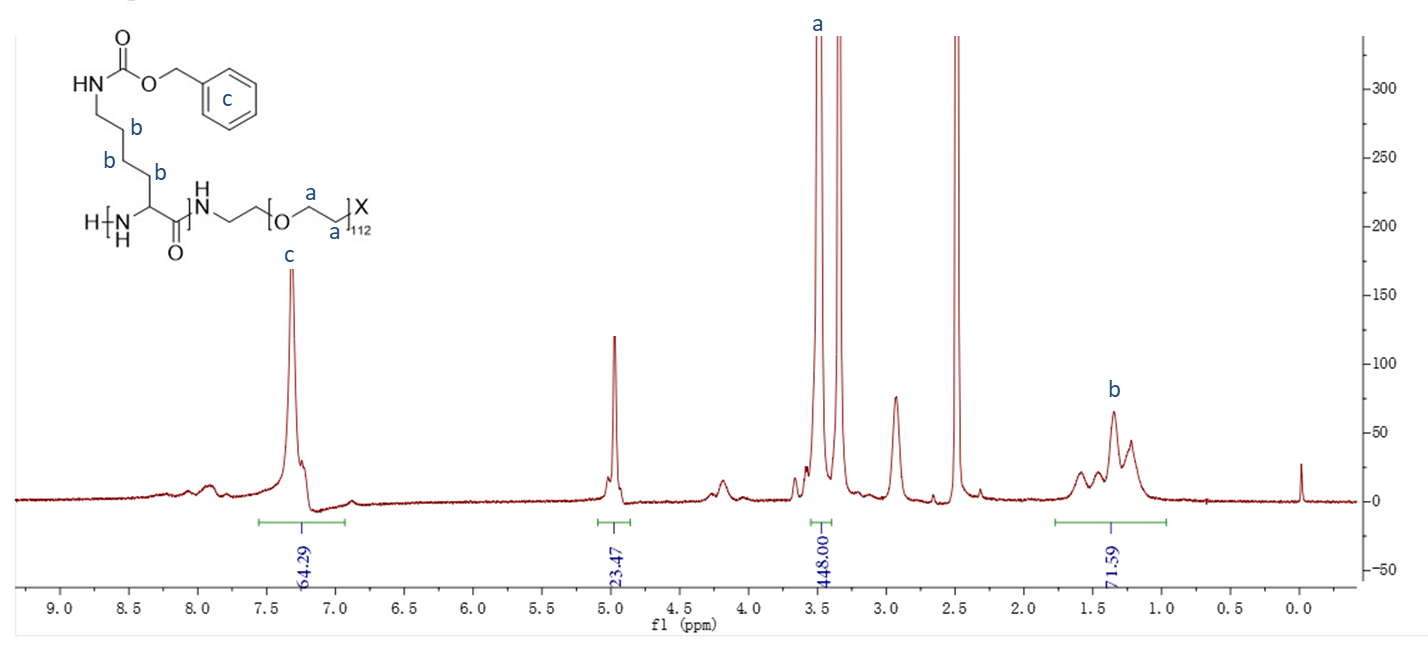


**Fig. S4.** ^1^H NMR (400 MHz, DMSO-*d_6_*) spectrum of PEG-*p*Lys(*Z*).

^1^H NMR (400 MHz, DMSO-*d*_6_) δ: 7.32 (s, 64H), 4.97 (s, 23H), 3.48 (s, 448H), 1.40 (q, *J* = 50.1, 49.1 Hz, 72H).


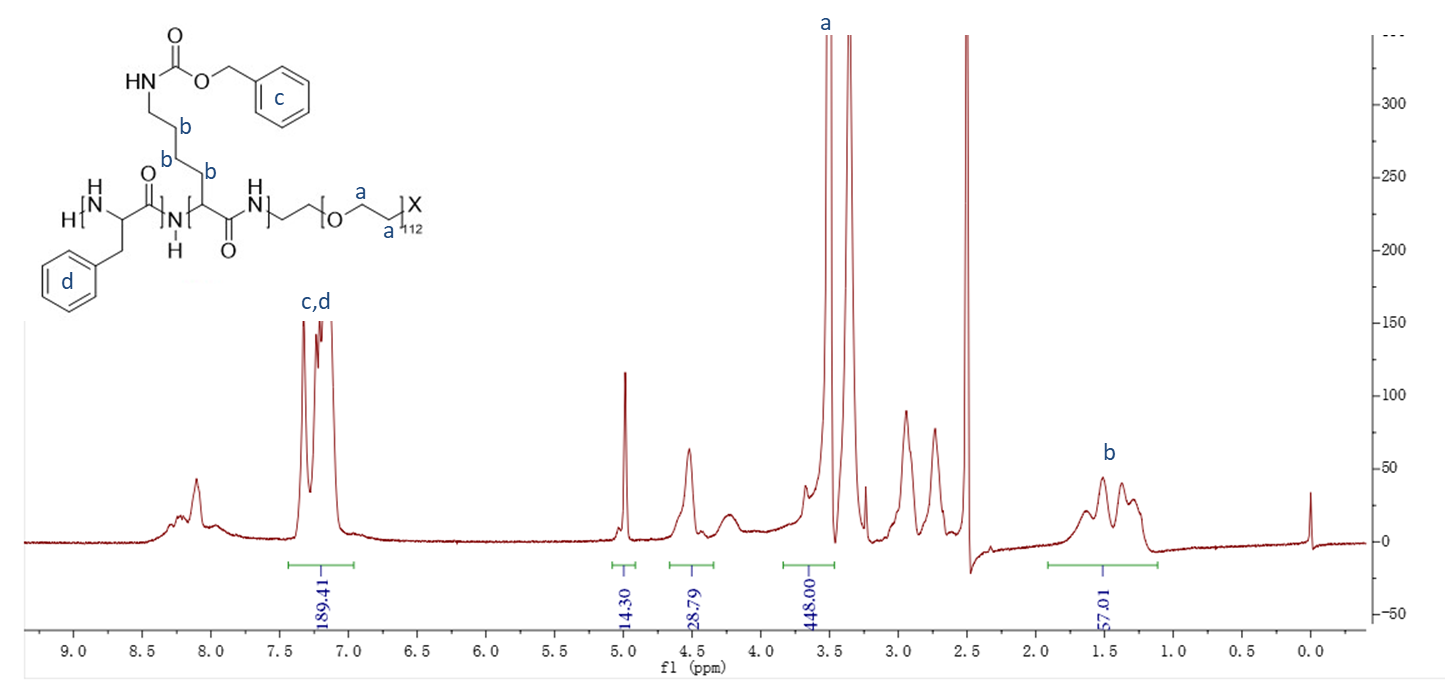


**Fig. S5.** ^1^H NMR (400 MHz, DMSO-*d_6_*) spectrum of PEG-*p*Lys(*Z*)-*p*Phe.

^1^H NMR (400 MHz, DMSO-*d*_6_) δ: 7.44-6.96 (m, 189H), 4.99 (s, 14H), 4.52 (s, 29H), 3.52 (s, 448H), 1.91-1.11 (m, 57H).


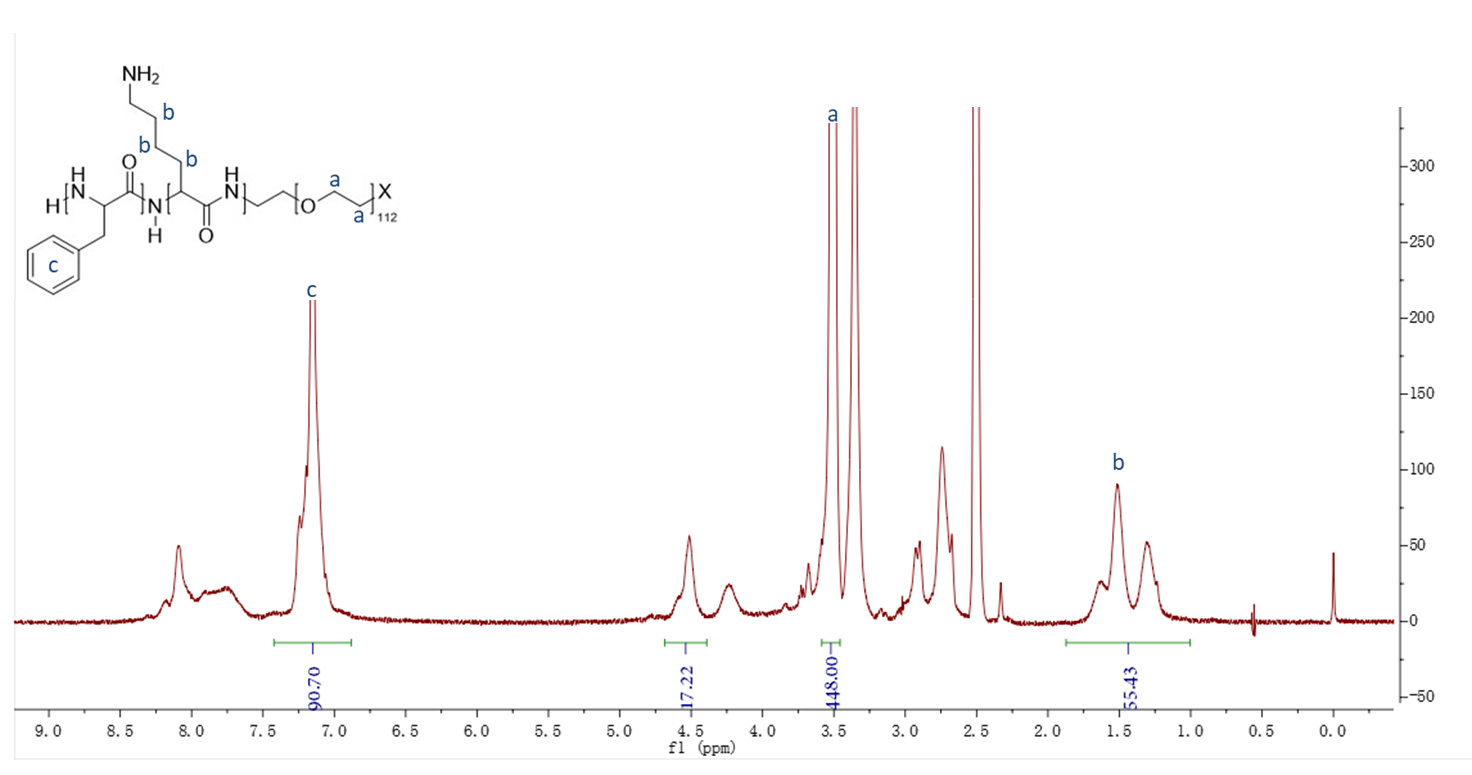


**Fig. S6.** ^1^H NMR (400 MHz, DMSO-*d_6_*) spectrum of PEG-*p*Lys-*p*Phe.

^1^H NMR (400 MHz, DMSO-*d*_6_) δ: 7.15 (s, 91H), 4.51 (s, 17H), 3.51 (s, 448H), 1.41 (d, *J* = 83.8 Hz, 55H).


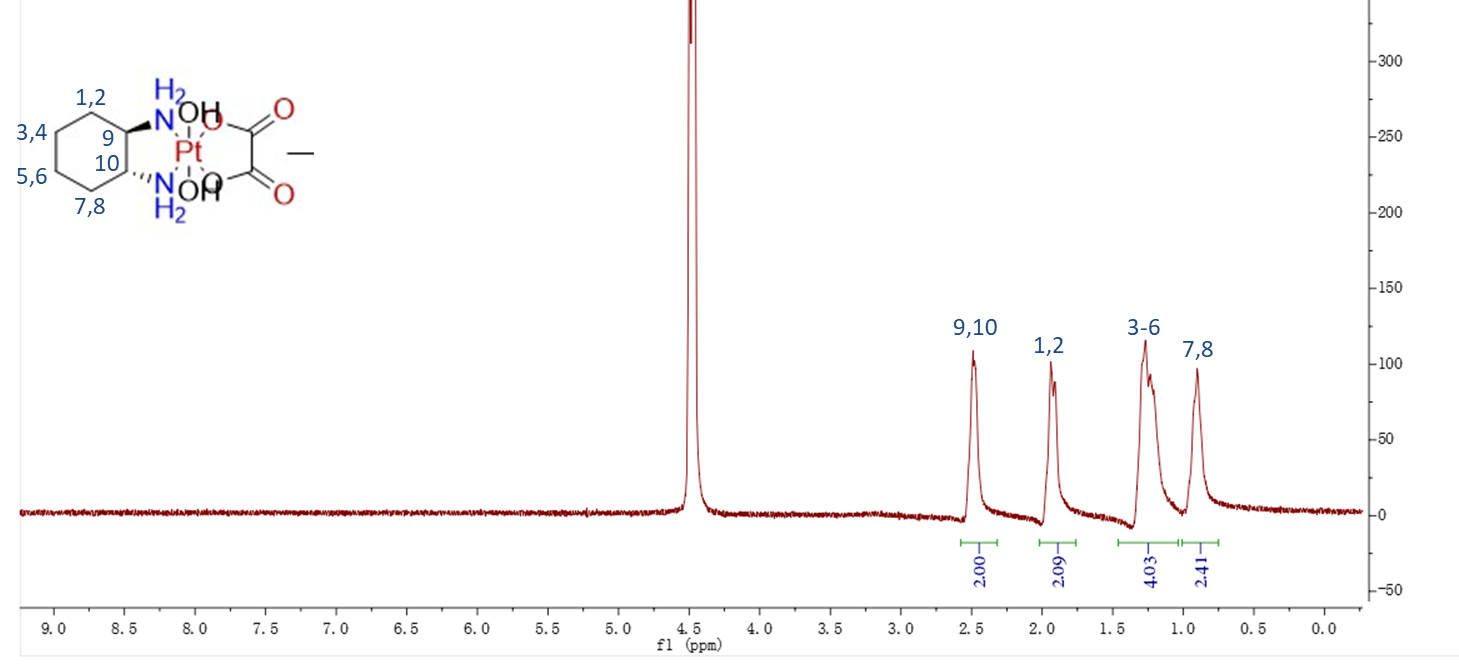


**Fig. S7.** ^1^H NMR (400 MHz, D_2_O-*d_2_*) spectrum of OXA-OH.

^1^H NMR (400 MHz, D_2_O-*d_2_*) δ: 2.48 (s, 2H), 2.02-1.76 (m, 2H), 1.26 (d, *J* = 16.9 Hz, 4H), 0.90 (s, 2H).


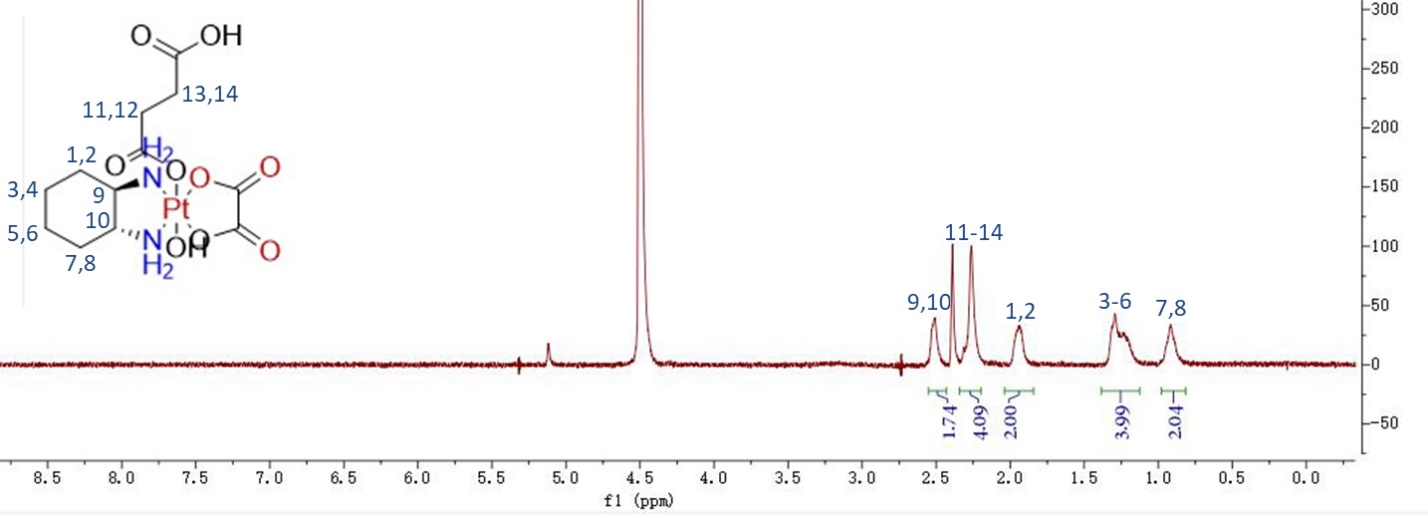


**Fig. S8.** ^1^H NMR (400 MHz, D_2_O-*d_2_*) spectrum of OXA-COOH.

^1^H NMR (400 MHz, D_2_O-*d_2_*) δ: 2.51 (s, 2H), 2.26 (s, 4H), 1.89 (s, 2H), 1.26 (d, *J* = 28.1 Hz, 4H), 0.91 (s, 2H).


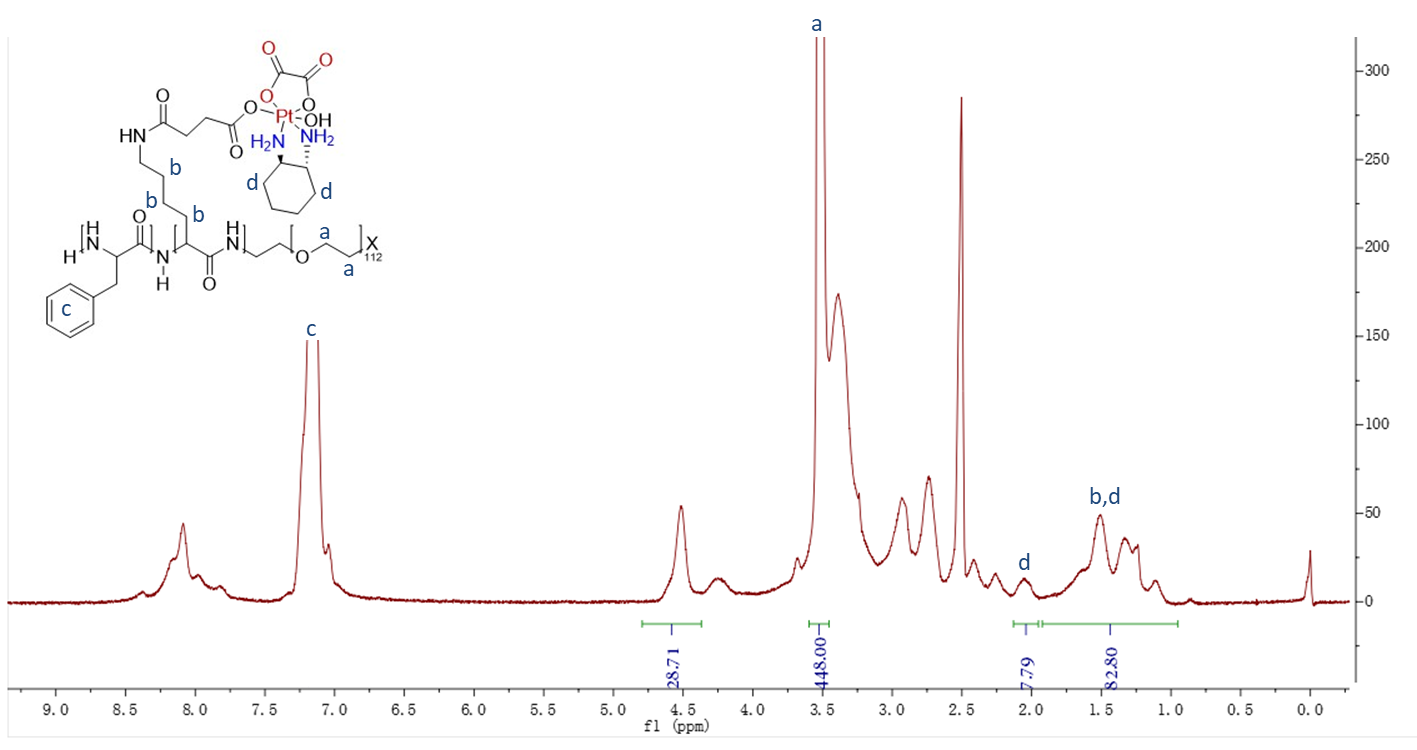


**Fig. S9.** ^1^H NMR (400 MHz, DMSO-*d_6_*) spectrum of PEG-*p*Lys/OXA-*p*Phe.

^1^H NMR (400 MHz, DMSO-*d*_6_) δ: 7.16 (s, 162H), 4.52 (s, 29H), 3. 51 (m, 448H), 2.05 (s, 8H), 1.30 (dd, *J* = 98.4, 62.3 Hz, 83H).


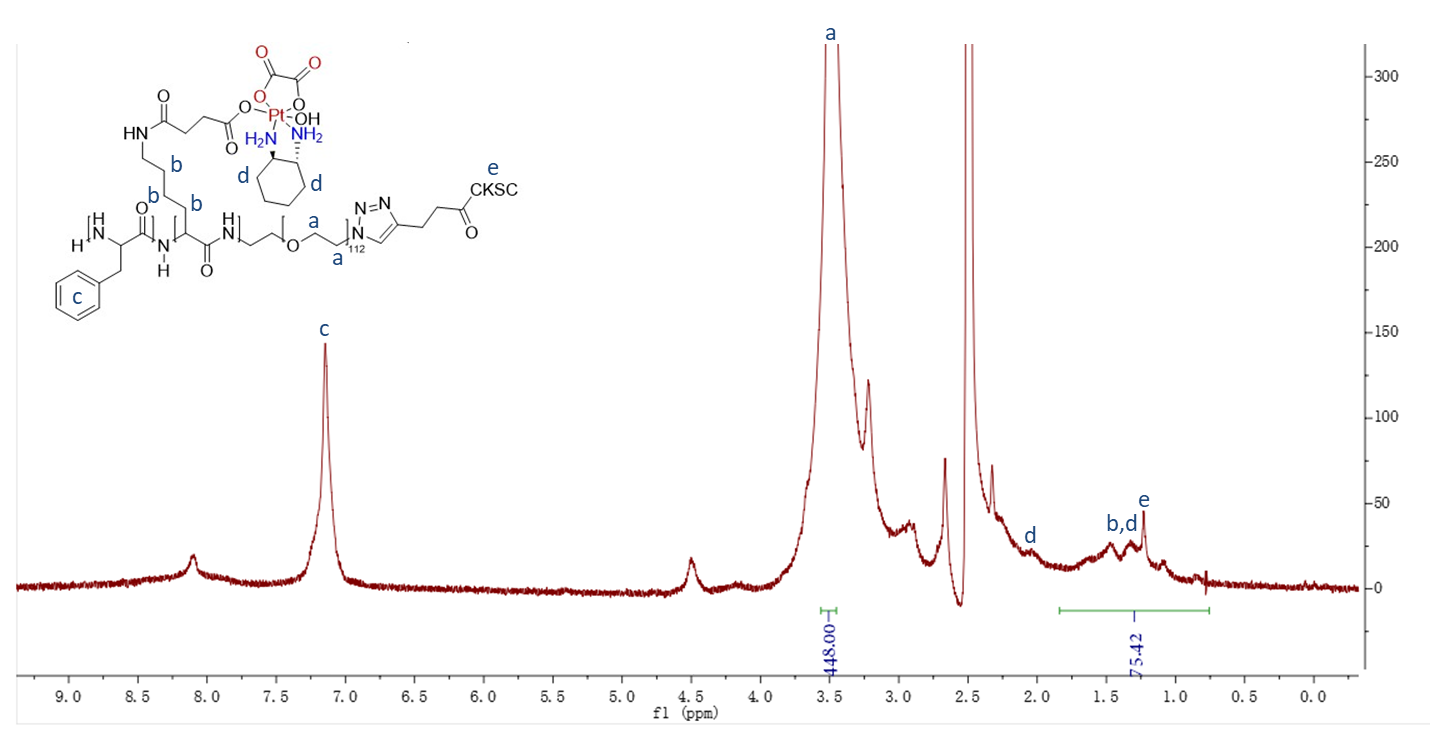


**Fig. S10.** ^1^H NMR (400 MHz, DMSO-*d_6_*) spectrum of CSKC-PEG-*p*Lys/OXA-*p*Phe.

^1^H NMR (400 MHz, DMSO-*d*_6_) δ: 3. 51 (m, 448H), 1.28 (q, *J* = 58.1, 55.4 Hz, 75H).


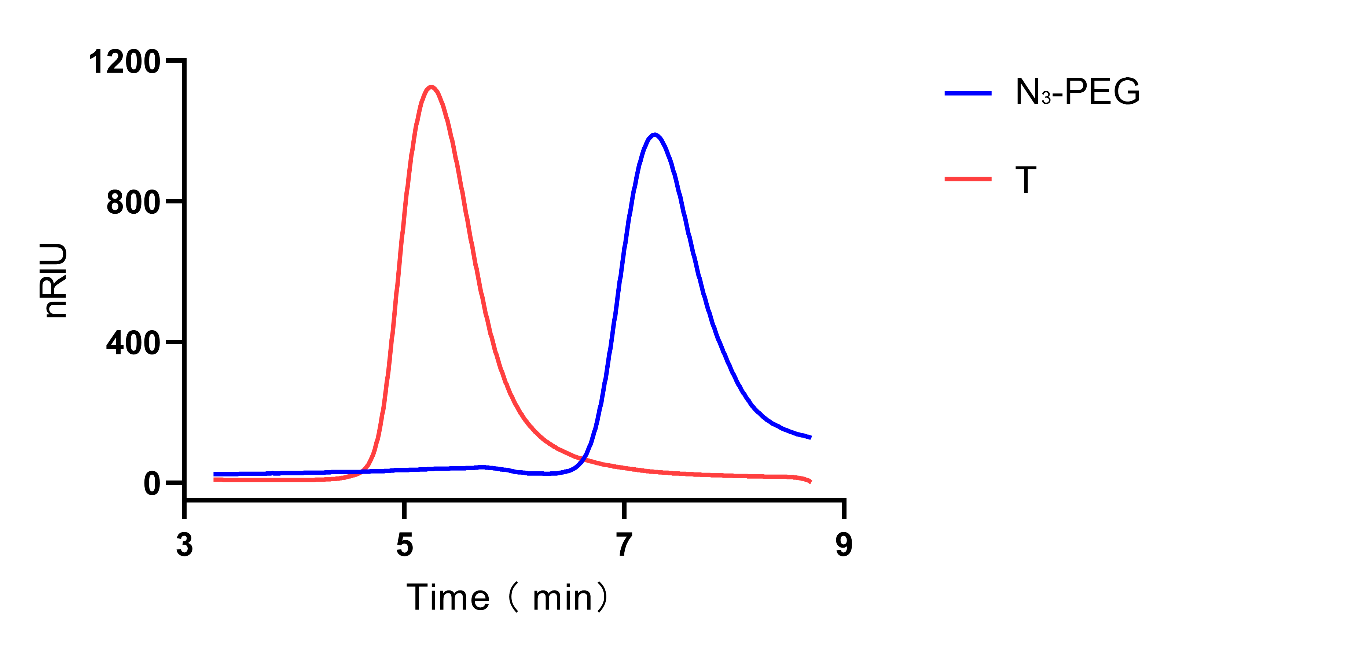


**Fig. S11.** GPC analysis of CSKC-PEG-*p*Lys/OXA-*p*Phe.


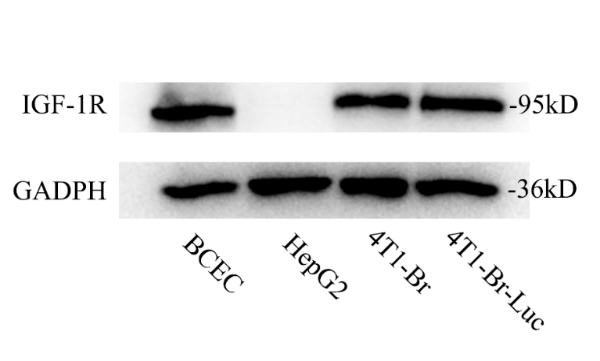


**Fig. S12.** The expression of IGF-1R on different cells.


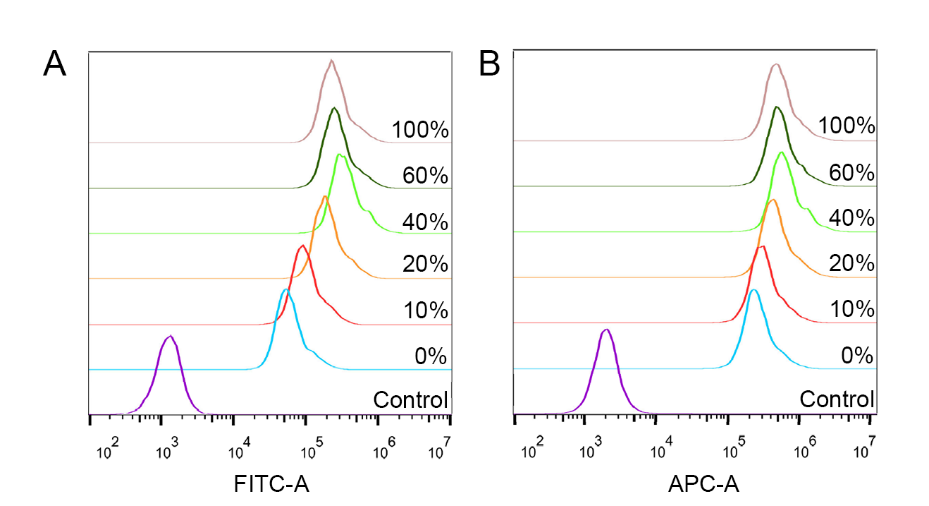


**Fig. S13.** Cellular uptake of double fluorescently labeled micelles with different degrees of CSKC peptide modification on 4T1-Br-Luc cells for 1 hour. (A) FITC-A: coumarin-6; (B) APC-A: micelles.


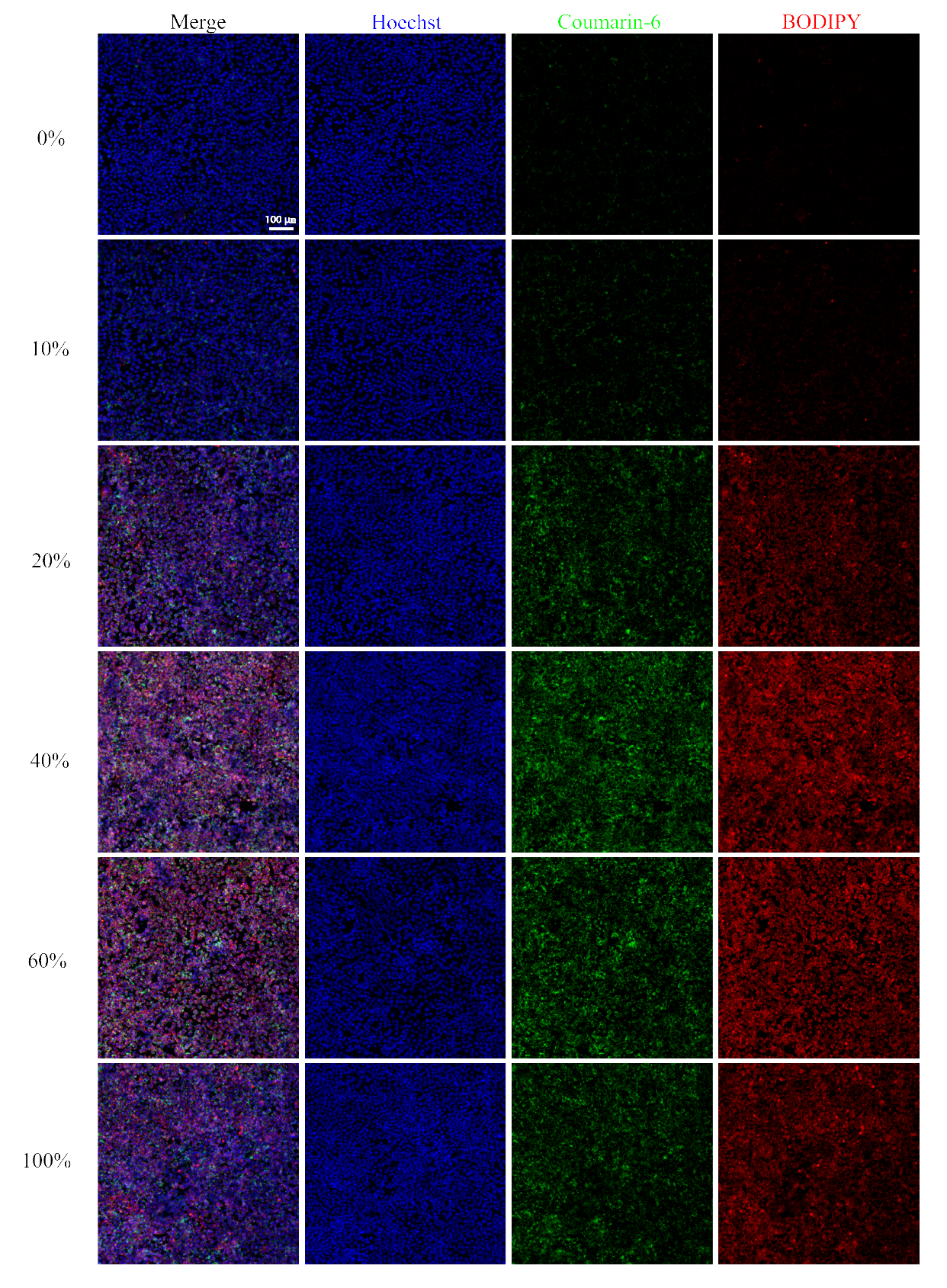


**Fig. S14.** Fluorescence imaging of cellular uptake of dual fluorescently labeled micelles with different degrees of CSKC modification on 4T1-Br-Luc cells for 1 h. Blue: nucleus; green: coumarin-6; red: micelles. Scale bar: 100 μm.


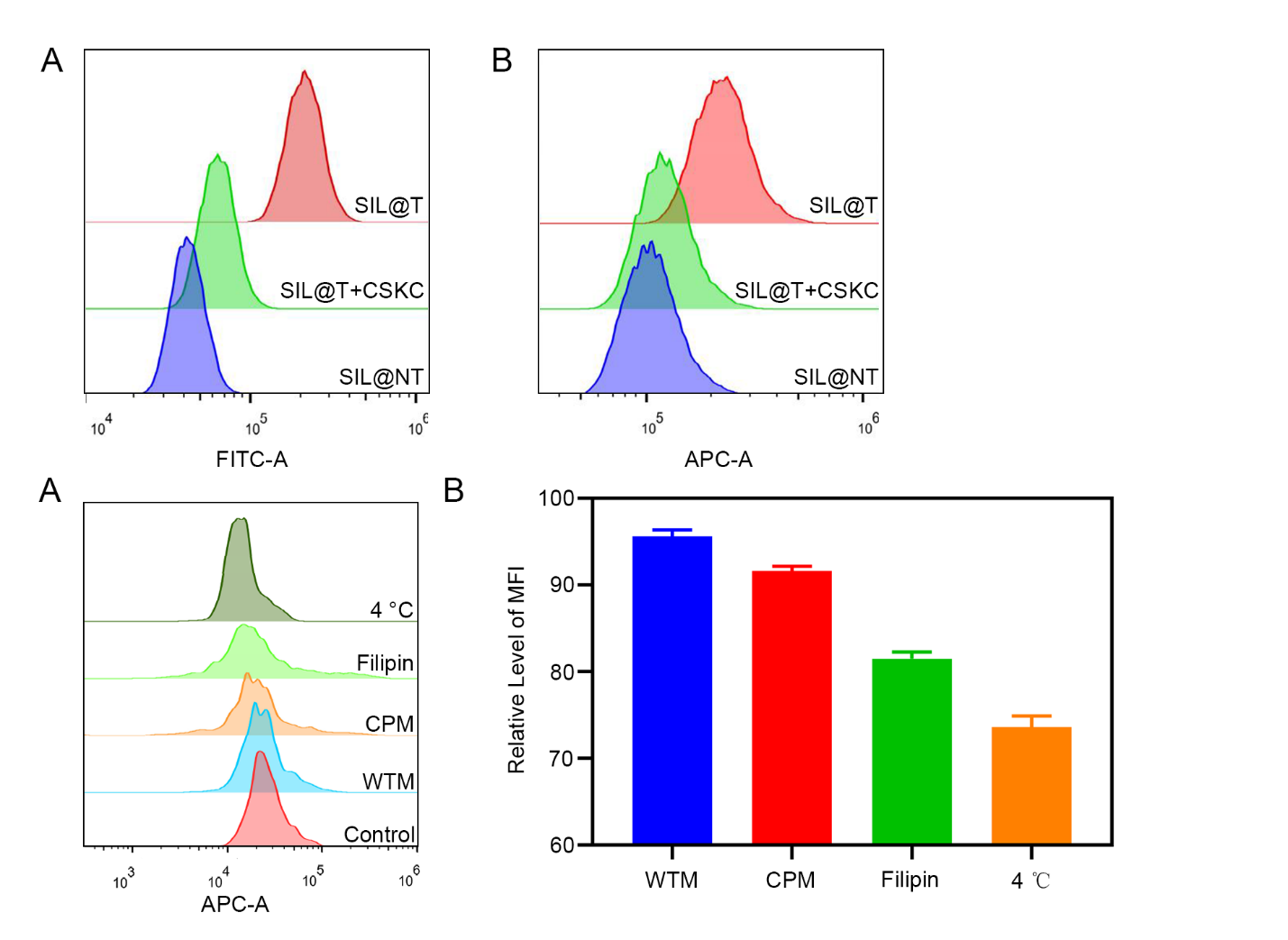


**Fig. S15.** Flow cytometry results of micellar uptake by 4T1-Br-Luc cells (1 h after formulation administration). (A) FITC-A: coumarin-6; (B) APC-A: micelles.

**
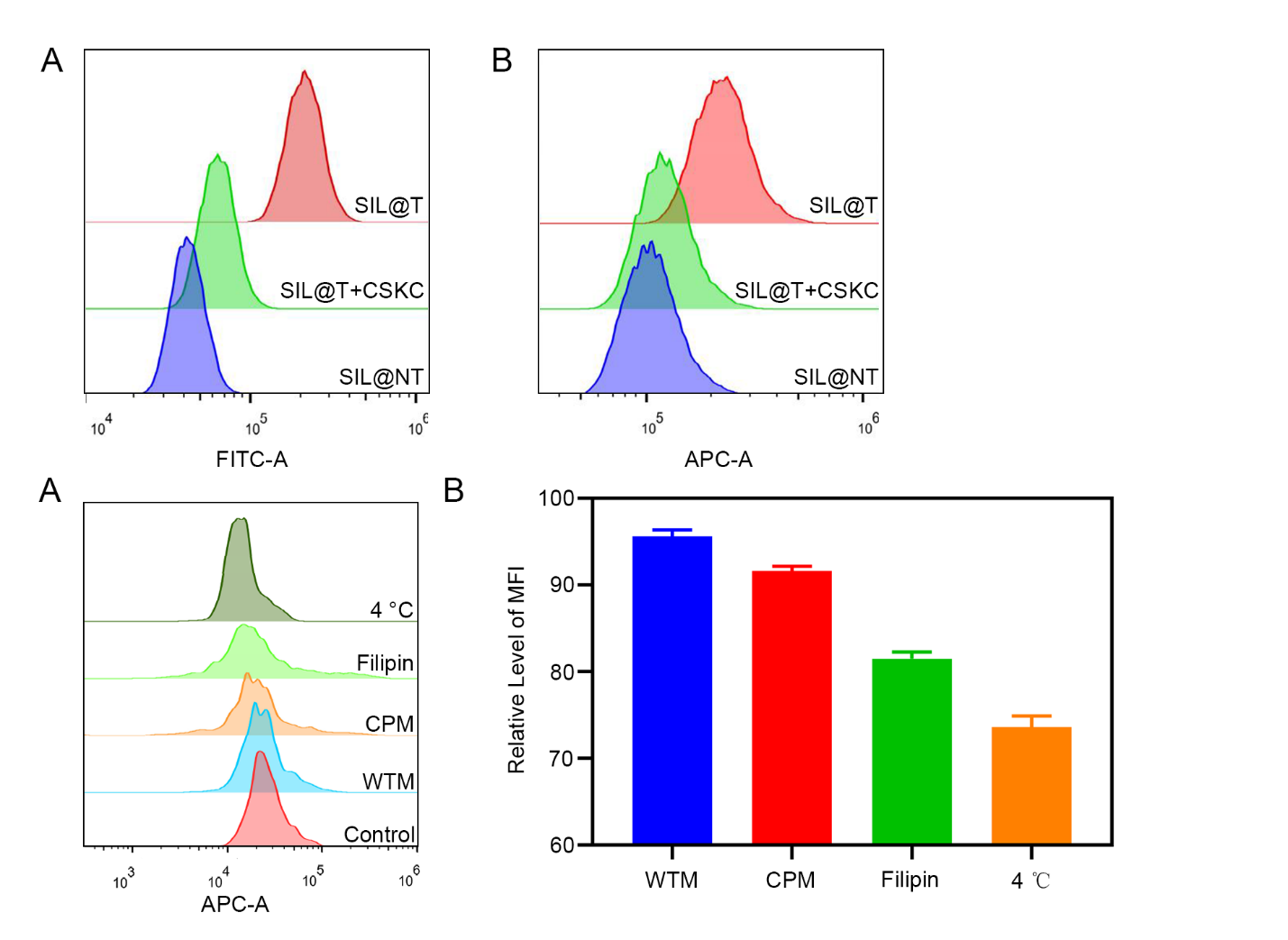
**

**Fig. S16.** Flow cytometry results of SIL@T uptake by 4T1-Br-Luc cells after pretreatment with inhibitors of different uptake pathways (1 h after formulation administration). (A) APC-A: micelles; (B) Quantitative results of panel A.


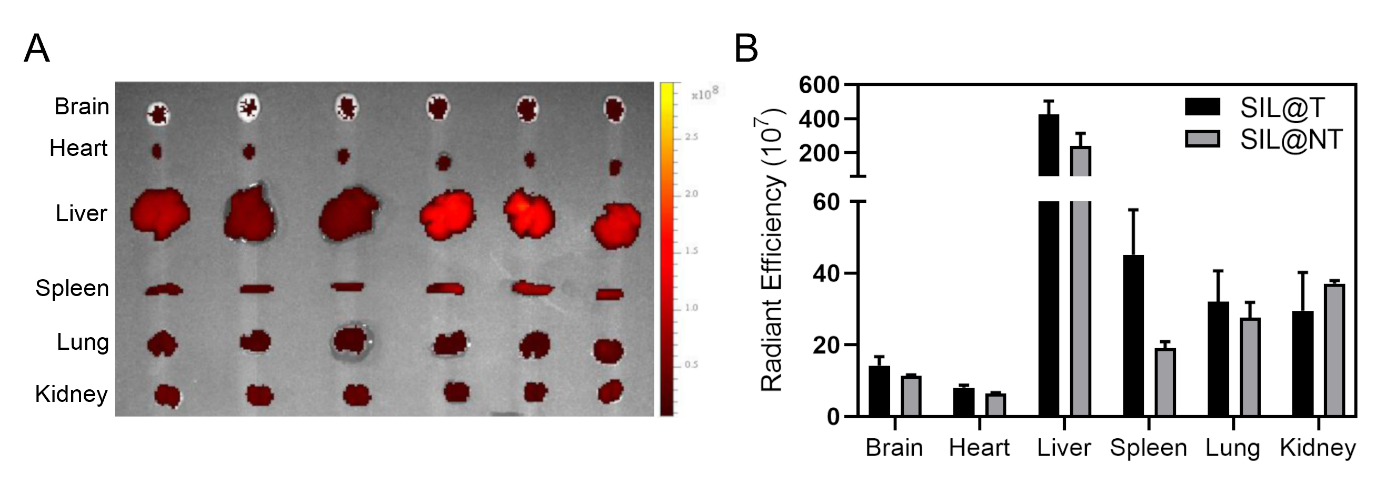


**Fig. S17.** (A) IVIS imaging of tissue distribution after administration of BODIPY-labeled micelles for 24 h (three left groups: SIL@NT; three right groups: SIL@T). (B) Tissue distribution of micelles 24 h after tail vein injection of BODIPY-labeled micelles (n=3).


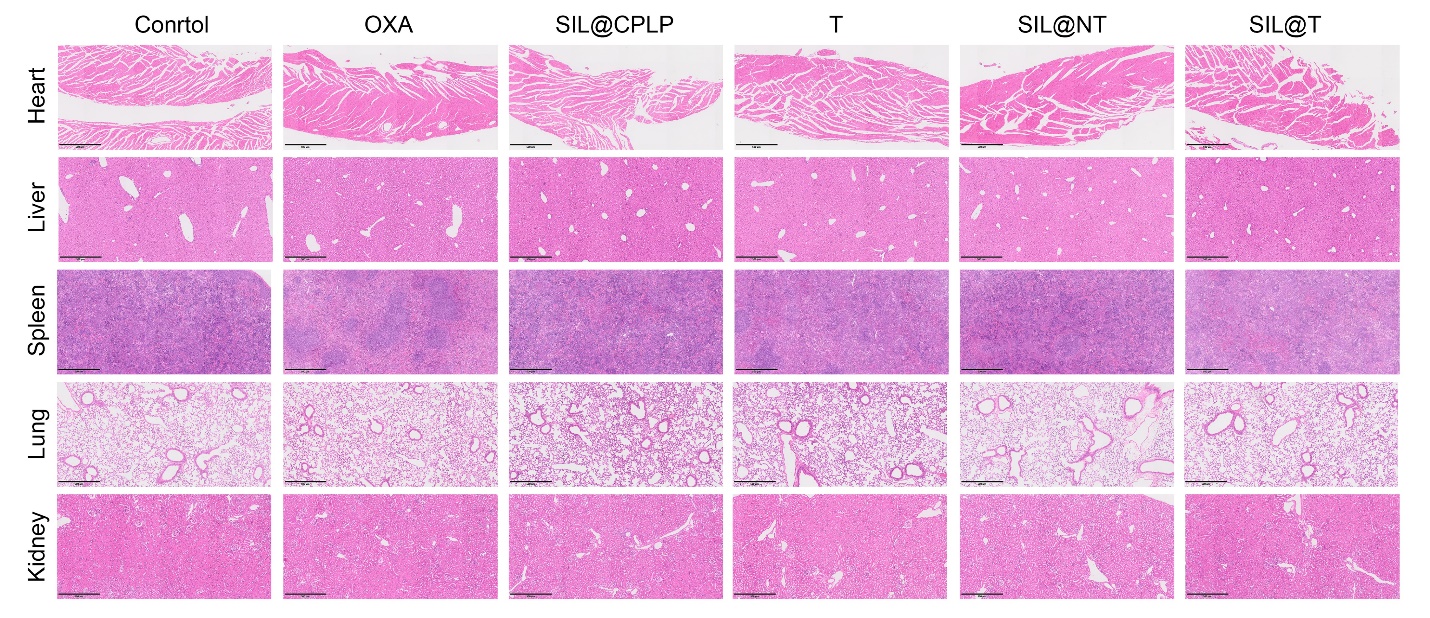


**Fig. S18.** HE staining of main organs.


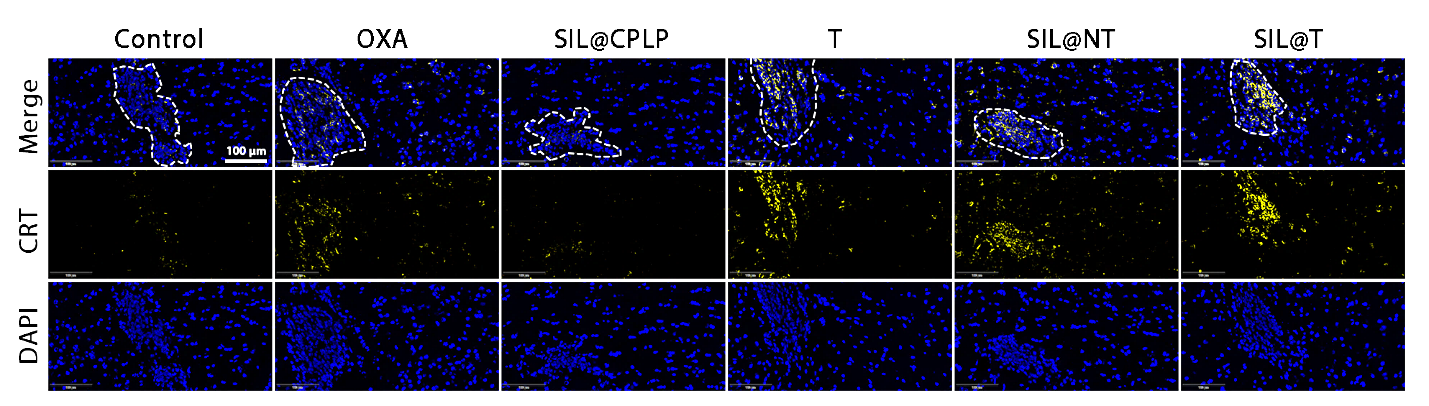


**Fig. S19.** Immunofluorescence imaging of CRT in brain cryosections of BM-mice. Blue: nucleus; yellow: CRT. Scale bar: 100 μm.


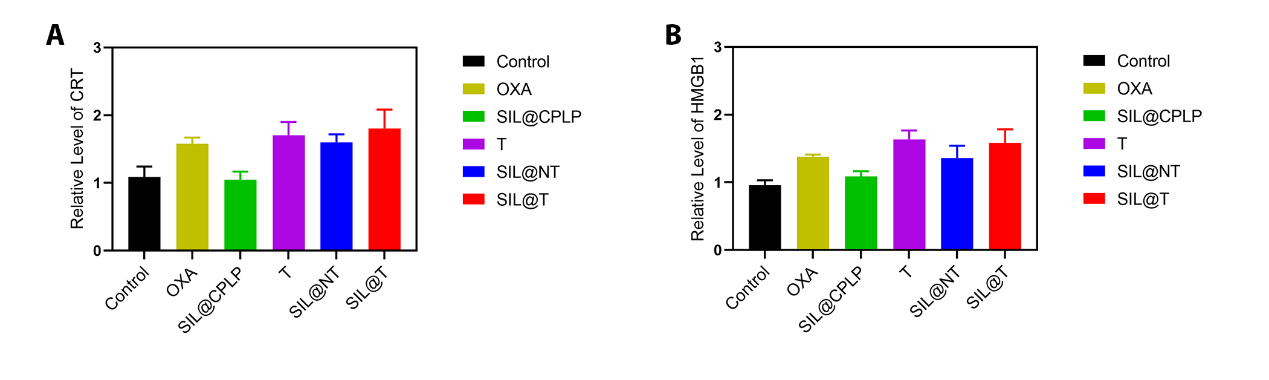


**Fig. S20.** WB quantification results of CRT (A) and HMGB1 (B) in Figure 5G.


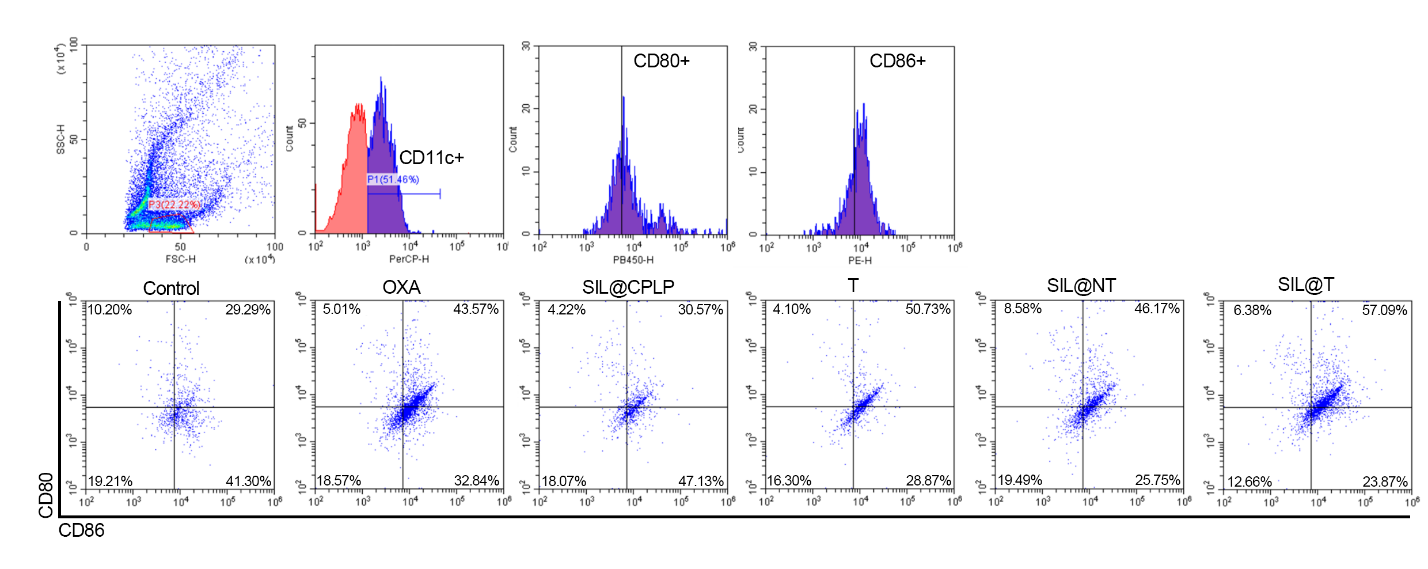


**Fig. S21.** Flow cytometry analysis of DC maturation in the cervical lymph node of BM-mice.


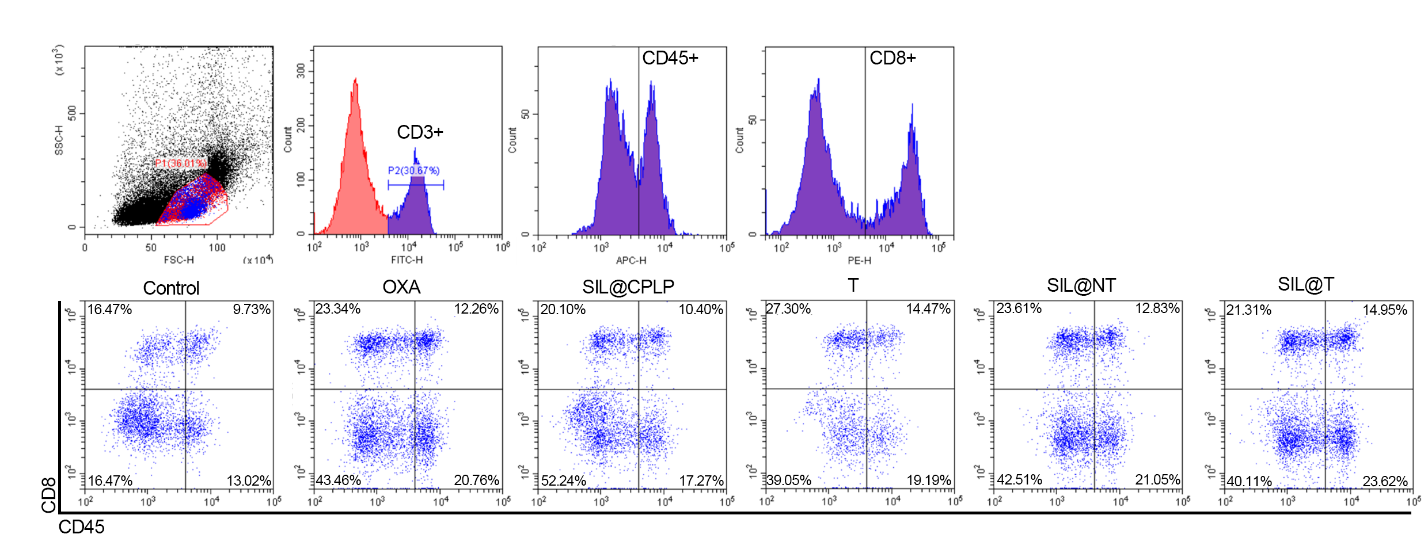


**Fig. S22.** Flow cytometry analysis of T cell polarization in the spleen of BM-mice.

**
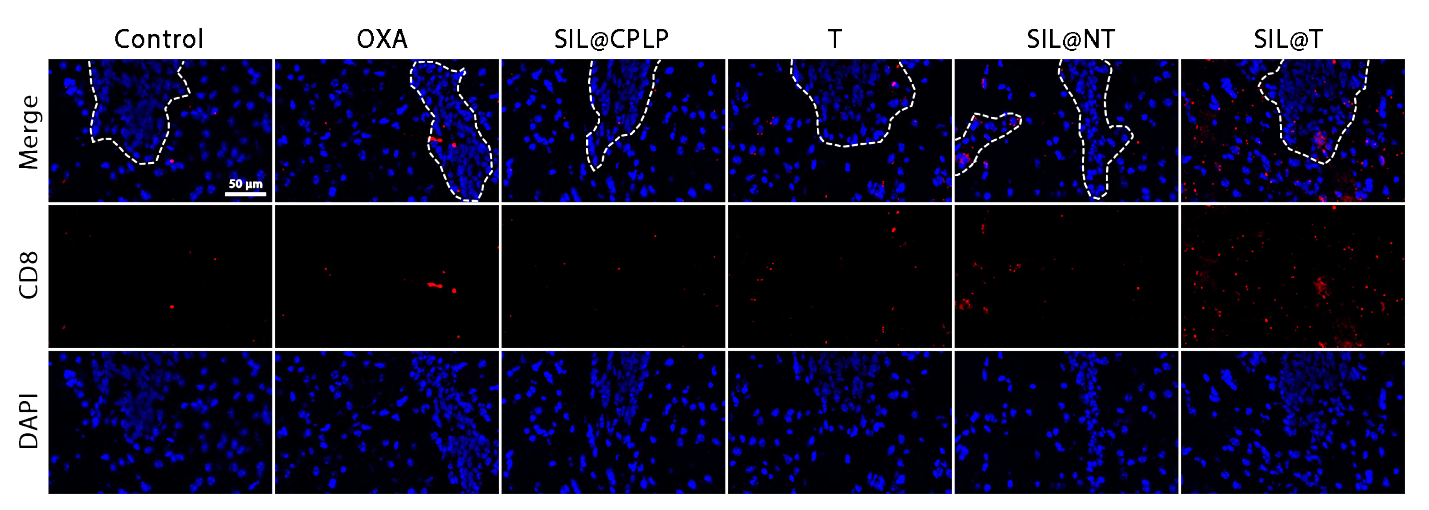
**

**Fig. S23.** Immunofluorescence imaging of CD8+ T cell infiltration in brain cryosections of BM-mice. Blue: nucleus; red: CD8. Scale bar: 50 μm.


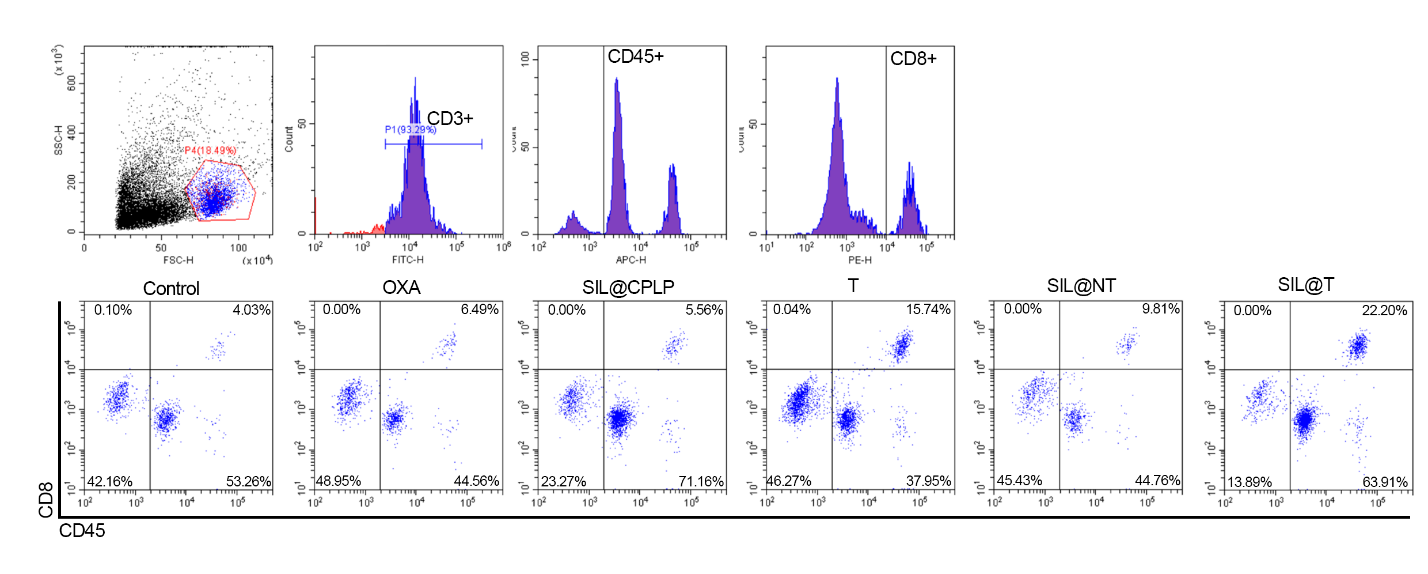


**Fig. S24.** Flow cytometry analysis of T cell polarization in brain metastases.


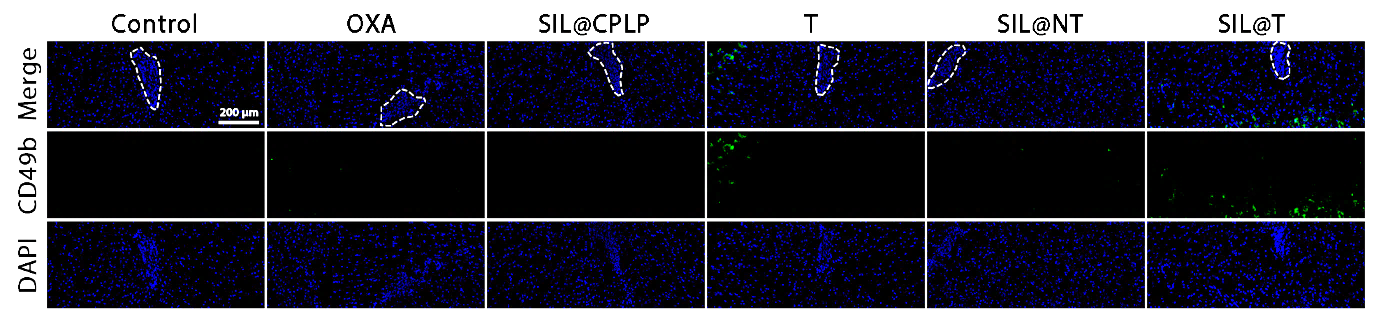


**Fig. S25.** Immunofluorescence imaging of NK cell infiltration in brain cryosections of BM-mice. Blue: nucleus; green: CD49b. Scale bar: 200 μm.


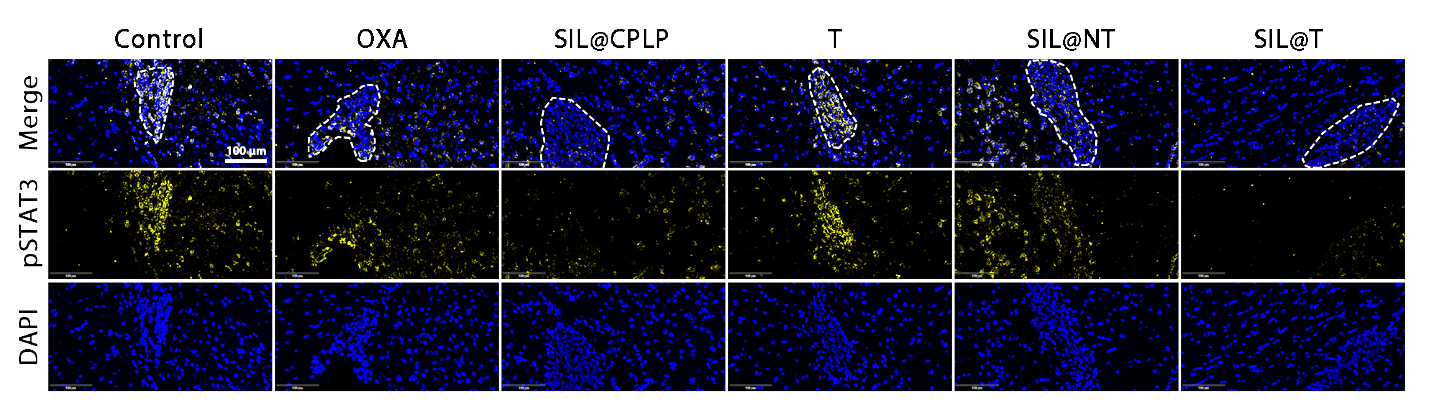


**Fig. S26.** Immunofluorescence imaging of pSTAT3 in brain cryosections of BM-mice. Blue: nucleus; yellow: pSTAT3. Scale bar: 100 μm.


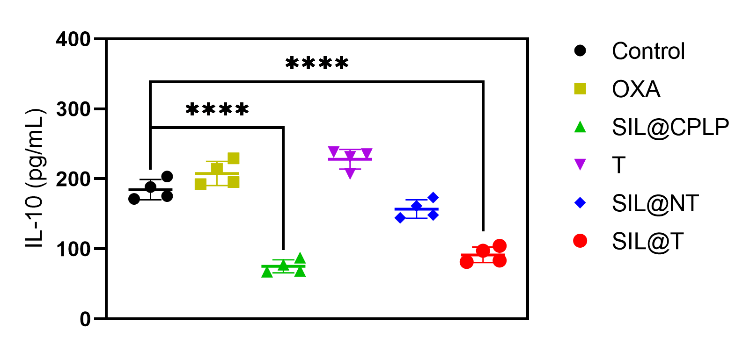


**Fig. S27.** ELISA results of IL-10 content in metastases (n=4, One-way ANOVA, ****P<0.0001).


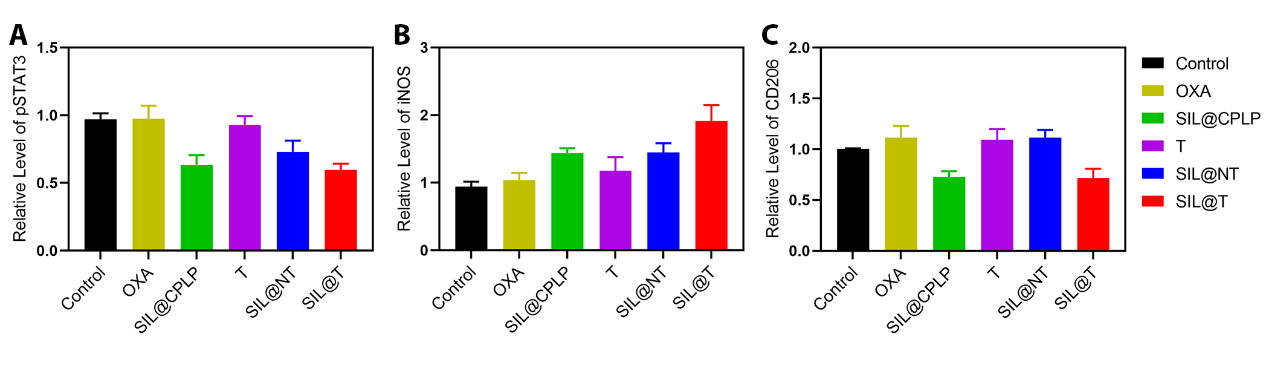


**Fig. S28.** WB quantification results of pSTAT3 (A), iNOS (B) and CD206 (C) in Figure 6D.


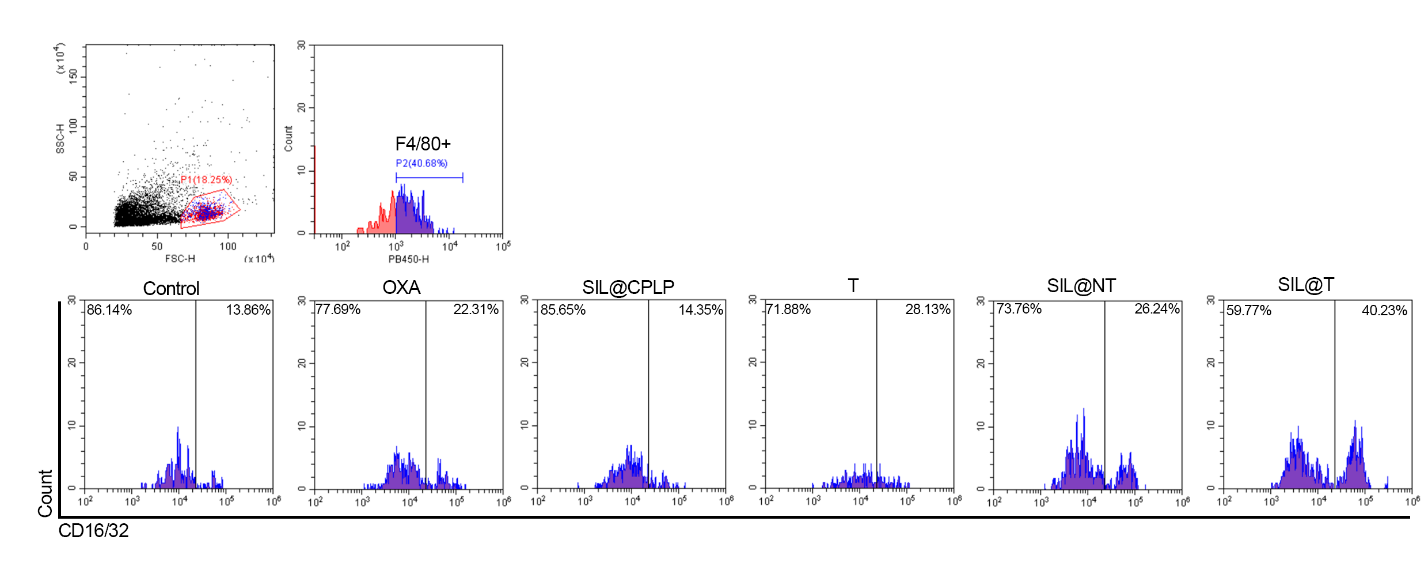


**Fig. S29.** Flow cytometry analysis of M1 polarization of TAM in brain metastases.


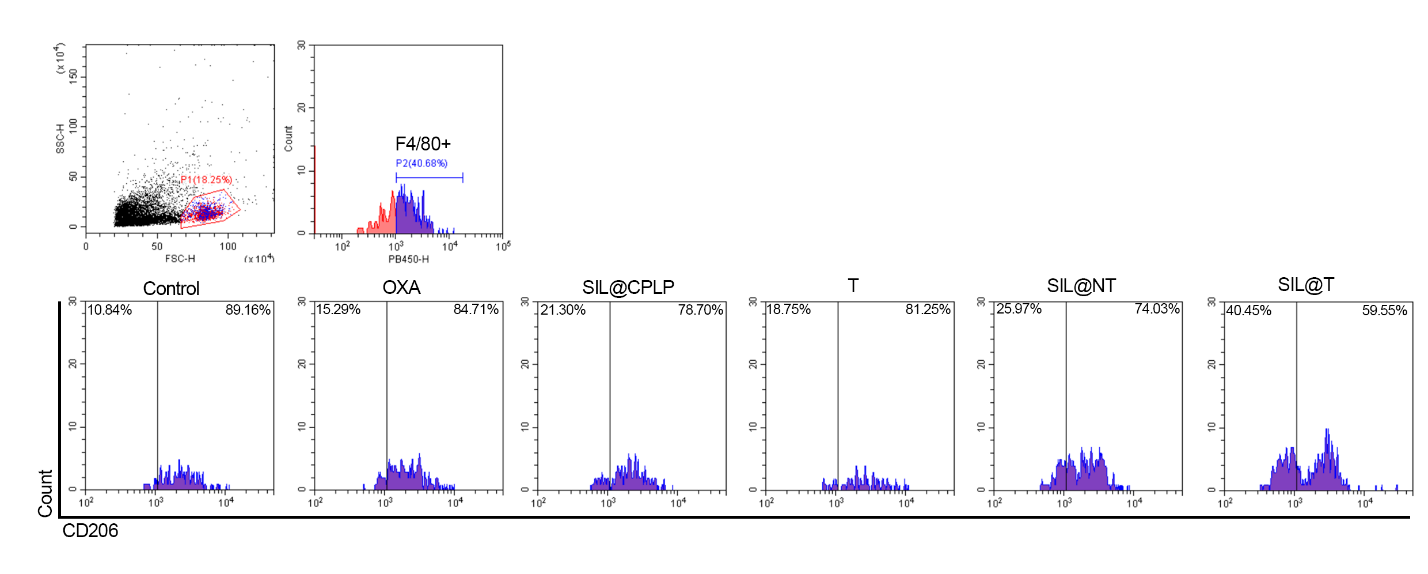


**Fig. S30.** Flow cytometry analysis of M2 polarization of TAM in brain metastases.


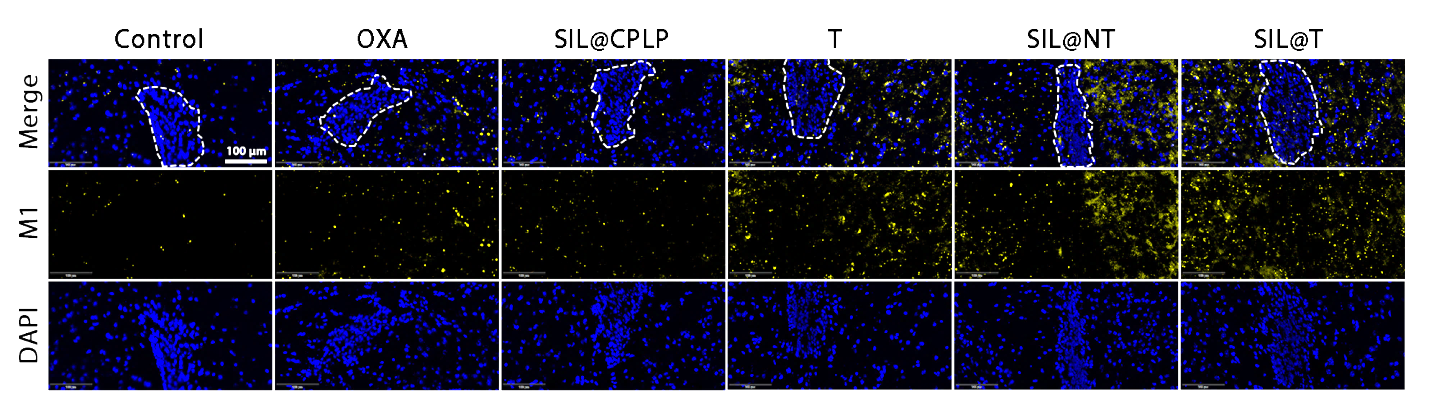


**Fig. S31.** Immunofluorescence imaging of CD16/32 in brain cryosections of BM-mice. Blue: nucleus; yellow: CD16/32. Scale bar: 100 μm.


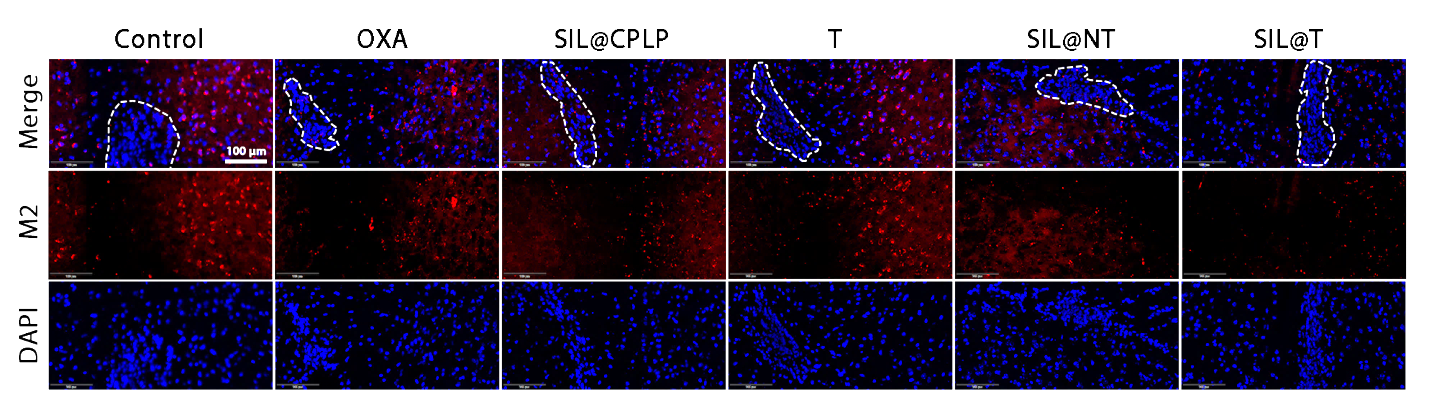


**Fig. S32.** Immunofluorescence imaging of CD206 in brain cryosections of BM-mice. Blue: nucleus; yellow: CD206. Scale bar: 100 μm.


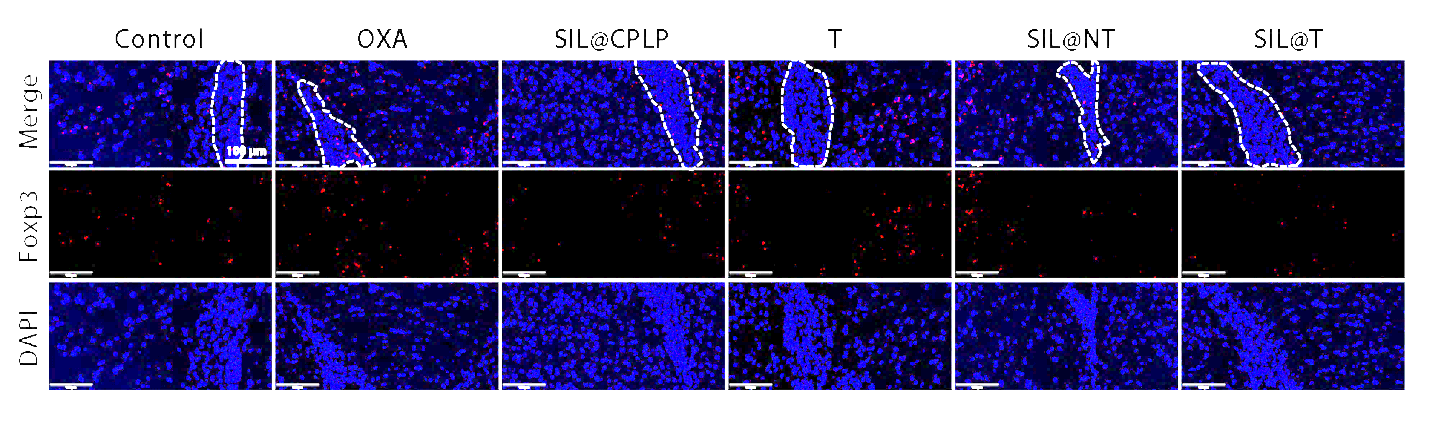


**Fig. S33.** Immunofluorescence imaging of CD8+ T cell infiltration in brain cryosections of BM-mice. Blue: nucleus; red: Foxp3. Scale bar: 100 μm.


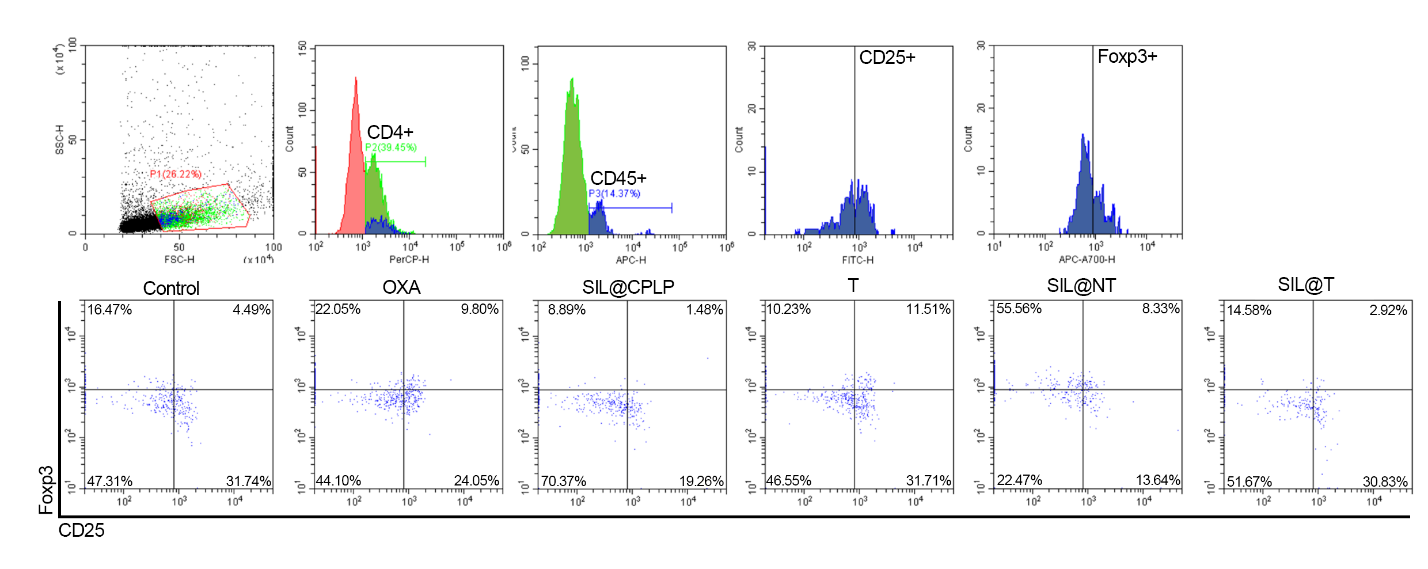


**Fig. S34.** Flow cytometry analysis of Treg cells in brain metastases.

| Micelle | Number Size  (nm) | Intensity  (nm) | PDI | Zeta Potential  (mV) |
| --- | --- | --- | --- | --- |
| T (PEG_5k_-Lys/OXA-Phe) | 86±1 | 159±13 | 0.215±0.031 | 4.2±0.1 |
| SIL@NT | 41±10 | 78±4 | 0.162±0.005 | 13.2±0.2 |
| SIL@T | 50±2 | 89±3 | 0.204±0.008 | 13.0±0.1 |
| PEG_2k_-Lys/OXA-Phe | 9±4 | 86±5 | 0.473±0.112 | -- |
| SIL@PEG_2k_-Lys/OXA-Phe | 6±3 | 79±1 | 0.451±0.096 | -- |

**Table S1.** Summary of the micelle properties. Data are presented as mean ± SD (n=3).

| Micelle | OXA | T | SIL@NT | SIL@T |
| --- | --- | --- | --- | --- |
| IC50 (μM) | 1.59±0.15 | 0.58±0.01 | 1.73±0.64 | 0.41±0.07 |

**Table S2.** IC_50_ of different micelles on 4T1-Br-Luc cells (n=6).

| Group | Control | OXA | SIL@CPLP | T | SIL@NT | SIL@T |
| --- | --- | --- | --- | --- | --- | --- |
| Median survival (d) | 21.0 | 25.0 | 24.0 | 27.5 | 26.5 | 32.5 |

**Table S3.** Median survival time of BM-mice (n=6).

| Group | Control | OXA | SIL@CPLP | T | SIL@NT | SIL@T |
| --- | --- | --- | --- | --- | --- | --- |
| Median survival (d) | 18.5 | 21.5 | 19.5 | 24.0 | 25.0 | 28.0 |

**Table S4.** Median survival time of Co-BM-mice (n=6).

EXPERIMENTAL SECTION/METHODS

**Materials and chemicals**

*N_6_*-cbz-*L*-Lysine (Lys(*Z*)), Hydrogen bromide 33 wt.% in Acetic acid (HBr/HOAc, 33%), 2-(7-Azabenzotriazol-1-yl)-*N,N,N',N'*-tetramethyluronium hexafluorophosphate (HATU, 98 %), *N,N*-diisopropylethylamine (DIPEA, 99 %), D_2_O (99.8 %), bovine serum albumin (BSA) were from J＆K Scientific (Shanghai, China). *L*-phenylalanine, triphosgene were from TCI (Asakawa, Japan). Azido-PEG-NHS ester (Mw=5 kD) was purchased from JenKem (Beijing, China). Methosy-PEG-amine (CH_3_O-PEG-NH_2_, Mw=5 kD) was from Seebio Biotech (Shanghai, China). Dichloromethane, anhydrous (DCM), tetrahydrofuran, anhydrous (THF), *N,N*-dimethylformamide (DMF) were from Acros Organics (State of New Jersey, USA). Oxaliplatin, heparin sodium, wortmannin (WTM), *D*-luciferin potassium salt, glutathione (GSH), cell counting kit-8(CCK-8) were purchased from Meilunbio (Dalian, China). Cuprous iodide (CuI), dimethyl sulfoxide- *d_6_* (DMSO-*d_6_* (D, 99.8 %), TMS (0.03 %)) were from Energy Chemical (Shanghai, China). Succinic anhydride (99 %), sodium ascorbate, trifluoroacetic acid (TFA), pyrene, HEPES were from Aladdin Chemistry (Shanghai, China). *D*-type CSKC peptide was purchased from QYAOBIO (Suzhou, China). Sterile cell culture flasks, six-well plates, sterile 15 mL/50 mL centrifuge tubes were from Corning (New York, USA). Disposable sterile dropper was from NEST (Wuxi, China). SnakeSkin^TM^ dialysis tubing (MWCO=3.5, 5, 8 kD), DAPI (No. D3571), CD11c-perCP-cyanine5.5 antibody (45-0114-80), CD80-436 antibody (62-0801-80), CD86-PE antibody(12-0862-82), CD3-FITC antibody (11-0032-82), CD45-APC antibody (17-0451-82), CD4-PerCP antibody (45-0042-82), CD8-PEantibody (12-0081-82), CD25-FITC antibody (53-0251-80), Foxp3-450 antibody (48-5773-80), F4/80-450 antibody (48-4801-80), CD16/32 antibody (12-0161-82), CD206 antibody (25-2061-82) were from Thermo Scientific (Waltham, USA). BODIPY-NHS 630/650 was from Lumiprobe (Hunt Valley, USA). Gelatin, filipin, chlorpromazine, poly-*D*-lysine (PDL, Mw=70 kD), dimethyl sulfoxide for cellular use were from Sigma Aldrich (Saint Louis, USA). Ultrasonic coupling agent was from Parker Laboratorie (New Jersey, America). Carbon-backed copper mesh was from Zhongjingkeyi (Beijing, China). DMEM, FBS, pen-strep (100×), *L*-glutamine solution (100×), MEM NEAA (100×), trypsin-EDTA solution (0.25%), trypsin solution (0.25 %), B-27^TM^ supplement (50×), goat serum were from Gibco (Carlsbad, USA). Isoflurane was from RWD (Shenzhen, China). Rabbit anti-CD31 antibody (ab28364), goat anti-rabbit IgG H&L (Alexa Fluor® 568) (ab175476), donkey anti-rabbit IgG H&L (Alexa Fluor® 647) (ab150075), goat anti-rat IgG H&L (Alexa Fluor® 555) (ab150114) were from Abcam (Cambridge America). Phospho-STAT3 (Tyr705) (Y705) monoclonal antibody (AP0070) was from ABclonal Technology Co., Ltd. (Wuhan, China). All other reagents were analytically pure and from, Sinopharm Chemical Reagent (Shanghai, China).

**Cell lines**

Brain capillary endothelial cells (BCEC) were kindly gifted by Professor Lou Jinning from the Institute of Clinical Medicine of China-Japan Friendship Hospital.

Mouse-derived triple-negative breast cancer brain metastasis-prone cell line 4T1-Br was kindly donated by Jiangbing Zhou, Professor of Biomedical Engineering at Yale University.

A stable transduced murine-derived triple-negative breast cancer brain metastasis-prone cell line 4T1-Br /Luc carrying a luciferase reporter gene was constructed and screened with CMV-Luc-PGK-Puro lentivirus from 4T1-Br cell line by Genomeditech (Shanghai, China).

Primary astrocytes and BCEC were extracted from mammary mouse brains with reference to literature methods.

**Animals**

SPF grade c57 nude mice (female, 6~8 weeks old, weight 18-20 g), SPF grade c57 suckling mice (female, within 24 h or 1-3 days after birth) were purchased from SLAC Laboratory Animal Co., Ltd (Shanghai, China).

All animal experiment operations have been approved by the experimental animal ethics committee of the school of pharmacy of Fudan University and follow relevant management regulations.

**Methods**

**2.1 Synthesis of polymer materials**

2.1.1 Synthesis of amino acid activation monomers

The *N*-cbz-*L*-lysine activation monomer Lys(*Z*)-NCA (a) and *L*-phenylalanine activation monomer Phe-NCA (b) were synthesized according to the route shown in Figure M1. Lys(*Z*) (3 g, 10.7 mmol, 1 equiv.) of and triphosgene (1.27 g, 4.28 mmol, 0.4 equiv.) were added into a 100 mL two-necked vial protected by Ar. 50 mL of anhydrous THF was added through a syringe and the reaction was stirred in an oil bath at 50 °C. When the liquid changed from a miscible state to a clarified one (about 1 h), the reaction was terminated. The solution was cooled to room temperature and then filtered, and the filtrate was added dropwise with stirring to 250 mL of hexane for precipitation at -20 °C for at least 1 h. The liquid was filtered under reduced pressure using a Buchner funnel and dried under vacuum to obtain a white solid product of 2.4 g (Lys(*Z*)-NCA, a). Phe-NCA (b) was obtained using the same method and identified using ^1^H NMR.


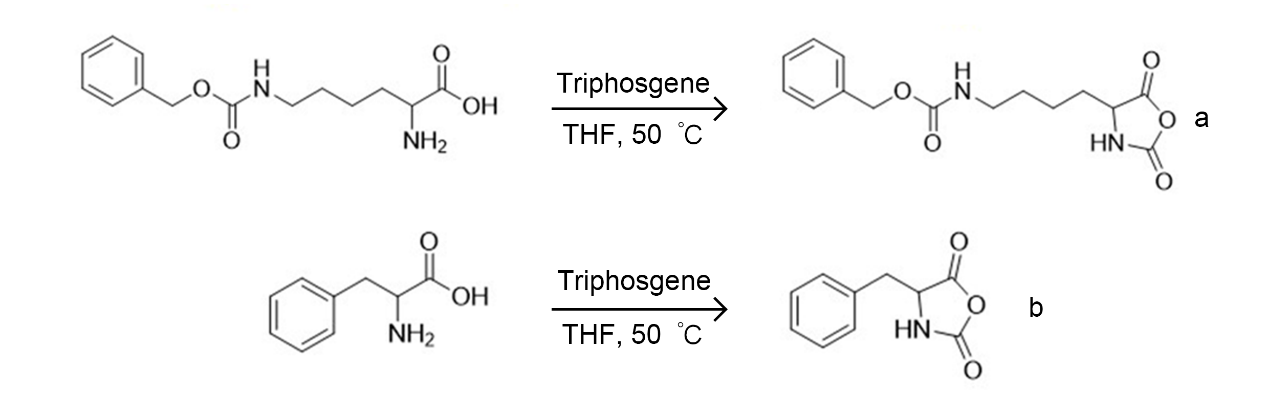


Figure M1. Synthesis of amino acid activation monomers.

2.1.2 The synthesis of Oxaliplatin prodrug (OXA-COOH)

OXA-COOH was synthesized according to the route shown in Figure M2. Oxaliplatin (1 g, 2.5 mmol) was weighed in a single-mouth flask, and 10 mL of 30 % aqueous hydrogen peroxide was added. Then the reaction was carried out at room temperature and protected from light for 12 h. The rest of hydrogen peroxide was removed by ultrasonication and freeze-dring to obtain a white powder (OXA-OH), which was identificated by ^1^H NMR.

OXA-OH (500 mg, 1.16 mmol, 1 equiv.), succinic anhydride (135 mg, 1.37 mmol, 1.18 equiv.), anhydrous triethylamine (TEA) (3.4μL, 0.05 mmol, 0.04 equiv.) were weighed in a two-mouth vial protected by Ar. Subsequently, 50 mL of anhydrous DMSO was added and the reaction was carried out at room temperature and protected from light for 24 h. Most of the DMSO was removed by rotary evaporation until about 2 mL of the solution remained, and the solid precipitate was precipitated by slowly dropping the remaining solution into 100 mL of anhydrous dichloromethane (DCM) and centrifuged at 5000 rpm for 5 min. Then the precipitate was dried under vacuum to obtain a white solid (OXA-COOH, c), which was identified by Mass spectrometry and ^1^H NMR.


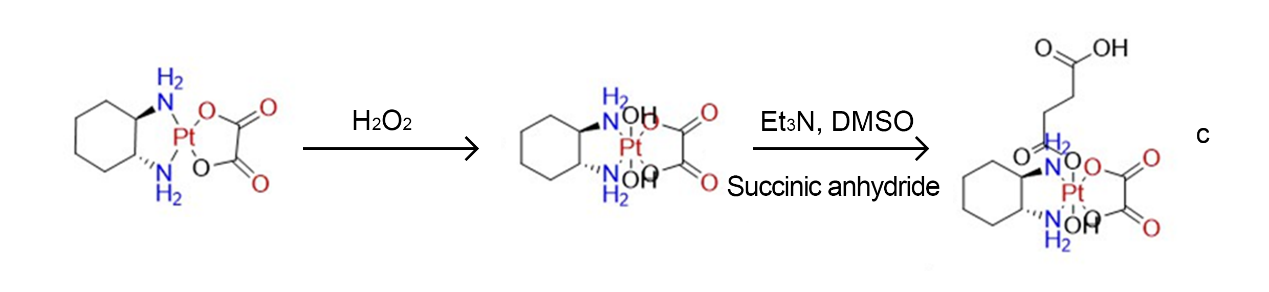


Figure M2. The synthesis of Oxaliplatin prodrug.

2.1.3 The synthesis of PEG-pLys/OXA-pPhe

PEG-pLys/OXA-pPhe was synthesized according to the route shown in Figure M3. mPEG-NH_2_ or N_3_-PEG-NH_2_(100 mg, 0.02 mmol, 1 equiv.) and Lys(*Z*)-NCA (61.25 mg, 0.2 mmol, 10 equiv.) were weighed in a two-mouth flask, dissolved by adding 10 mL of anhydrous DMF, and protected by Ar. After adding the anhydrous DMF solution (10 mL) of Phe-NCA (76.67 mg, 0.3 mmol, 15 equiv.), the reaction was stirred at 50 °C for 48 h. After the reaction mixture was cooled to room temperature, it was added to 200 mL of pre-chilled (-20 °C) anhydrous ether. Then the mixture was precipitated, filtered under reduced pressure using a brønsted funnel and dried under vacuum to obtain the white solid product. After adding the white solid to a single-mouth flask, 4 mL of TFA and 0.2 mL of HBr/AcOH were added sequentially, and the reaction solution was stirred at room temperature for 3 h. The reaction mixture was sealed in a dialysis bag (MWCO=3.5 kD) for 24 h and freeze-dried to obtain a white solid (d).

Oxaliplatin prodrug (OXA-COOH, 41.2 mg, 0.078 mmol, 4.5 equiv.) and HATU (31.37 mg, 0.078 mmol, 4.5equiv.) were weighed in a two-mouth flask. And DIPEA (10.71 μL, 0.078 mmol, 4.5 equiv.), 10 mL of anhydrous DMSO were added. The reaction was stirred at room temperature for 0.5 h in Ar. and under the protection from light. The above white solid(d) (140 mg, 0.0175 mmol, 1 equiv.), DIPEA (10.71 μL, 0.078 mmol, 4.5 equiv.) dissolved in 10 mL anhydrous DMSO were added to the above system and the reaction mixture was stirred at room temperature for 24 h in Ar and under the protection from light. Then the reaction mixture was sealed in a dialysis bag (MWCO=3.5 kD) for 24 h and freeze-dried to obtain a yellow solid. The products of each step were identified by ^1^H NMR.


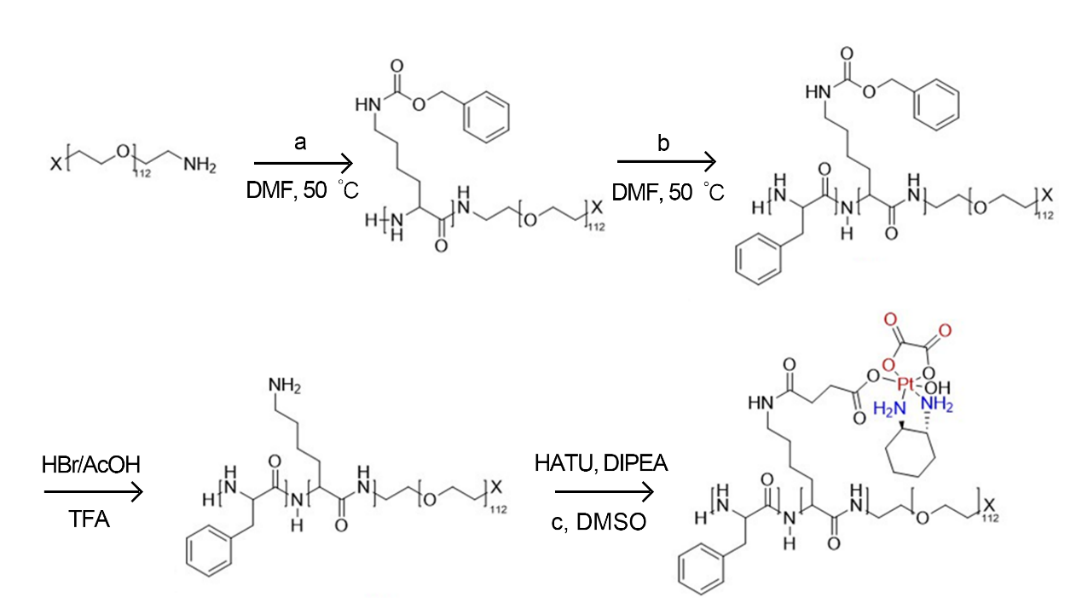


Figure M3. The synthesis of PEG-*p*Lys/OXA-*p*Phe.

2.1.4 Synthesis of CSKC modified polymer

CSKC-PEG-pLys/OXA-pPhe was synthesized by the monovalent copper-catalyzed Click reaction according to the route shown in Figure M4. The above yellow solid (110mg, 1 equiv.) was dissolved in 11 mL DMSO and dialyzed in water for 4 h. Then the DMSO was removed to obtain aqueous micellar solution. The hexynoic acid-modified CSKC peptide (10 mg, 2 equiv.), sodium ascorbate (9.4 mg, 5 equiv.) and anhydrous copper sulfate (2.4 mg, 1 equiv.) were added to the above micellar solution sequentially, and the reaction mixture was stirred for 24 h at room temperature and protected from light. Then the reaction mixture was sealed in a dialysis bag (MWCO=3.5 kD) and placed in PBS (pH=7.4) containing 10 mM EDTA-2Na. The reaction solution was sealed in a dialysis bag (MWCO=3.5 kD) and dialyzed in PBS (pH=7.4) with 10 mM EDTA-2Na for 12 h. The reaction mixture was then dialyzed in deionized water for 48 h and freeze-dried to obtain the white product which was identified by ^1^H NMR.


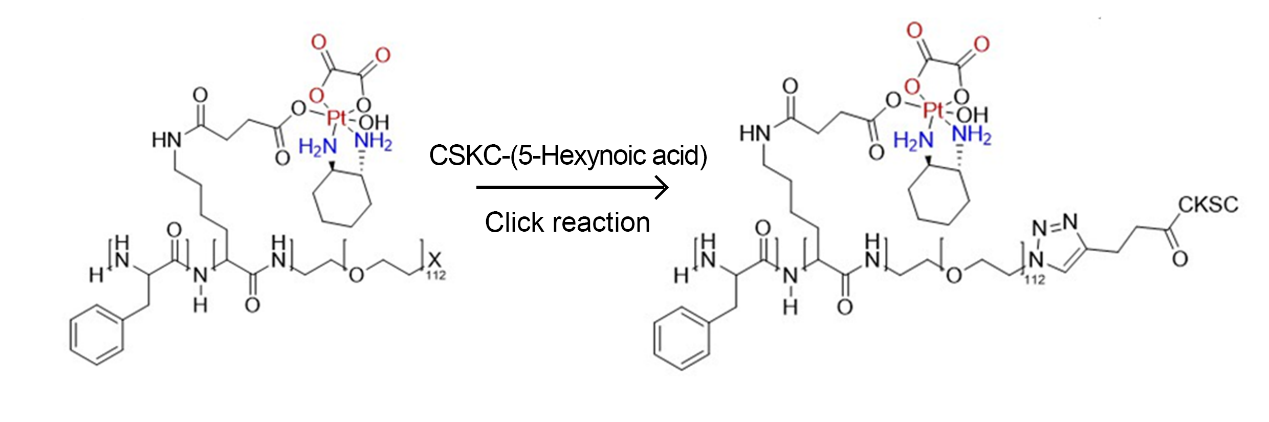


Figure M4. Synthesis of CSKC modified polymer.

**2.2 Preparation, characterizations and formulation optimization of the micelles**

2.2.1 Preparation and formulation optimization of the micelles

The micelles were prepared by a dialysis method. The polymer materials (PEG-*p*Lys/OXA-*p*Phe or CSKC-PEG-*p*Lys/OXA-*p*Phe) were dissolved in DMSO at a concentration of 10 mg/mL and were sealed in the dialysis bag (MWCO=3.5 kD. Then they were dialyzed in deionized water for 24 h with three water changes. The liquid in the dialysis bag was collected, and the particle size distribution and zeta potential of the micelles were measured using Malvern3600 Zetasizer Nano-ZS laser particle size analyzer (Malvern Panalytical, Worcestershire UK).

2.2.2 The redox-sensitive properties of polymeric micelles

The sensitivity of polymeric micelles was investigated using PBS (pH=7.4) buffer containing 10 mM Vc to simulate the cytoplasmic microenvironment and PBS (pH=5.5) buffer containing 2 mM Vc to simulate the lysosomal microenvironment. The drug release from the micelles was investigated by high performance liquid chromatography (HPLC) at different time points (OXA detection method: column: Agilent C18, 250 mm×4.6 mm, 5 μm; Mobile phase: 10% MeOH-90% H_2_O; Flow rate: 1 mL/min; injection volume: 10 μL; Detector and detection wavelength: VWD, 250 nm.) (SIL detection method: column: Agilent C18, 250 mm×4.6 mm, 5 μm; Mobile phase: 50% ACN-50% NaH2PO4 (0.1 M, pH=4.8); Flow rate: 1 mL/min; Injection volume: 10 μL; Detector and detection wavelength: VWD, 288 nm).

**2.3 Preparation of BODIPY-labeled micelles**

The near-infrared fluorescent probe BODIPY-NHS (Ex=630 nm/Em=650 nm) was labeled to the amino residue of the lysine side chain in the polymer material by an amidation reaction. Polymer materials (15 mg, 3 eq.) and BODIPY-NHS (0.37 mg, 1 eq.) were dissolved in anhydrous DMF at concentrations of 10 mg/mL and 1 mg/mL, respectively. The reaction solution was sealed in a dialysis bag (MWCO=3.5 kD) and dialyzed in DMF for 12 h to remove the free probe, and then exchanged to deionized water for 48 h. Dialyzed for 48 h and freeze-dried to obtain the BODIPY-labeled polymer material.

**2.4 Cell culture**

The complete medium formulation for 4T1-Br (4T1-Br/Luc or 4T1-Br/GFP) cells was: high-sugar DMEM medium with 10% (v/v) FBS (additional 0.5 μg/mL puromycin for 4T1-Br/Luc; additional 4 μg/mL puromycin for 4T1-Br/GFP).

The complete medium formulation for BCEC and primary BCEC was: high-sugar DMEM basal medium containing 20 mg/L sodium heparin, 2 mM glutamine, and 20% (v/v) heat-inactivated FBS. BCEC were inoculated using 2% gelatin precoated cell culture dishes.

Complete medium formulation for primary astrocytes is: high sugar DMEM medium containing B-27^TM^ Supplement, 10% (v/v) FBS (100 U/mL penicillin and 100 mg/mL streptomycin are required to be added to the medium for the first three days). Cell culture dishes are precoated using sterile PDL solution (0.1 mg/mL).to inoculate primary astrocytes.

**2.5 Extraction and culture of primary astrocytes and BCEC**

Primary astrocytes were extracted from the brains of suckling mice within 24 h of birth according to the literature method. 1) Prepare PDL precoated T25 culture flasks, surgical instruments (2 precision fine straight forceps, 2 small forceps, 1 iris scissors, 1 large forceps); 2) Anesthetize suckling mice on ice for 10 min, disinfect with 75% ethanol spray and transfer to ultra clean bench; 3) Break the neck, remove the whole brain tissue; 4) remove the cerebellum and olfactory bulb with small forceps and put the brain into a new sterile dish with Hank's; 5) open the two hemispheres along the midline, peel off the meninges with precision fine straight forceps and put the brain tissue into a new dish with Hank's; 6) After aspirating the excess liquid, the brain tissue was cut into chyme form with iris scissors; 7) Add 0.125% trypsin and placed in a 37 °C incubator for 15-20 min for digestion; 8) The suspension was centrifuged at 800 rpm for 5 min, and the supernatant was discarded; 9) 4 mL of complete medium was added and the cells were blown with a burette until they were dispersed. Single cell suspension was obtained after filtration through a 70 μm cell sieve; 10) Inoculate the single cell suspension with 1×10^6^/T25, change the suspension completely on the second and third days, and then half change the suspension every other day until the cells are full grown (about 7 days); 11) Screw the flask tightly, seal the sealing film, and shake at 200 rpm at 37 °C for 12-16 h; 12) Discard the medium, wash the cells 3 times with Hank' s and collect them for subsequent experiments.

Primary BCEC was extracted from the brains of suckling mice within 1-3 days of birth according to the literature method. 1) Prepare a 35 mm culture dish precoated with 2% gelatin and surgical instruments (2 precision fine forceps, 2 small forceps, 1 iris scissors, 1 large forceps). 2) Anesthetize the suckling mice on ice for 10 min, disinfect with 75% ethanol spray and transfer to the clean bench. 3) Break the neck and remove the whole brain tissue. 4) Remove the cerebellum and olfactory bulb with small forceps and put the brain into a new sterile dish with Hank's; 5) Open the two hemispheres along the midline, peel off the meninges with precision fine straight forceps and put the brain tissue into a new dish with Hank's. 6) After aspirating the excess liquid, the brain tissue was cut into chyme form with iris scissors; 7) Add 0.05% trypsin and place the culture dish in a 37 °C incubator for 30 min for digestion; 8) The suspension was centrifuged at 1000 rpm for 5 min, and the supernatant was discarded; 9) An equal amount of 15% dextrose was added, mixed, and centrifuged at 2000 rpm for 20 min; 10) Hank's was added to resuspend the cells. And pass the suspension through a 150 μm cell sieve. Then filter the filtrate through a 70 μm cell sieve and collect the microvascular segments on the sieve; 11) 1 mg/mL type II collagenase was added to digest the cells in an incubator at 37 °C for 30 min. Terminate the digestion, and centrifuge the suspension at 1000 rpm for 5 min; 12) Collect the cell precipitate and inoculate the precipitate containing 100 U/mL penicillin, 100 mg/mL streptomycin, and 20% (v/v) heat-inactivated FBS in high sugar DMEM basal medium in 35 mm culture dishes. Change the complete medium of primary BCEC on day 4; 13) After 10 days of culture, the cell clones derived from the vascular segments were retained and the rest of the cells were hung off with a cell scraper, and the remaining cell clones were collected for subsequent experiments.

**2.6 Uptake by cells**

2.6.1 Uptake of the micelles by 4T1-Br cells

The 4T1-Br cells were inoculated in six-well plates at a density of 1×10^5^/well, and when the cells reached 80-90% fusion (typically 18-24 h), the medium was replaced with 2 mL of basal DMEM medium containing dual fluorescent markers (BODIPY-labeled micelles, coumarin-6 used to mimic being encapsulated in hydrophobic core of SIL), SIL@NT or SIL@T in basal DMEM medium (equivalent to 4 μM SIL administered per well). The drug-containing basal medium was removed after 1 h incubation in a 37 °C incubator. And the cells were digested and collected, and 4T1-Br cell uptake was detected using a CytoFLEX S flow cytometer.

2.6.2 Investigation of the uptake pathway of CSKC-modified targeting micelles

The uptake pathway of CSKC-modified targeting micelles SIL@T was investigated using 4T1-Br cells. 4T1-Br cells were inoculated in six-well plates at a density of 1×10^5^/well and cultured until fusion reached 80-90% (typically 18-24 h). Each well was pretreated with a different endocytosis inhibitor for 1 h (niche protein endocytosis inhibitor philippin, 5 μg/mL; grid protein endocytosis inhibitor chlorpromazine, 5 μg/mL; giant cell drinking inhibitor worman penicillin, 1 μg/mL). Then the inhibitor-containing medium was removed and DMEM basal medium containing BODIPY-labeled SIL@T was added (each well was administered at a dose equivalent to 4 μM SIL). Then the cells were incubated in an incubator at 37 °C for 0.5 h. One sample (untreated with endocytosis inhibitor) was incubated in a refrigerator at 4 °C for 0.5 h. Cells were washed 3 times with Hank's, digested and collected, and 4T1-Br cell uptake was detected using a CytoFLEX S flow cytometer.

2.6.3 In vitro BBB penetration of CSKC-modified targeted micelles

1) BCEC were inoculated at a density of 5*10^4^ cells/cm2 in a 2% gelatin pre-coated 24-well plate transmembrane insert for 14 days; 2) An appropriate amount of culture medium was added to the donor pool so that a liquid level difference of greater than 5 cm could be formed between the donor and recipient pools. Then the cells were incubated for 4 h. If the donor and recipient pools still maintain a significant liquid level difference, BBB formation is tentatively identified; 3) The culture medium was removed and 250 μL of basal DMEM medium containing 0.6 μCi 14C-sucrose, BODIPY-labeled SIL@NT or SIL@T (each well was administered at a dose equivalent to 4 μM SIL) was added separately to each well. 4) The plate was placed in a shaker at 37 °C with 50 rpm shaking and sampled 20 μL at 5, 10, 15 and 30 min; 5) The samples were divided into two parts with one for measuring the 14C-sucrose (add scintillation solution, mix and leave overnight, and measure the radioactivity count by liquid flash counter), and the other one for measuring the BBB permeation of micelles.

**2.7 In vivo targeting of SIL@T to brain metastases**

2.7.1 Establishment of the BM-mice

The BM-mice was established by ultrasound-guided left ventricular injection technique with reference to the literature method [26]. The c57 mice (hair removal cream to remove the mouse's chest coat, 18-20 g) were anesthetized using an isoflurane-oxygen (or air) gas anesthesia system, and the anesthetized c57 mice were fixed in a supine position on an imaging table (set at 37 °C) using breathable medical tape. The mouse's chest was coated with ultrasound coupling agent gel (to avoid air bubbles), and the imaging table and probe position were adjusted so that the left ventricular view was clearly presented in the field of view. A syringe aspirated with 4T1-Br/Luc cell suspension was inserted between the probe and the gel in a direction parallel to the probe to pierce the skin and muscle layers into the left ventricle (move the syringe slowly, do not insert too fast or too deep) and 125 μL DPBS suspension containing 4×10^5^ 4T1-Br/Luc cells was slowly injected.

After 1 week of modeling, each c57 mouse was injected intraperitoneally with 100 μL of saline containing 30 mg/mL of potassium D-fluorescein, and the IVIS Spectrum (Small Animal In Vivo Optical Imaging System) was used to monitor the bioluminescence signal of 4T1-Br/Luc cells and confirm the successful establishment of the BM-mice.


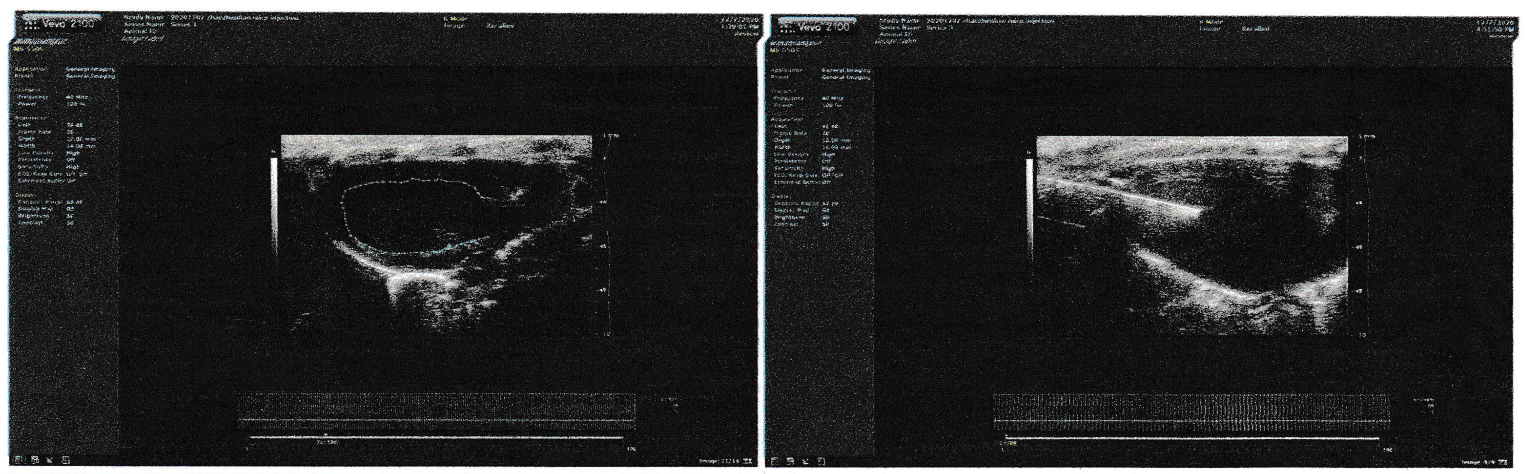


Figure M5. Establishment of the BM-mice by ultrasound-guided left ventricular injection technology.

2.7.2 Targeting ability of SIL@T to TNBC brain metastases

BM-mice with comparable brain metastasis Luc signals were selected and randomly divided into 2 groups of 3 animals each, and BODIPY-labeled micelles SIL@NT and SIL@T (dose: 0.5 mg BODIPY/kg) were injected via tail vein, respectively. The internal distribution of micelles was investigated by live imaging of small animals at different time points.

Double fluorescence-labeled micelles SIL@NT and SIL@T were obtained by encapsulating coumarin-6 with BODIPY-labeled micelles. Brain tissue was taken out 2 h after tail vein injection (dose: 0.5 mg BODIPY/kg) and frozen sections were prepared (light-protected operation). Neovascularization was labeled using Anti-CD31antibody (1:50 dilution) and goat anti-rabbit IgG H&L (Alexa Fluor® 568) secondary antibody (1:1000 dilution), and nuclei were labeled with DAPI. The prepared tissue samples were imaged and observed using an inverted laser confocal microscope.

The excitation wavelength (Ex) and emission wavelength (Em) ranges of each channel were set as follows (without interfering with each other).

DAPI：Ex=405 nm/Em=415~467 nm；

Alexa Fluor® 488：Ex=488 nm/Em=502~550 nm；

Cy3：Ex=552 nm/Em=564~625 nm；

BODIPY：Ex=638 nm/Em=650~755 nm

2.7.3 Establishment of the Co-BM-mice

The Co-BM-mice was established with reference to the literature method [56]. 4T1-Br-Luc cells were injected subcutaneously into the mammary gland to generate extracranial tumors (5×10^5^ 4T1-Br/Luc cells). To generate intracranial tumors, cancer cells (2×10^5^ 4T1-Br/Luc cells) were stereotactically injected into the striatum (2-mm right from the midline, 2-mm anterior from bregma, 3-mm deep) after 3 day .

After 2 day of modeling, each c57 mouse was injected intraperitoneally with 100 μL of saline containing 30 mg/mL of potassium D-fluorescein, and the IVIS Spectrum (Small Animal In Vivo Optical Imaging System) was used to monitor the bioluminescence signal of 4T1-Br/Luc cells and confirm the successful establishment of the Co-BM-mice.

**2.8 Investigation of in vitro cytotoxicity of micelles on TNBC**

The toxicity of micelles to TNBC cells 4T1-Br was detected using the CCK-8 kit . Refer to the kit instructions for the specific method.

**2.9 In *vitro* evaluation of the efficacy of SIL@T**

2.9.1 Experimental grouping of formulations

The formulations include: G1: PBS; G2: OXA; G3: SIL@CPLP (where CPLP stands for the targeted polymeric material without OXA attached and SIL@CPLP stands for a micelle constructed from CPLP loaded with SIL.); G4: T (where T stands for a micelle constructed from the targeted polymeric material attached to OXA.); G5: SIL@NT (where NT stands for a micelle constructed from the non-targeted polymeric material attached to OXA. SIL@NT represents the loading of SIL into NT.); G6: SIL@T (where T stands for a micelle constructed from the targeted polymeric material attached to OXA. SIL@T represents the loading of SIL into T.). Among them, G1 was used as a blank control; G4 was a targeted prodrug form of G2 to demonstrate that T could enhance the stability of OXA as well as increase its accumulation at the target site; G5 was a non-targeted form of G6 to confirm that modification of CSKC could help micelles to better cross the BBB and further target to metastases; G3 and G4 were single-drug controls of G6 to demonstrate that the combination of the two drugs could achieve better metastatic tumor treatment by a cocktail-like strategy.

2.9.2 Drug administration

Cultures of primary astrocytes (24 h) and complete DMEM cultures were mixed 1:1 for the culture of 4T1-Br-Luc cells.When the cell confluence reached 80~90%, different preparations (100 μmol per group of OXA; 80 μmol per group of SIL) were given and incubated with the cells for 4 hours, and then replaced with fresh medium to continue culturing for 72 h.

2.9.3 The inhibitory effect of SIL@T on STAT3 phosphorylation

1) The cell was lysed in RIPA buffer containing protease inhibitors, and the lysate solution was centrifuged at 12,000 rpm for 10 min at 4 °C, and the supernatant was collected to obtain proteins; 2) After the protein concentration was quantified by BCA assay, each group used 20 μg protein samples in SDS-PAGE gradient gel (4-20%) for separation (constant pressure, 100 V) and wet transfer to PVDF membrane at 300 mA constant flow; 3) The PVDF membrane was washed with TBS containing 0.1% Tween-20 (TBST) and blocked with TBST containing 5% skim milk for 1 h; 4) Discard the liquid, and add TBST,. The PVDF membranes were washed three times for 5 min each on a 70 rpm shaker and incubated overnight at 4°C in the corresponding antibody dilutions (pSTAT3: 1/2000); 5) The liquid was discarded, and TBST was added. The PVDF membranes were washed three times for 15 min each on a 70 rpm shaker, and the corresponding horseradish peroxidase (HRP)-coupled secondary antibodies (goat anti-mouse IgG: 1/1000) were incubated with PVDF membranes; 6) Discard the liquid and add TBST. The PVDF membrane were washed three times on a 70 rpm shaker for 15 min each time, and ECL developer was added to the gel imaging system to acquire images.

2.9.4 The inhibitory effect of SIL@T on HMGB1/CRT

When using WB to analyze: 1) The culture broth was collected and centrifuged at 12,000 rpm for 10 min at 4 °C, and the supernatant was collected to obtain proteins; 2) After the protein concentration was quantified by BCA assay, each group used 20 μg protein samples in SDS-PAGE gradient gel (4-20%) for separation (constant pressure, 100 V) and wet transfer to PVDF membrane at 300 mA constant flow; 3) The PVDF membrane was washed with TBS containing 0.1% Tween-20 (TBST) and blocked with TBST containing 5% skim milk for 1 h; 4) Discard the liquid, and add TBST,. The PVDF membranes were washed three times for 5 min each on a 70 rpm shaker and incubated overnight at 4°C in the corresponding antibody dilutions (HMGB1: 1/10000); 5) The liquid was discarded, and TBST was added. The PVDF membranes were washed three times for 15 min each on a 70 rpm shaker, and the corresponding horseradish peroxidase (HRP)-coupled secondary antibodies (goat anti-rabbit IgG: 1/1000) were incubated with PVDF membranes; 6) Discard the liquid and add TBST. The PVDF membrane were washed three times on a 70 rpm shaker for 15 min each time, and ECL developer was added to the gel imaging system to acquire images.

When using laser confocal live cell imaging system to observe the CRT and HMGB1: use 4% paraformaldehyde to fix the cells. Use Anti-CRT/HMGB1 primary antibody and goat anti-rabbit IgG H&L (Alexa Fluor® 555) secondary antibody (1:1000 dilution) to fluorescently label CRT/HMGB1 protein. And then use DAPI (5 μg/mL) to counter-stain the nuclei for 15 minutes.

**2.10 In vivo evaluation of the efficacy of SIL@T on brain metastases from TNBC**

2.10.1 Experimental grouping of formulations

The formulations include: G1: Saline; G2: OXA; G3: SIL@CPLP; G4: T; G5: SIL@NT; G6: SIL@T

2.10.2 Administration

On the seventh day after modeling (i.e., grouping day -6), brain metastasis Luc signals were monitored by IVIS Spectrum Small Animal Live Imaging System and BM-mice were randomly divided into 6 groups according to the signals. On days 1, 5 and 9 after grouping, each group was injected via tail vein with a dose of OXA equivalent to 5 mg/kg and a dose of SIL equivalent to 6 mg/kg.

2.10.3 Survival status, brain metastasis signal and body weight monitoring in BM-mice

The survival of each group of BM-mice was checked daily after drug administration, and the mice were weighed and monitored every 4 days starting from the first day of grouping. GraphPad Prism 8.0 was used to plot the weight change curve, brain metastasis signal change curve and survival curve, and one-way ANOVA was used to analyze the weight curve and brain metastasis signal curve, and survival test was used to analyze the survival curve and compare the differences between groups.

**2.11 Investigation of the therapeutic mechanism of SIL@T on TNBC brain metastases**

Successfully established BM-mice were given formulations of each group on days 1, 5, grouped as in 2.9.1, and brain tissue was taken out on day 6 for flow cytometry and preparation of frozen sections to investigate the mechanism of SIL@T treatment of brain metastasis.

2.11.1 Investigation of the mechanism of SIL@T activation of immune response to TNBC brain metastases

2.11.1.1 Frozen sections were used to investigate CRT translocation of tumor cells in brain metastases

1) Sections were washed twice for 5 min each in TBS solution containing 0.025% Triton X-100 (pH=7.4); 2) The fluid around the tissue was gently wiped away using filter paper, and closed circles were constructed around the brain tissue with an immunohistochemical pen for antibody staining; 3) The brain tissue was blocked at room temperature for 2 h using TBS solution containing 10% goat serum and 1% BSA; 4) Gently wipe away the liquid around the tissue using filter paper, add 50 μL of CRT antibody diluent (diluent is TBS containing 1% BSA, 1:500 dilution) to each brain tissue and incubate at 4 °C overnight; 5) Remove the sections, restore to room temperature, and wash them with TBS solution three times for 15 min each time; 6) Gently wipe away the liquid around the tissue with filter paper, add 50 μL of donkey anti-rabbit IgG H&L (Alexa Fluor® 647) secondary antibody dilution (diluted in TBS, 1:1000 dilution) to each slice and incubate at 37 °C for 2 h; (7 Wash 3 times with TBS solution for 15 min each time; (8 Restain the cell nuclei with DAPI (5 μg/mL) for 15 min. (9 Wash three times with TBS solution for 5 min each time; (10 Seal the slice with anti-fluorescence quenching sealer and scan the whole slice under Olympus VS120 section scanning system (fluorescence channel: DAPI, Em=455 nm; Cy5, Em=670 nm.) .

2.11.1.2 Frozen sections were used to investigate CD8+ T infiltration in brain metastases

The basic operation is the same as in 2.10.1.1, where steps 4-7 were replaced by using filter paper to gently wipe away the fluid around the tissue, adding 50 μL of CD3, CD8 direct antibody dilution (dilution solution is TBS containing 1% BSA, 1:500 dilution) to each slice of brain tissue, incubating overnight at 4 °C, removing the slice, restoring it to room temperature, and washing 3 times with TBS solution for 15 min each time (channel: Cy3, Em=565 nm; DAPI/FITC as 2.10.1.1)

2.11.1.3 Brain tissue homogenates were used to investigate ICD marker expression, and the expression levels of CRT and HMGB1 in metastatic foci were analyzed using protein blotting

1) Tissues were lysed in RIPA buffer containing protease inhibitors, and the lysate solution was centrifuged at 12,000 rpm for 10 min at 4 °C, and the supernatant was collected to obtain proteins; 2) After the protein concentration was quantified by BCA assay, each group used 20 μg protein samples in SDS-PAGE gradient gel (4-20%) for separation (constant pressure, 100 V) and wet transfer to PVDF membrane at 300 mA constant flow; 3) The PVDF membrane was washed with TBS containing 0.1% Tween-20 (TBST) and blocked with TBST containing 5% skim milk for 1 h; 4) Discard the liquid, and add TBST,. The PVDF membranes were washed three times for 5 min each on a 70 rpm shaker and incubated overnight at 4°C in the corresponding antibody dilutions (CRT: 1/5000; HMGB1: 1/10000); 5) The liquid was discarded, and TBST was added. The PVDF membranes were washed three times for 15 min each on a 70 rpm shaker, and the corresponding horseradish peroxidase (HRP)-coupled secondary antibodies (goat anti-rabbit IgG: 1/1000; goat anti-mouse IgG: 1/1000) were incubated with PVDF membranes; 6) Discard the liquid and add TBST. The PVDF membrane were washed three times on a 70 rpm shaker for 15 min each time, and ECL developer was added to the gel imaging system to acquire images.

2.11.1.4 Tissue flow cytometry was used to investigate peripheral immune activation

1) Remove tissue (lymph and spleen) after PBS perfusion. Grind, and filter it through a 70 μm cell sieve; 2) Centrifuge it at 1000 rpm for 5 min. Discard supernatant and add PBS to resuspend cells and count; 3) Take 200 μL of cell suspension containing approximately 10^6^ cells per sample, and add 5 μL of 0.5 mg/mL BSA solution. The cells were blocked at room temperature for 10 min; 4) The mixed antibody was added. The cells incubated for 20 min at room temperature and were protected from light; 5) The cells were centrifuged at 500 g for 5 min. Discard the supernatant and resuspend the cells in PBS. Then detect and analyze them using CytoFLEX S flow cytometer. (DC：CD11c、CD80、CD86；CD8+ T：CD3、CD45、CD8)

2.11.1.5 Tissue flow cytometry was used to investigate the immune infiltration of brain metastases

1) Remove brain tissue after PBS perfusion. Weigh 40 mg of brain tissue with metastases. Grind, and filter it through a 70 μm cell sieve. 2) Cell suspension was centrifuged at 1000 rpm for 5 min. Then discard the supernatant and add 3 mL of 30% Percoll. Blow to disperse the cells; 3) Add the suspension slowly to 3 mL of 70% Percoll upper layer to form a stratified layer (Centrifuge tubes containing the cells were washed in advance with FBS); 4) The tubes were centrifuges at 1800 rpm for 30 min at room temperature; 5) Aspirate the cells located at the stratified interface. PBS was added and centrifuged at 1200 rpm for 5 min. Resuspend the cells in PBS and count. Subsequent operations are the same as 3-5 in 2.10.1.4.

2.10.2 Investigate the mechanism of SIL@T to reverse the immunosuppressive microenvironment of TNBC brain metastasis

2.11.2.1 Frozen sections were used to investigate the STAT3 activation in brain metastatic focal tumor cells

The basic operation was the same as 2.10.1.1, where the antibody was pSTAT3, diluted 1:100.

2.11.2.2 Frozen sections were used to investigate the infiltration of Treg and M1/2 microglia/macrophages in brain metastases

The basic operation was the same as 2.10.1.2 with the HRP-conjugated antibody CD45, 1:500 dilution; Foxp3, 1:100 dilution; CD16/32, 1:800 dilution; CD206,1:200 dilution. (Channel: Cy5, Em=670 nm; the rest was the same as 2.10.1)

2.11.2.3 Brain tissue homogenates were investigated for STAT3 activation levels and microglial/macrophage marker expression

pSTAT3, iNOS and CD206 expression levels in metastatic foci were analyzed using protein blotting. The basic operation is the same as 2.10.1.3 with antibody pSTAT3, 1:2000 dilution; iNOS, 1:1000 dilution; CD206, 1:21000 dilution.

2.11.2.4 The measurement of TGF-β levels in brain tissue homogenates

1) Tissues were lysed in RIPA buffer containing protease inhibitors, and the lysate solutions were centrifuged at 12,000 rpm for 10 min at 4 °C. The supernatants were collected to obtain proteins; 2) TGF-β concentrations were measured using the TGF-β cytokine kit, referring to the kit instructions for details.

2.11.2.5 Tissue flow cytometry was used to investigate the infiltration of Treg and M1/2 microglia/macrophage in brain metastases

The basic operation is the same as 2.10.1.5 with M1: F4/80, CD16/32; M2: F4/80, CD206; Treg: CD4, CD45, CD25, Foxp3.

**2.12 HE staining**

Tissues of the heart, liver, spleen, lung, and kidney of BM-mice in 2.10 were taken out. Paraffin sections (5 μm thick) were prepared and stained using the HE staining kit. Sections were placed under an inverted fluorescent microscope in bright field for observation.

**2.13 Statistical analysis**

All data were analyzed by GraphPad Prism Version 8.0 (GraphPad Software, San Diego, USA) and presented as means ± standard deviation (SD). The significance level was defined as **P* < 0.05, ***P* < 0.01, ****P* < 0.001 and *****P* < 0.0001.
